# Synthesis, Computational Studies, and Structural Analysis of 1-(3,5-Dimethoxyphenyl)azetidin-2-ones with Antiproliferative Activity in Breast Cancer and Chemoresistant Colon Cancer

**DOI:** 10.3390/ph18091330

**Published:** 2025-09-05

**Authors:** Azizah M. Malebari, Shubhangi Kandwal, Abdirahman Ali, Darren Fayne, Brendan Twamley, Daniela M. Zisterer, Mary J. Meegan

**Affiliations:** 1 Department of Pharmaceutical Chemistry, College of Pharmacy, King Abdulaziz University, Jeddah 21589, Saudi Arabia; amelibary@kau.edu.sa; 2 Molecular Design Group, School of Chemical Sciences, Dublin City University, D09 V209 Dublin, Ireland; shubhangi.kandwal@dcu.ie (S.K.); abdirahman.ali8@mail.dcu.ie (A.A.); darren.p.fayne@dcu.ie (D.F.); 3 Molecular Design Group, School of Biochemistry and Immunology, Trinity Biomedical Sciences Institute, Trinity College Dublin, D02 R590 Dublin, Ireland; 4 DCU Life Sciences Institute, Dublin City University, D09 V209 Dublin, Ireland; 5 School of Chemistry, Trinity College Dublin, D02 P3X2 Dublin, Ireland; twamleyb@tcd.ie; 6 School of Biochemistry and Immunology, Trinity Biomedical Sciences Institute, Trinity College Dublin, D02 R590 Dublin, Ireland; dzistrer@tcd.ie; 7 School of Pharmacy and Pharmaceutical Sciences, Panoz Institute, Trinity College Dublin, D02 PN40 Dublin, Ireland

**Keywords:** 1-(3,5-dimethoxyphenyl)-2-azetidinone, *β*-lactam, antiproliferative activity, breast cancer, colon cancer, tubulin, single-crystal X-ray crystallography, docking, molecular dynamics

## Abstract

**Background/Objectives**: A series of 1-(3,5-dimethoxyphenyl)azetidine-2-ones were synthesised to evaluate their antiproliferative activity in MCF-7 breast cancer cells and HT-29 chemoresistant colon cancer cells. The 1,4-diarylazetidin-2-ones were designed by replacing the characteristic 3,4,5-trimethoxyphenyl Ring A of the antimitotic stilbene combretastatin CA-4 with a 3,5-dimethoxyphenyl substituent at N-1, together with phenyl, hydroxyl, and phenoxy substituents at C-3 of the four-membered ring. **Methods**: A panel of 12 novel compounds was synthesized and evaluated in estrogen receptor (ER)- and progesterone receptor (PR)-positive MCF-7 breast cancer cells followed with the more potent compounds further evaluated in HT-29 chemoresistant colon cancer cells. Cytotoxicity was determined by LDH assay. The structures of the 1-(3,5-dimethoxyphenyl)azetidine-2-ones **12i**, **12k**, **12o**, **12p** together with the 1-(3,5-dimethoxyphenyl)azetidine-2-one **12s** were determined by X-ray crystallography. The *trans* configuration of the C-3 and C-4 substituents of the *β*-lactam ring was confirmed for compounds **12k** and **12u**. Molecular modelling and molecular dynamics studies examined the molecular interactions of the compounds with the colchicine binding site of tubulin. **Results**: The 1-(3,5-Dimethoxyphenyl)-4-(4-ethoxyphenyl)-3-hydroxyazetidin-2-one **12l** was identified as the most potent antiproliferative compound in the series (with an IC_50_ value of 10 nM in MCF-7 breast cancer cells and 3 nM in HT-29 colon cancer cells) and with greater potency than CA-4 in the chemoresistant HT-29 cells. Computational docking studies predicted binding conformations for **12l** and the related series of compounds in the colchicine binding site of tubulin and rationalised the impact of the 3,5-dimethoxyphenyl substituent at N-1 of the azetidine-2-one on activity. **Conclusions**: These findings indicate that the novel 1-(3,5-dimethoxyphenyl)-2-azetidinone **12l** is a suitable candidate for further investigation as a potential antiproliferative microtubule-targeting agent for breast and chemoresistant colon cancers.

## 1. Introduction

Breast cancer is the most commonly occurring cancer in women and is the leading cause of female cancer deaths [1]. Breast cancer is a multifactorial disease, which is clinically defined by hormone receptor (HR) status, e.g., an estrogen receptor-positive (ER+) and/or progesterone-receptor positive (PR+) and human epidermal growth factor receptor 2-positive (HER2+) status [2]. Approximately 80% of breast cancers are classified as HR+/HER2− and are treated with adjuvant hormone therapies such as the selective estrogen receptor modulator (SERM) tamoxifen and aromatase inhibitors such as anastrozole [2]. Cyclin-dependent kinase 4 and 6 (CDK4/6) inhibitors, such as palbociclib, abemaciclib, and ribociclib are also effective [3], and can be combined with endocrine therapies such as the selective estrogen receptor degrader (SERD) fulvestrant for metastatic disease [4]. HER^2^+ targeted therapies include the monoclonal antibodies trastuzumab, pertuzumab, and margetuximab and the antibody–drug conjugates (ADCs) emtansine and trastuzumab deruxtecan. The targeted HER2 tyrosine kinase inhibitors neratinib and tucatinib [5] are also effective treatment options [6,7]. Triple-negative breast cancers (TNBCs) do not express ER or PR and are HER2 negative; they account for 15–20% of all breast cancer cases diagnosed [8,9]. While chemotherapy drugs can be effective for early stage TBNC, metastatic TNBC treatments include the immunotherapy drug pembrolizumab [10] and the antibody–drug conjugate (ADC) sacituzumab govitecan [11]; the poly(ADP-ribose)polymerase (PARP) inhibitor olaparib is a targeted therapy for women with *BRCA* gene mutations [12]. Improved therapeutic approaches are required for breast cancer patients to maximise efficacy and outcomes, while minimizing the side effects of the treatments [13].

Colorectal cancer (CRC) is the third most frequently diagnosed cancer worldwide [1,14]. Colorectal cancer treatments include chemotherapy drugs, e.g., 5-fluorouracil, capecitabine, irinotecan, and oxaliplatin together with bevacizumab, an anti-angiogenic agent targeting the vascular endothelial growth factor (VEGF), and cetuximab which targets the endothelial growth factor (EGF). However, resistance reduces the effectiveness of many of these drugs [15]. Regorafenib, an orally active tyrosine kinase inhibitor, shows anti-angiogenic activity due to its dual targeted VEGFR2-TIE2 tyrosine kinase inhibition and is indicated for metastatic colorectal cancer (mCRC) [16]. New therapeutic approaches in the treatment of CRC have emerged such as immunotherapy with limited success, while microtubule inhibitors such as taxanes are not generally effective in CRC [17]. The development of new treatments for CRC is therefore required to enable a continuous improvement of patient outcomes.

Small molecule compounds which interact with microtubules are recognised as one of the major clinical options for cancer treatment [18], while targeted drugs and immunotherapies have shown significant potential in the development of effective cancer therapeutics [19]. Tubulin plays an essential role in many cellular activities and is the target of a number of important chemotherapeutic drugs, e.g., taxol, vincristine, and vinblastine, which destabilise tubulin [20,21], while colchicine (**1**) inhibits the polymerisation of tubulin and acts at the α,β-intrasubunit interface of the tubulin dimer [22]. Tubulin polymerisation is one of the most attractive and promising targets for the discovery of novel anticancer agents [23] and many structurally diverse compounds have been identified as colchicine-binding site ligands [24]. However, many limitations which are related to poor selectivity and high toxicity still exist with conventional chemotherapies.

**Figure 1:**
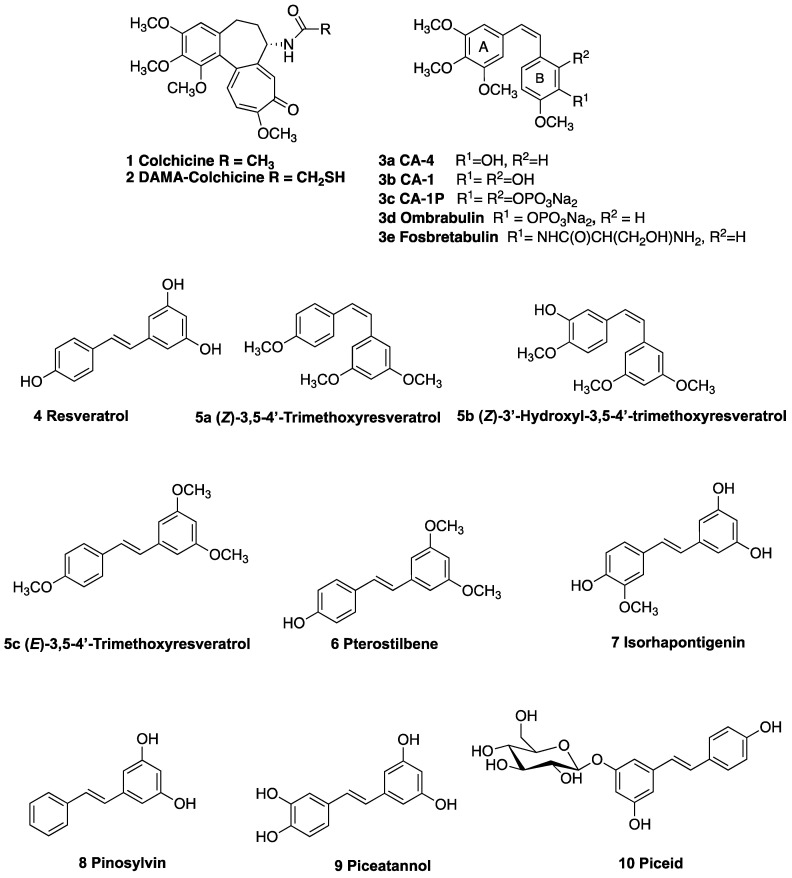
Colchicine (**1**), DAMA-colchicine (**2**), combretastatins CA-1, CA-1P, and CA-4, ombrabulin, fosbretabulin (**3a**–**e**), resveratrol (**4**), and related stilbenes (**5**–**10**).

Stilbene-based compounds are widely occurring natural products and demonstrate a range of biological activities [25,26]. Combretastatins are a group of *cis*-stilbenes isolated from the South African tree *Combretum caffrum* [27]. Combretastatin CA-4 (**3a**), CA-1 (**3b**), and related phosphate and amino acid prodrugs (**3c**–**e**) (Figure 1) show potent anticancer activity in many human cancer cells together with the inhibition of tubulin polymerisation and anti-vascular effects [28,29]. The introduction of a heterocyclic ring to replace the olefinic bond of CA-4 prevents *cis*–*trans* isomerisation, while retaining antimitotic activity [30,31]. We previously reported the potent antiproliferative and antimitotic effects of the *β*-lactam-containing analogues of CA-4 [32,33,34], while related investigations into the antiproliferative and anti-tubulin activity of azetidin-2-ones have also been reported [35,36,37].

The role of the 3,4,5-trimethoxy-substituted A-Ring of CA-4 in inhibiting tubulin polymerisation is demonstrated in the X-ray crystallographic studies of CA-4, DAMA-colchicine (**2**) [22] and the related colchicine-binding site ligands co-crystallised in tubulin [38]. The 3,4,5-trimethoxyphenyl Ring A of CA-4 (**3a**) occupies a hydrophobic pocket at the colchicine-binding site of tubulin [22,35,38], as demonstrated in the X-ray crystal structures of CA-4 (**3a**) [38] and other colchicine-binding site inhibitors (CBSIs) [39].

The 3,5-dihydroxyphenyl substitution pattern is characteristic of stilbenes such as resveratrol (**4**), (Figure 1) [40,41,42] with therapeutic and chemopreventive properties in colorectal and skin cancers [43,44,45]. Resveratrol demonstrates antioxidant effects [46], while the methylated resveratrol derivative (*Z*)-3,5,4′-trimethoxystilbene (**5a**) is 100-fold more active than resveratrol (**4**) in human colon cancer Caco-2 cells [42]; (*Z*)-3′-hydroxyl-3,5,4′-trimethoxystilbene (**5b**) is a potent vascular disrupting agent [47]. The *trans*-3,5,4′-trimethoxystilbene (**5c**) also demonstrates antiproliferative effects in human colon cancer cells [48]. The 3,5-dimethoxyphenyl substitution pattern occurs in pterostilbene (**6**), while the 3,5-dihydroxyphenyl substitution is present in isorhapontigenin (**7**), pinosylvin (**8**), piceatannol (**9**) [25,49,50], and related glucoside piceid (**10**) [51], which have anticancer properties [52] (Figure 1).

The three methoxy groups of colchicine (**1**) and Ring A of CA-4 (**3a**) are regarded as required for efficient binding to the colchicine site [53]. The 3,5-Dimethoxy ring A analogues of CA-4 and resveratrol have been reported [54,55], while replacement of one of the ring A methoxy groups by a fluorine atom showed similar activity to CA-4 [56]. 2-Azetidinones with 3,4,5-trimethoxyphenyl or 3,5-dimethoxyphenyl substituents at the N1 position of the *β*-lactam display antitumour activity [36,57,58,59].

**Figure 2:**
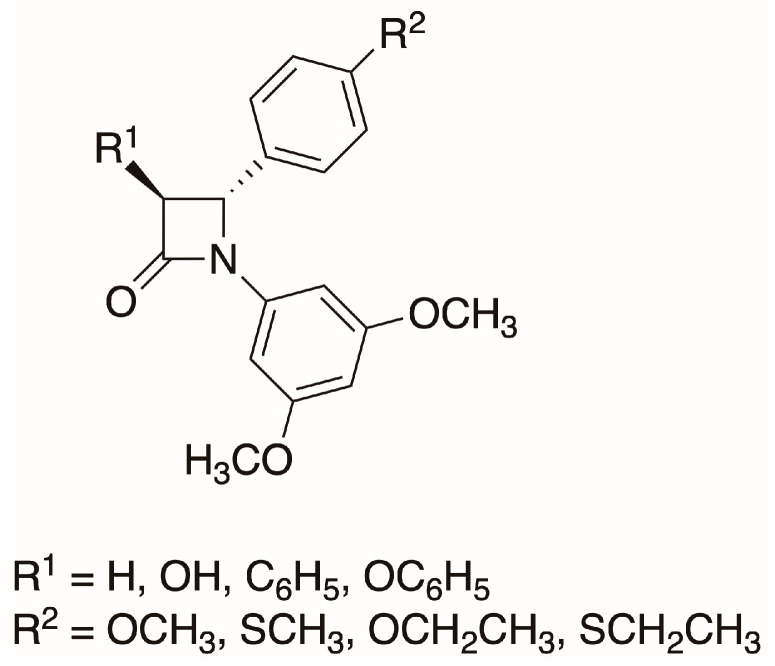
1-(3,5-Dimethoxyphenyl)azetidine-2-ones investigated.

The specific objectives of the present study were as follows: (i) to investigate the synthesis of a panel of 3,5-dimethoxyaryl Ring A *β*-lactam compounds; (ii) to determine the antiproliferative activity of the novel compounds in MCF-7 breast cancer cells and HT-29 chemoresistant colon cancer cells; and (iii) to establish the role of the 3,5-dimethoxyaryl Ring A in the biological activity of these and previously reported compounds using single crystal X-ray analysis, molecular modelling, docking studies, and molecular dynamics (MD) simulation. We focused on the design of a series of 1,4-diaryl-2-azetidinones where the 3,5-dimethoxyphenyl substituent at N-1 replaces the 3,4,5-trimethoxyphenyl Ring A of combretastatin CA-4 as we wished to determine whether the absence of the *para*-methoxy group at N1-aryl Ring A of the *β*-lactam could affect the tubulin–colchicine binding site interactions and potency of these compounds. The target structures are illustrated in Figure 2. Phenyl, hydroxyl, or phenoxy substituents are located at C-3 of the β-lactam ring, together with some 3-unsubstituted examples. At C-4 of the β-lactam (Ring B), we have included 4-methoxyphenyl, 4-ethoxyphenyl, 4-thiomethylphenyl, and 4-thioethylphenyl substituents. Single crystal X-ray analysis, molecular modelling, docking studies, and molecular dynamics (MD) simulation are essential in providing a comprehensive understanding of the structural relationship between the drug molecule, and the receptor protein relevant to a disease. The role of docking in determining molecular recognition and binding between a ligand and a target protein molecule is important in drug design and is widely used in drug discovery, multitarget ligand design, hit identification, and optimization studies. Molecular dynamics (MD) simulation is a key method used for modelling conformational changes within a ligand–target protein complex when binding a small molecule drug. In the present study, single crystal X-ray studies examined the specific structural features of the compounds synthesised while molecular modelling, docking studies, and molecular dynamics studied the role of the novel 3,5-dimethoxyphenyl Ring A interactions with the colchicine–tubulin binding site residues.

## 2. Results and Discussion

### 2.1. Synthesis of β-Lactam Compounds

**Scheme 1:**
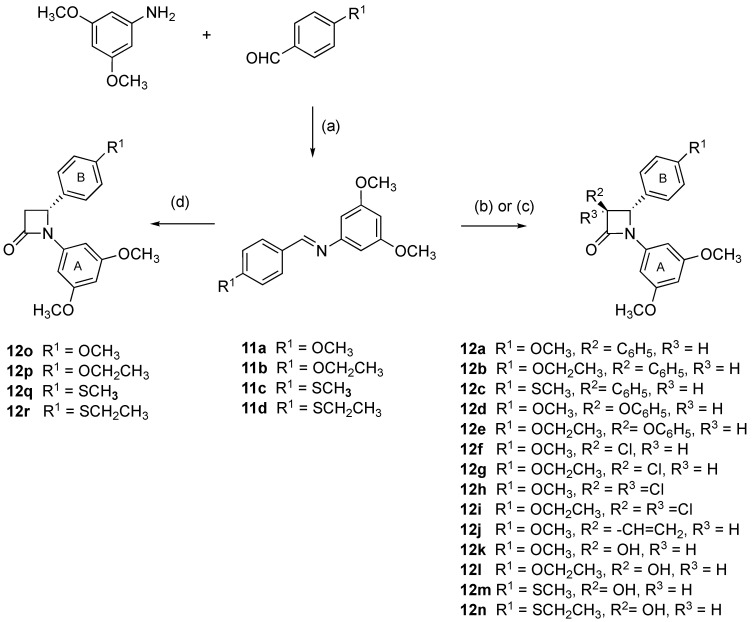
Synthesis of *β*-lactams **12a**–**r**. Reagents and conditions: (a) H_2_O, 30 min, 20 °C (85–97%); (b) compounds **12a**–**12j**: Triethylamine, acid chloride [C_6_H_5_CH_2_COCl, C_6_H_5_OCH_2_COCl, ClCH_2_COCl, Cl_2_CHCOCl, CH_3_CH=CH-COCl, or CH_3_COOCH_2_COCl], toluene, reflux, N_2_, 5 h, (11–31%); **12f**–**i** [61], **12j** [33], and **12k** [36]; (c) compounds **12k**–**12n**: (i) CH_3_COOCH_2_COCl, toluene, reflux, N_2_, 5 h, (11–31%), and (ii) NH_2_NH_2_·2HCl, Triethylamine, MeOH, reflux, 4 h.; and (d) compounds **12o**–**12r**: Ethyl bromoacetate, Zn dust, TMSCl, 40 °C, 15 min, then 100 °C, 2 min, microwave, C_6_H_6_, 100 °C, 30 min, microwave. Products obtained as racemic mixtures; one enantiomer illustrated.

A panel of 3,4-diaryl-*β*-lactam compounds was synthesized, all containing the 3,5-dimethoxyphenyl substituent (Ring A) at N-1 and an ether (methoxy, ethoxy) or thioether (thiomethyl or thioethyl) substituent at C-4 of the aryl ring B. The substituents phenyl, phenoxy, and hydroxy were positioned at C-3 of the *β*-lactam ring. Some examples of 3-unsubstituted *β*-lactams were also included in this study (Scheme 1). The compounds **12a**–**n** were prepared by reaction of the appropriate acid chlorides (phenylacetyl, phenoxyacetyl and acetoxyacetyl) with a suitable substituted imine using a Staudinger-type reaction. A Reformatsky reaction of the appropriate imine and ethyl bromoacetate was used to prepare the 3-unsubstituted *β*-lactams **12o**–**r**.

The required imines **11a**–**d** were prepared in good yield (85–97%) by condensation of 3,5-dimethoxyaniline with the appropriate benzaldehyde and optimised in aqueous conditions at ambient temperature for 30 min [60]. Compounds **12a**–**12c**, containing an aryl substituent at C-3, were obtained by a Staudinger reaction of phenylacetyl chloride with the appropriate imine to afford the racemic products. This panel was synthesised to evaluate the effect on the activity of the replacement of the 3,4,5-trimethoxyphenyl Ring A with 3,5-dimethoxyphenyl Ring A, and the inclusion of 4-methoxyphenyl, 4-thiomethylphenyl, or 4-thioethylphenyl moiety at the N-1 position (Ring B). The racemic products **12a**–**12c** were obtained with exclusively *trans* stereochemistry observed for the *β*-lactam C-3 and C-4 ring substituents. The ^1^H NMR spectrum of **12c** shows H-3 and H-4 as coupled doublet signals at δ 4.21 (H-3) and δ 4.85 (H-4), *J* = 2.49 Hz, while C-3, C-4, and C-2 of the *β*-lactam ring are identified at 63.7, 65.1, and 161.1 ppm, respectively, in the ^13^C NMR spectrum.

The 3-phenoxy *β*-lactams **12d** and **12e** were similarly prepared by reaction of imines **11a** and **11b** with phenoxyacetyl chloride by a modified Staudinger reaction (Scheme 1). In the ^1^H NMR spectrum of the 3-phenoxy-substituted *β*-lactam **12e**, H-3 and H-4 are observed as coupled doublet signals at δ 5.05 (H-3) and δ 4.90 (H-4), (*J_3,4_ =* 1.22 Hz), indicating the formation of a *trans* isomer. In the ^13^C NMR spectrum of compound **12e**, C-3 and C-4 of the *β*-lactam ring of the β-lactam carbonyl carbon are observed at 63.55 and 64.01 ppm, respectively. For compound **12e**, the minor *cis* stereoisomer was isolated with characteristic coupled doublet signals in the ^1^H NMR spectrum at δ 5.49 (H-3) and 5.28 (H-4), *J_3,4_=* 4.88 Hz.

**Figure 3:**
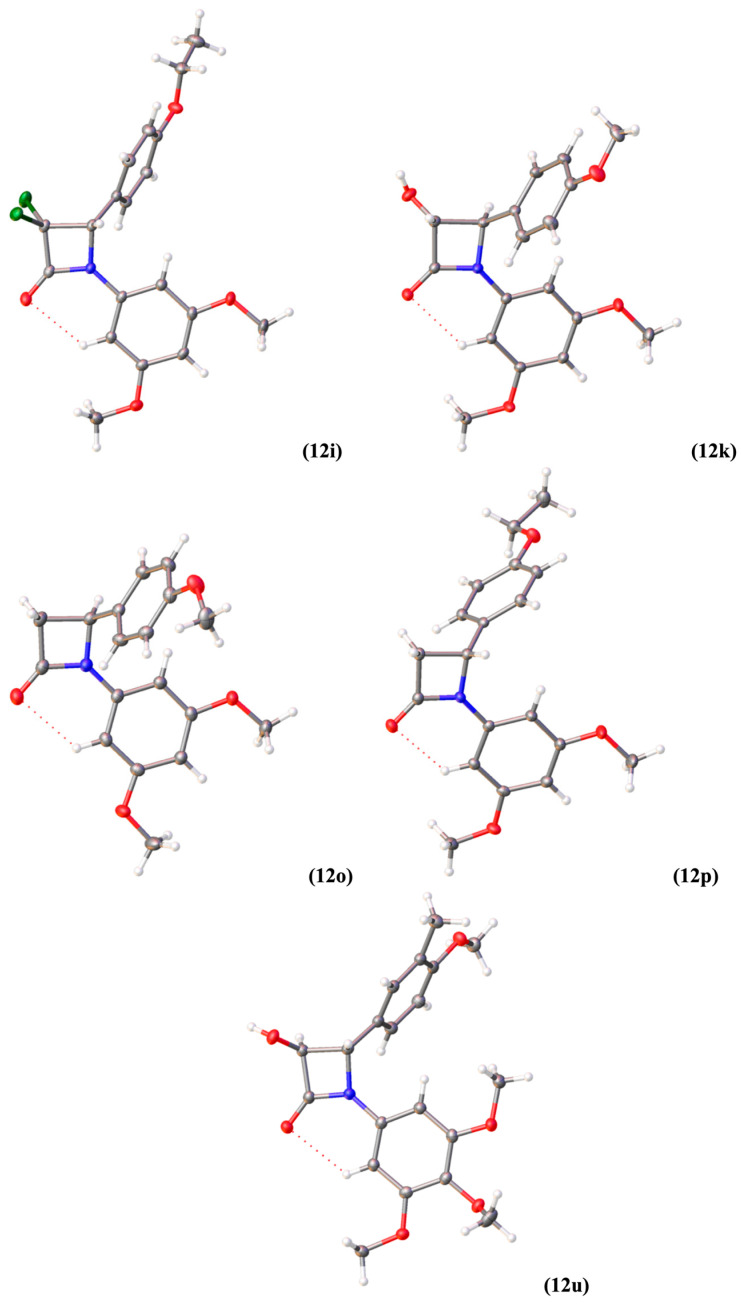
View of the molecular structure of the compounds **12i**, **12k**, **12o**, **12p**, and **12u** with atomic displacement shown at 50% probability. Intramolecular H-bonding is represented by a dotted line. (CCDC depositions 2452665, 2452666, 2452667, 2452668, 2452669, respectively).

The 3-Chloro *β*-lactams **12f** and **12g** and the 3,3-dichloro *β*-lactams **12h** and **12i**, having 4-methoxy and 4-ethoxy substituents at C-4 of the B ring, were prepared in a modified Staudinger reaction as previously reported by us (Scheme 1) [61], while the 3-vinyl *β*-lactam compound **12j** was obtained by a reaction of the appropriate imine **11j** with crotonyl chloride as a *trans* isomer, as previously reported [33]. The 3-hydroxy *β*-lactams **12l**–**12n** were prepared by a Staudinger reaction using acetoxacetyl chloride (Scheme 1). The 3-acetoxy *β*-lactams intermediates were then deprotected with hydrazine dihydrochloride to yield the corresponding 3-hydroxy *β*-lactams at an improved yield when compared with our previous use of potassium carbonate for this reaction [62]. Compound **12k** was prepared as previously reported [36]. In the ^1^H NMR spectrum of the 3-hydroxy *β*-lactam **12l**, H-3 and H-4 are observed as coupled doublet signals (δ 4.68 and 4.77, respectively, *J* = 1.83 Hz), while C-3 and C-4 were identified in the ^13^C NMR spectrum at 60.65 and 66.43 ppm, respectively. The definitive structural confirmation of the proposed configuration for compound **12k** was obtained from an X-ray analysis, which showed a *trans* configuration for the H-3 and H-4 protons (Figure 3).

Microwave technology was successfully applied for the Reformatsky synthesis of 3-unsubstituted *β*-lactams, **12o**–**12r**, using zinc activation by trimethylchlorosilane (TMCS), followed by a reaction with the appropriate imine and ethyl bromoacetate [63] (Scheme 1). The ^1^H NMR spectrum of **12q** displayed geminally coupled diastereotopic H-3 protons at δ 2.86 (dd, *J* = 15.26, 3.05 Hz) and δ 3.49 (dd, *J* = 15.26, 6.10 Hz), and H-3 protons coupled to H-4 (δ 4.89, *J* = 5.80, 2.75 Hz). In the ^13^C NMR spectrum, C-3, C-4, and C-2 are identified at 46.87, 60.86, and 164.65 ppm. All compounds in the panel displayed characteristic *β*-lactam carbonyl absorbance in the IR spectrum in the region ν1774–1723 cm^−1^. The *β*-lactams containing the 3,4,5-trimethoxy-substituted Ring A (**12s** [36,64], **12t** [64], and **12u** [32] (Figure 3) were prepared as we previously described, and used as control compounds for a comparison of antiproliferative activities with the panel of novel 3,5-dimethoxy-substituted Ring A compounds designed in the present study. Confirmation of the proposed *trans* configuration for the H-3 and H-4 protons of compound **12u** was obtained from an X-ray analysis (Figure 3).

### 2.2. Single Crystal X-Ray Crystallography Study of Azetidine-2-ones **12i**, **12k**, **12o**, **12p** and **12u**

**Figure 4:**
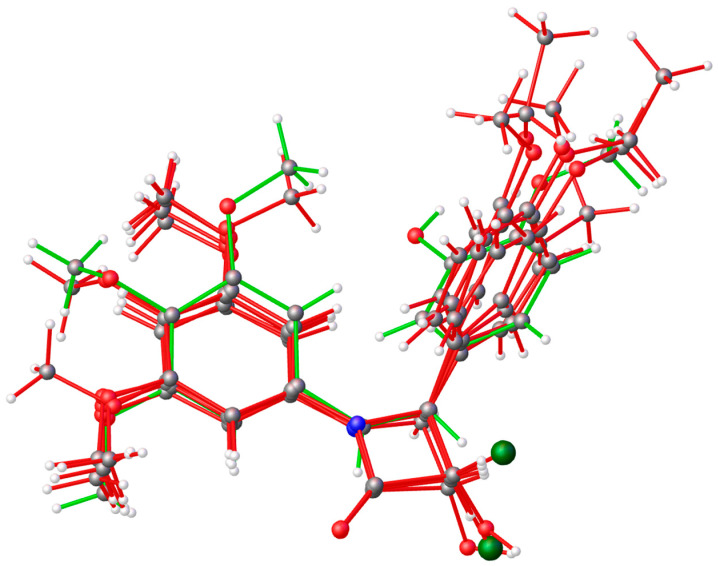
A view of the overlay of the same chirality of **12i**, **12k**, **12o**, **12p**, **12u** (CCDC depositions 2452665, 2452666, 2452667, 2452668, 2452669), and CA-4 (CCDC 646589, green bonds). Where possible, the nitrogen and ketone oxygen atoms have been used to anchor the overlap. For CA-4, *ipso* phenyl carbon and vinyl carbon (C3 and C1) atoms were used as anchors.

X-ray crystallography is useful in the development of novel pharmaceutical compounds, as it confirms molecular configuration, and shows hydrogen bonding and other interactions which can influence stability and the biological activity of molecules. The structures of the azetidine-2-ones **12i**, **12k**, **12o**, and **12p** (with 3,5-dimethoxyphenyl Ring A), and the related azetidine-2-one **12u** (with 3,4,5-dimethoxy Ring A) were determined by single crystal X-ray analysis, and the crystal structures are displayed in Figure 3. The crystal data and structure refinement for these compounds are displayed in Table S1 and Figure S1, Supplementary Information. The *trans* stereochemistry of the *β*-lactam ring protons at C-3 and C-4 is confirmed for compounds **12k** and **12u;** this result supports the initial structural assignments based on ^1^H NMR data, and is in agreement with the X-ray data previously reported for combretastatins [65,66] and related monocyclic *β*-lactams [67]. A view of the overlay of compounds **12i**, **12k**, **12o**, **12p**, **12u**, and CA-4 is displayed in Figure 4. The 3,5-dimethoxy Ring compounds **12i**, **12k**, **12o**, and **12p** together with the 3,4,5-trimethoxy Ring A compound **12u** demonstrate a similar conformationally restricted *β*-lactam scaffold for the planar aryl rings A and B, indicating that the absence of the 4-methoxy group in compounds **12i**, **12k**, **12o**, and **12p** does not cause a change in conformation for the compounds, compared with **12u** or **CA-4**. The 3,4,5-trimethoxy Ring A arrangement is usually observed for the characteristic interactions required for the colchicine-binding site of tubulin. This study also shows hydrogen bonding between the carbonyl oxygen of the *β*-lactam ring and the hydrogen at the *ortho* position of ring A, and this is observed for each of the compounds (Figure 3). It is evident that rings A and B are not coplanar (Table 1); a rigid configuration is demonstrated with torsional angles for ring A/B observed for compounds **12i**, **12k**, **12o**, **12p**, and **12u** of 63.36°, −62.89°, −57.13°, 62.53°, and 60.46°, respectively; whereas the ring A/B comparable value for DAMA-colchicine is 53° [22,65] and for CA-4 is −9.4° [66].

The structural parameters for **12i**, **12k**, **12o**, **12p**, and **12u** are given in Table 2. The carbonyl bond lengths for the *β*-lactams were found in the range 1.202–1.222 Å, which are consistent with previously reported bond lengths for carbonyl bonds in monocyclic *β*-lactams, e.g., 1.217(3) Å [68] and 1.207(2) Å [69]. The *β*-lactam C-2/C-3 bond lengths, 1.541–1.526 Å, are within the ranges reported of 1.52–1.55 Å, while the N1/C-4 bond lengths of 1.485–1.495 Å were in the expected range of 1.49–1.51 Å [67,70]. The amide C-2/N1 bond lengths for compounds **12i**, **12k**, **12o**, **12p**, and **12u** were observed in the range 1.358–1.377 Å. Due to the strained *β*-lactam ring present in these compounds, a longer C-2/N1 amide bond length (1.36–1.38 Å) is observed when compared with the normal amide bond length of 1.33 Å; additionally, the C=O bond length is shorter (1.20–1.22 Å) when compared with the reported amide bond length (1.24 Å) [70], also due to decreased amide resonance [71]. A view of the overlay of compounds **12i**, **12k**, **12o**, and **12p** (having 3,5-dimethoxyphenyl Ring A) is displayed in Figure 4, and demonstrates that these molecules align very closely with CA-4; little difference is observed in the conformation of these compounds by the absence of the 4-methoxy substituent on Ring A when compared with the compound **12u**, having the 3,4,5-trimethoxyphenyl Ring A.

Hirshfeld surface analysis was used to visualize and quantify the intermolecular interactions in the crystal structure for the *β*-lactam molecules **12i**–**12o**. The Hirshfeld surfaces have been calculated and fingerprint plots are shown in the Supplementary Information (Figures S50–S54), which demonstrate the intermolecular contacts in the crystal structures of compounds **12i**–**12o**. The compound **12i** shows predominantly weak C-H∙∙∙O (lactam-CH to methoxide) and Cl∙∙∙O interactions in the crystal structure (Figure S50); the compound **12k** shows predominant OH∙∙∙O interactions, (Figure S51), while **12o** shows predominant weak CH∙∙∙O (aryl ring—methoxide) interactions, (Figure S52). Weak CH∙∙∙O interactions (methoxide-CH_3_∙∙∙methoxide, ethoxide-CH_3_∙∙∙methoxide; lactam-CH_2_∙∙∙ketone) are observed in the crystal structure for **12p** (Figure S53), while OH∙∙∙O interactions (lactam OH ∙∙∙ketone O) are predominant for **12u** (Figure S54).

### 2.3. Antiproliferative Evaluation of Azetidine-2-ones **12a**–**12e**, **12l**–**12r** with 3,5-Dimethoxyphenyl Ring A in MCF-7 Human Breast Cancer Cells

The antiproliferative effects of the *β*-lactam compounds **12a***–***12r** were investigated, in which the 3,5-dimethoxyphenyl ring A replaced the characteristic 3,4,5-trimethoxyphenyl ring A of CA-4. In the present work, the 1-(3,5-dimethoxyphenyl)azetidin-2-one compounds **12a***–***12e**, **12l***–***12r** are novel and hitherto unreported, while the in vitro antiproliferative activities and proapoptotic effects for **12k** were reported by Tripodi et al. [36]. In our study, the C-3 substituents on the *β*-lactam ring were structurally varied and included phenyl, hydroxyl, and phenoxy together with C3-unsubstituted examples. The products were evaluated for antiproliferative activity in MCF-7 human breast cancer cells and also in chemoresistant HT-29 colon cancer cells using the alamarBlue cell viability assay. The results obtained are displayed in Table 3 and Table 4. From our previous research on related 3,4,5-trimethoxyphenylazetidin-2-ones, the introduction of an aryl, phenoxy, methyl, vinyl, hydroxyl, and chloro substituents at the C-3 position of the azetidine-2-one ring resulted in compounds with excellent antiproliferative properties [32,34,61,63].

**Figure 5:**
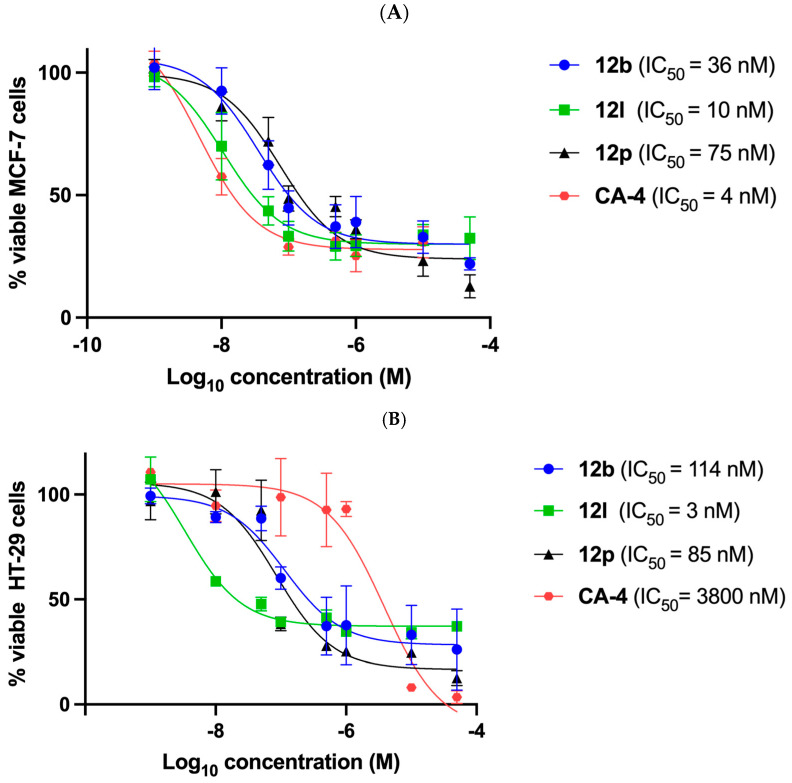
Antiproliferative activity of *β*-lactams **12b**, **12l**, and **12p** in MCF-7 breast cancer cells and HT-29 chemoresistant colon cancer cells. (**A**) Antiproliferative activity for *β*-lactams **12b**, **12l**, **12p**, and CA-4 in MCF-7 cells and (**B**) antiproliferative activity for *β*-lactams **12b**, **12l**, **12p**, and CA-4 in HT-29 cells. Cells were grown in 96-well plates and treated with the *β*-lactam compounds **12b**, **12l**, **12p**, and CA-4 at 1 nM–50 μM for 72 h. Cell viability was measured by AlamarBlue assay (average of three independent experiments) expressed as a percentage of vehicle control (ethanol 1% (*v*/*v*))-treated cells. The values represent the mean ± SEM for three independent experiments performed in triplicate.

As shown in Table 3, all the 3,5-dimethoxy ring A *β*-lactam compounds with 4-methoxyphenyl ring B demonstrated sub-micromolar activity in MCF-7 cells. The control compound CA-4 was also screened in MCF-7 breast cancer cells and demonstrated an IC_50_ value of 4.6 nM. The most potent compounds in the *p*-methoxyphenyl series that exhibited antiproliferative activity in the nanomolar range were compounds **12a**, **12d**, **12o**, and **12k**, with IC_50_ values in MCF-7 cells of 25, 54, 55, and 1.5 nM, respectively. In the *p*-ethoxyphenyl series of compounds, 3-hydroxy analogue **12l** was the most potent example in the series, with an IC_50_ value for compound **12l** of 10 nM in MCF-7 cells; the IC_50_ values for 3-phenyl **12b** and the 3-phenoxy analogue **12e** were 36 nM and 273 nM, respectively, in MCF-7 cells. All *β*-lactam compounds bearing *p*-thiomethylphenyl Ring B, 3-phenyl **12c**, 3-unsubstituted **12q**, and 3-hydroxy **12m**, exhibited effects in the sub-micromolar IC_50_ range of 23–244 nM in MCF-7 cell, with the 3-hydroxyl compound **12m** identified as the most potent (IC_50_ = 23 nM). The replacement of the *para*-thiomethyl group in compound **12m** by a *para*-thioethyl group to provide the **12n** analogue, resulted in a slight reduction in activity, with IC_50_ values of 23 nM and 31 nM, respectively, in MCF-7 cells. In contrast, a much more noticeable decrease in activity was observed upon comparison of the activities of the 3-unsubstituted *para*-thiomethylphenyl compounds **12q** and **12r** (IC_50_ = 150 nM and 11360 nM, respectively). The cell viability curves for the *p*-ethoxyphenyl series of *β*-lactams **12b**, **12l**, and **12p**, together with the control CA-4, in MCF-7 cells are illustrated in Figure 5A and demonstrate their relative potencies. The IC_50_ values for the 3-chloro, 3,3-dichloro, and 3-vinyl *β*-lactams **12f***–***12j** (3,5-dimethoxy ring A) were 680, 945, 6612, 1112, and 170 nM, respectively, in MCF-7 cells, as we previously reported [33,61] and are included for comparison, indicating that in the present study the introduction of hydroxyl, phenyl, or phenoxy at C-3, together with C3-unsubstituted examples generally resulted in an improvement in activity (Table 3).

Compound **12l** displayed the most potent antiproliferative effect in the series of compounds in the MCF-7 cells, with an IC_50_ value of 10 nM. This is consistent with our previous finding that the 3-hydroxy compounds were much more active than other substituents at this position. It is interesting that the 4-thiomethylphenyl-3-hydroxy *β*-lactam **12m** displayed a 2.5-fold decrease in antiproliferative effects when compared with the corresponding 4-ethoxyphenyl compound **12l**. Other substituents which also resulted in potent activities were 3-phenoxy **12a**, 3-phenyl **12b**, and 3-unsubstituted **12o** analogues. These observations could infer that the 3,5-dimethoxyphenyl Ring A was favourable for interaction of the molecule with the colchicine-binding site of tubulin, and the *para*-methoxyphenyl group of Ring A may not be as critical for this interaction.

An evaluation of selected compounds in chemoresistant HT-29 colon cancer cells demonstrated that the 3,5-dimethoxy ring A *β*-lactam compounds with 4-methoxyphenyl ring B **12k** (3-hydroxy) and **12o** (3-unsubstituted) showed sub-micromolar activity, with IC_50_ values of 12 nM and 89 nM, respectively (Table 4). The control compound CA-4 was also screened in CA-4 resistant HT-29 colorectal cancer cells, and demonstrated an IC_50_ value of 3810 nM, as CA-4 is rapidly metabolised in HT-29 cells by glucuronidation with UDP-glucuronosyltransferases (UGTs) [64,72]. Compound **12l** (3,5-dimethoxy ring A *β*-lactam compound with 4-ethoxyphenyl ring B) displayed the most potent antiproliferative effect in the HT-29 cell line, with an IC_50_ value of 3 nM, compared to 3810 nM for CA-4. In contrast, 3-hydroxy *β*-lactams with 4-methoxyphenyl **12k** and 4-thiomethylphenyl **12m** displayed a decrease in antiproliferative effects (IC_50_ = 12 nM and 26 nM, respectively) when compared with **12l** (IC_50_ = 3 nM). This is consistent with our previous finding that the 3-hydroxy compounds showed greater antiproliferative effects when compared with 1,4-diarylazetidinones, having alternative substituents at this position. In the *p*-ethoxyphenyl series of compounds, 3-hydroxy **12l**, 3-unsubstituted **12p**, and 3-phenyl analogue **12b** were the most potent in the series, with IC_50_ values 3 nM, 85 nM, and 114 nM, respectively, in HT-29 cells. The 3-hydroxy *β*-lactam compound **12m**, bearing the *p*-thiomethylphenyl Ring B, demonstrated potent activity with IC_50_ = 26 nM, while the 3-phenyl **12c** and 3-unsubstituted **12q** exhibited sub-micromolar IC_50_ values in HT-29 cells of 115 nM and 747 nM, respectively. These findings agree with the reported data, suggesting that the deletion of the CA-4 B ring *meta* hydroxyl substituent from *β*-lactam is advantageous for increasing biological activity in HT-29 colorectal cells [32,64]. The relative potency between MCF-7 and HT-29 cells for **12m** of 0.885 demonstrates comparable activity in both cell lines, while the relative potency for **12l** is 3.088, indicating greater potency in MCF-7 cells. The cell viability curves for the *p*-ethoxyphenyl series of *β*-lactams compounds **12b**, **12l**, and **12p**, together with the control CA-4, in HT-29 cells are illustrated in Figure 5B. While the potency of **12l** is comparable to CA-4 in MCF-7 cells, its relative potency is over 1000 times greater than CA-4 in chemoresistant HT-29 colon cancer cells, which supports our previously reported results indicating that the replacement or deletion of the B ring *meta* hydroxyl of CA-4 and analogues prevents inactivation at the B ring *meta* hydroxyl position by glucuronidation [32,64].

In the series of compounds investigated, compound **12l** displayed a potent antiproliferative effect in both the MCF-7 and HT-29 cell lines, with IC_50_ values of 10 nM and 3 nM, respectively. The control compound CA-4 demonstrated notably poorer antiproliferative activity in HT-29 cells (IC_50_ = 3814 nM, Table 4) when compared to its excellent activity in breast cancer cells (IC_50_ = 4.6 nM, Table 3). Antiproliferative data for *β*-lactam **12t** (Ring A: 3,4,5-trimethoxy, Ring B: 4-ethoxy) were included for comparison (IC_50_ values of 7 nM (MCF-7) and 15 nM (HT-29)) as we previously reported [64]. The 3,5-dimethoxyphenyl **12l** shows superior activity in both cell lines when compared with **12t**. The IC_50_ data for betalactams **12s** [36,64] and **12u** [32] (both containing the 3,4,5-trimethoxy Ring A) were also included in this study for comparison of activity and an analysis of the previously unreported X-ray structural features for compound **12u** (See Figure 3).

We also investigated a potential relationship between physicochemical properties and cytotoxicity for the series of synthesised compounds. We did not observe a correlation between the molecular volume of the compounds and biological activity (see Supplementary Information, Table S3). For compound **12l** (logP = 2.40), the slightly bulkier and more lipophilic ethyl substituent resulted in comparable anticancer activity (IC_50_ = 10 nM for MCF-7 and 3 nM for HT-29) when compared with the methoxy analogue **12k** (logP =2.02, IC_50_ = 1.5 nM for MCF-7 and 12 nM for HT-29). In HT-29 cells, the ethoxy compound **12p** (logP = 3.11, IC_50_ = 8 nM) displays similar potency to the methoxy compound **12o** (logP 2.75, IC_50_ = 9 nM). These results may support the hypothesis that the aryl ring B of our compounds interacts with a hydrophobic pocket in the colchicine–tubulin binding site. Compounds **12l**, **12m**, **12n**, and **12o** with IC_50_ values in the range 3–55 nM in MCF-7 cells and logP values in the range 2.40–2.94 indicate that a lower logP value was beneficial for cancer cell growth inhibition. In contrast, compounds **12a**, **12b**, and **12d**, which were also potent inhibitors of cell growth, with IC_50_ values of 25, 36, and 54 nM display higher logP values of 3.97, 4.29, and 3.69, respectively. However, a direct correlation between the lipophilicity (logP) and the antiproliferative activity of the compounds in MCF-7 cells was not evident across the series and logP may not be useful in predicting relative activity in the series.

**Table 3:** Antiproliferative activity of *β*-lactam compounds containing 3,5-dimethoxyphenyl Ring A in MCF-7 human breast cancer cells.

Compound	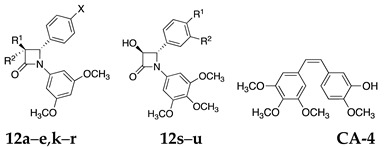	MCF-7 IC_50_ (nM) ^a^	LogP ^e^
12a	R^1^ = C_6_H_5_, R^2^ = H, X = OCH_3_	25 ± 3	3.97
12b	R^1^ = C_6_H_5_, R^2^ = H, X = OCH_2_CH_3_	36 ± 10	4.29
12c	R^1^ = C_6_H_5_, R^2^ = H, X = SCH_3_	244 ± 60	4.51
12d	R^1^ = OC_6_H_5_, R^2^ = H, X = OCH_3_	54 ± 10	3.69
12e	R^1^ = OC_6_H_5_, R^2^ = H, X = OCH_2_CH_3_	273 ± 10	4.02
12k ^d^	R^1^ = OH, R^2^ = H, X = OCH_3_.	1.5 ± 0.7	2.02
12l	R^1^ = OH, R^2^ = H, X = OCH_2_CH_3_	10.5 ± 0.9	2.40
12m	R^1^ = OH, R^2^ = H, X = SCH_3_	23 ± 2	2.54
12n	R^1^ = OH, R^2^ = H, X = SCH_2_CH_3_	31 ± 6	2.94
12o	R^1^ = H, R^2^ = H, X = OCH_3_	55 ± 10	2.75
12p	R^1^ = H, R^2^ = H, X = OCH_2_CH_3_	75 ± 6	3.11
12q	R^1^ = H, R^2^ = H, X = SCH_3_	150 ± 40	3.27
12r	R^1^ = H, R^2^ = H, X = SCH_2_CH_3_	11,360 ± 800	3.60
12s ^b^	R^1^ = OCH_3_, R^2^ = H	4 ± 0.4	2.02
12t ^d^	R^1^ = OCH_2_CH_3_, R^2^ = H	7 ± 0.9	2.28
12u ^c^	R^1^ = OCH_3_, R^2^ = CH_3_	5 ± 1	2.31
CA-4 ^f^		4.6 ±0.32	3.27

^a^ The IC_50_ value is the half-maximal inhibitory concentration required to inhibit the growth of MCF-7 breast cancer cells. Values represent the mean ± SEM (error values × 10^−6^) for at least three experiments performed in triplicate. Treatment at eight different concentrations in the range 1 nM–50 μM over 72 h was used to determine IC_50_ values in comparison to the control compound CA-4: ^b^ [36], ^c^ [32], and ^d^ [64]. ^e^ The consensus logP o/w is the arithmetic mean of the values predicted by the five proposed methods of the Swiss ADME cheminformatics webtool [73]. ^f^ The IC_50_ value determined for CA-4 (4.6 nM) in MCF-7 cells is in agreement with reported values [74,75]. The IC_50_ values for 3-chloro, 3,3-dichloro, and 3-vinyl *β*-lactams **12f**–**12j** were 680, 945, 6612, 1112, and 170 nM, respectively, in MCF-7 cells as we previously reported [33,61].

**Table 4:** Antiproliferative activity of *β*-lactam compounds containing 3,5-dimethoxyphenyl Ring A in HT-29 colon cancer cells.

Compound	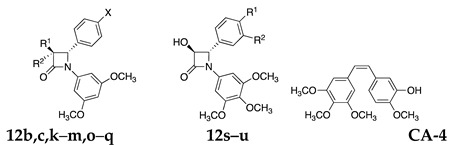	HT-29 IC_50_ (nM) ^a^	Relative Potency ^b^ MCF-7/HT-29
12b	R^1^ = C_6_H_5_, R^2^ = H, X = OCH_2_CH_3_	114 ± 10	0.316
12c	R^1^ = C_6_H_5_, R^2^ = H, X = SCH_3_	115 ± 20	2.122
12k	R^1^ = OH, R^2^ = H, X = OCH_3_	12 ± 3.0	0.167
12l	R^1^ = OH, R^2^ = H, X = OCH_2_CH_3_	3 ± 0.9	3.088
12m	R^1^ = OH, R^2^ = H, X = SCH_3_	26 ± 4.0	0.885
12o	R^1^ = H, R^2^ = H, X = OCH_3_	89 ± 10	0.618
12p	R^1^ = H, R^2^ = H, X = OCH_2_CH_3_	85± 20	0.873
12q	R^1^ = H, R^2^ = H, X = SCH_3_	747 ± 0.0 ^c^	0.200
12s	R^1^ = OCH_3_, R^2^ = H	12 ± 2.0	0.333
12t ^d^	R^1^ = OCH_2_CH_3_, R^2^ = H	15 ± 1	0.466
12u ^e^	R^1^ = OCH_3_, R^2^ = CH_3_	7 ± 2	0.714
CA-4 ^f^		3814 ± 100	0.0012

^a^ IC_50_ value is the half-maximal inhibitory concentration required to inhibit the growth of HT-29 chemoresistant colon cancer cells. Values represent the mean ± SEM (error values × 10^−6^) for at least three experiments performed in triplicate. Cells were treated in the range 1 nM–50 μM at eight different concentrations over 72 h to determine IC_50_ values in comparison to the control compound CA-4. ^b^ Relative potency = IC_50_ in MCF-7 cells/IC_50_ in HT-29 cells. ^c^ Single experiment; ^d^ [64], and ^e^ [32]. ^f^ The IC_50_ value determined for CA-4 in HT-29 cells (3814 nM) is in agreement with the reported values [57,74,75].

### 2.4. Evaluation of In Vitro Cytotoxicity of β-Lactam Compounds

**Figure 6:**
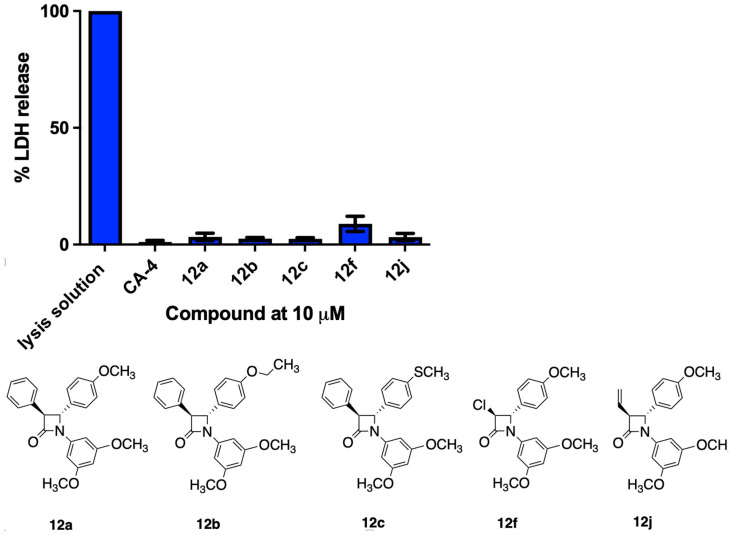
LDH assay for 3,5-dimethoxyphenyl ring A *β*-lactams **12a**, **12b**, **12c**, **12f**, **12j**, and CA-4 in MCF-7 breast cancer cells. Following treatment of the MCF-7 cells at 10 µM for 24 h, LDH release was determined with a Promega LDH assay kit (G1780) [76] and % cell death at 10 µM is shown (see Table S2, **12j** [33]).

In view of the very promising antiproliferative activity results obtained with the MCF-7 and HT-29 cancer cell lines, the in vitro cytotoxicity of the novel *β*-lactam compounds was evaluated in MCF-7 cells using the CytoTox 96^®^ Non-Radioactive Cytotoxicity Assay [76]. The LDH assay is specific for cell death as it measures the concentration of cytosolic protein lactate dehydrogenase (LDH) released during the mechanisms of cell death associated with the loss of cell membrane integrity, apoptosis, or necrosis [77]. MCF-7 cells were treated with the selected compounds **12a**, **12b**, **12c**, and CA-4 at a 10 μM concentration for a 24 h period and the results are presented as a percentage of total LDH release (Figure 6). The compounds selected in this study were among the most potent in antiproliferative activity. The related 3-chloro-3,5-dimethoxyphenyl *β*-lactam **12f**, and 3-vinyl-*β*-lactam compound **12j** [33] were also included in this study. The majority of the compounds demonstrated minimal cytotoxicity (<3.5% at 10 μM concentration), apart from compound **12f** (8.9%). The lowest % LDH released was obtained by the potent 3-phenyl compounds **12b** and **12c**, having the 4-ethoxyphenyl (2.5%) and 4-thiomethylphenyl (2.4%) Ring B as well as the control drug CA-4 10 μM (1.1%) (Figure 6), indicating that these 3,5-dimethoxyphenyl *β*-lactam compounds could be suitable candidates for further investigation as potential antiproliferative agents for ER-positive breast cancers. A cytotoxicity evaluation of the compounds in the HT-29 colon cancer cell line was outside the scope of the present study; however, a cytotoxicity evaluation is required for further progression of the compounds as colon cancer therapeutics.

### 2.5. Predicted Physicochemical Properties, Cheminformatics, and In Vitro ADME Properties for β-Lactams ***12a–u***

The physicochemical and pharmacokinetic properties of active molecules are determined in the early stages of their design to optimise the design of a successful drug candidate for clinical development. The predicted physicochemical characteristics, drug-like features, and pharmacokinetic properties of the selected 3,4-diaryl-2-azetidinone compounds **12a***–***u** were determined using the SwissADME cheminformatics webtool [73] (Supplementary Information, Figures S1 and S2 and Tables S3–S8) together with the Molinspiration Cheminformatics webtool [78]. Some correlations between the biological activity and the predicted physicochemical properties of the compounds were observed. The predicted physicochemical properties of the compounds were found to comply with the requirements of the Lipinski rules, Ghose rules, Veber rules, Egan rules, and Muegge rules. There has been increased interest in the discovery and development of “beyond rule of 5” drugs [79,80]. The descriptors, which are useful to assess drug-likeness, include molecular weight, logP, the number of HB acceptors and donors (HBAs and HBDs), or the number of rotatable bonds (RotBs), and the results obtained for this set of compounds are shown in Table S3. All synthesised compounds have a molecular weight (MW) <500 Da, with molecular weights in the range 313–404 Da. The hydrogen bond acceptors were in the range 3–6, the hydrogen bond donor range was 0–1, there were 5–8 rotatable bonds, and logP was in the range 2.02–4.51 for all compounds. The predicted consensus logPo/w values for all synthesised compounds are reported in Table S4, which confirms that all the compounds have a calculated logP value < 5 and are in the range 2.02–4.51 [81].

Topological Polar Surface Area (TPSA) is calculated for a drug to provide an indication of the drug’s polarity and thus a prediction of its lipid solubility. The TPSA value is a widely used molecular descriptor in the study of drug transport properties such as intestinal absorption, and blood–brain barrier (BBB) penetration. The TPSA values calculated for all the synthesised molecules were <140 Å^2^ and are in the range 48.00–84.30 Å^2^, which is the limit required for cell membrane permeation [82], as shown in Table S3 [83]. Interestingly, all compounds have TPSA < 84.3 Å^2^, with many of the potent compounds, e.g., **12a**, **12b**, **12o**, and **12p**, having TPSA values < 50 Å^2^. Using these descriptors, all the synthesised compounds are predicted to exhibit drug-like properties.

In preclinical investigations, screening for solubility is a critical challenge encountered in the development of new, poorly soluble drug candidates. Using the SwissADME web tool, the aqueous solubility (log *S*) of the compounds can be estimated based on predictive methods implemented in three models ESOL, Ali, and FILTER-IT [73]. Our compounds were classified as “soluble” and “moderately soluble” for a qualitative estimation of aqueous solubility (see Table S5), with a predicted solubility for compound **12o** of 0.134 mg/mL (ESOL) and 0.26 mg/mL (Ali). For a preclinical evaluation of these compounds, their solubility in EtOH or in EtOH/DMSO (10 μg/mL) was used. Optimisation of water-solubility is required for future evaluation of these compounds. The pK_a_H values for compounds **12l**, **12m**, and **12a** were calculated with Chemicalize as 12.3 (OH), 12.29 (OH)), and 9.47 (H-3) and were not predicted to be ionized at physiological pH.

The calculated logP and calculated TPSA of the compounds can be correlated using the Brain Or Intestinal EstimateD permeation method (BOILED-Egg) to display the simultaneous prediction of two key ADME values: gastrointestinal (GI) absorption and brain access (blood–brain barrier, BBB) [73,84] (Supplementary Information, Figures S1 and S2). All the compounds described in our study are predicted to demonstrate high GI absorption, and all compounds except **12m** and **12n** (and our previously reported lead compounds **12u** and **12t**) are predicted to cross the BBB (see Supplementary Table S6 and Figures S1 and S2 for the WLOGP-*versus*-TPSA plot and Bioavailability Radar). The majority of the series of compounds evaluated are predicted not to be substrates of the permeability glycoprotein (P-gp) (except compounds **12b**, **12c**, **12t**, and **12u**) (Supplementary Information Table S6), which is desirable for potential anticancer therapeutics.

Interestingly, most of the compounds in the series followed the Pfizer and GSK rules for drug-likeness (MW ≤ 400, logP ≤ 4), e.g., for the most potent compound **12l**, the MW = 343, predicted logP = 2.40, number of rotatable bonds = 6, number of H-bond acceptors = 5, and number of H-bond donors = 1. Compound **12l** demonstrates a low TPSA value of 68 Å^2^ (TPSA < 75 Å^2^), shows predicted high blood–brain barrier (BBB) absorption together with a high Abbott Bioavailability Score (0.55), and is not predicted to inhibit CYP2D6 metabolic enzyme activity (see Supplementary Information Table S7). Hepatoxicity was not predicted for the majority of the series of compounds evaluated using the pkCSM (http://biosig.unimelb.edu.au/pkcsm/prediction, accessed on 29 August 2025) web interface [85] (Supplementary Information Table S9). All compounds evaluated in the screening panel were confirmed as free from pan-assay interference compound (PAINS) alerts [86] and were identified as suitable candidate compounds for subsequent in vitro biochemical investigations based on their drug-like properties (Tables S3–S8, Supplementary Information). These results indicate that the novel potent compound **12l** is a suitable candidate for further investigation as a potential antiproliferative microtubule-targeting agent for breast and colon cancers.

### 2.6. Molecular Modelling Study of β-Lactams

The computational modelling study was performed on potent compound **12l** as a potential antiproliferative microtubule-targeting agent using the X-ray structure of bovine tubulin co-crystallised with *N*-deacetyl-N-(2-mercaptoacetyl)colchicine (DAMA-colchicine (**2**), CN2), PDB 1Z2B [87], which had a resolution of 4.1 Å. This PDB was selected as the protein chains had a smaller number of breaks than other X-ray structures. Molecular dynamics (MD) simulations were also performed using GROMACS 2020.7 (GNU General Public License http://www.gromacs.org, accessed on 29 August 2025) [88], in order to check the stability of the predicted docked pose. To compare the molecular modelling results of the potent 3,5-dimethoxyphenyl Ring A *β*-lactam compound **12l**, we have also modelled the interactions between the related 3,4,5-trimethoxyphenyl Ring A *β*-lactam compound **12s** [36,64] and the tubulin protein. The 3*S*/4*S* enantiomers of the *trans β*-lactam compounds **12l** and **12s** were selected for the modelling analysis as our previous studies demonstrated that the *S*,*S* enantiomers are more highly ranked than the corresponding *R*,*R* enantiomeric pair.

**Figure 7:**
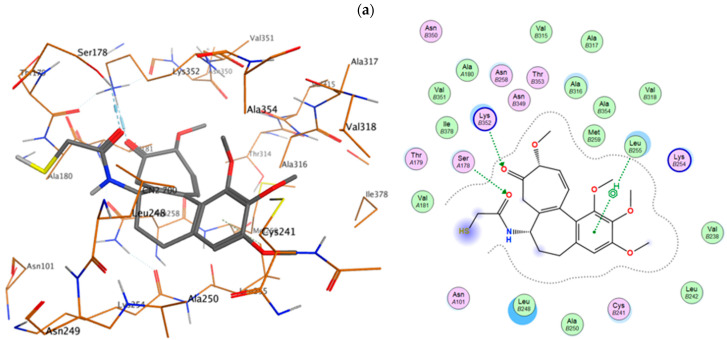

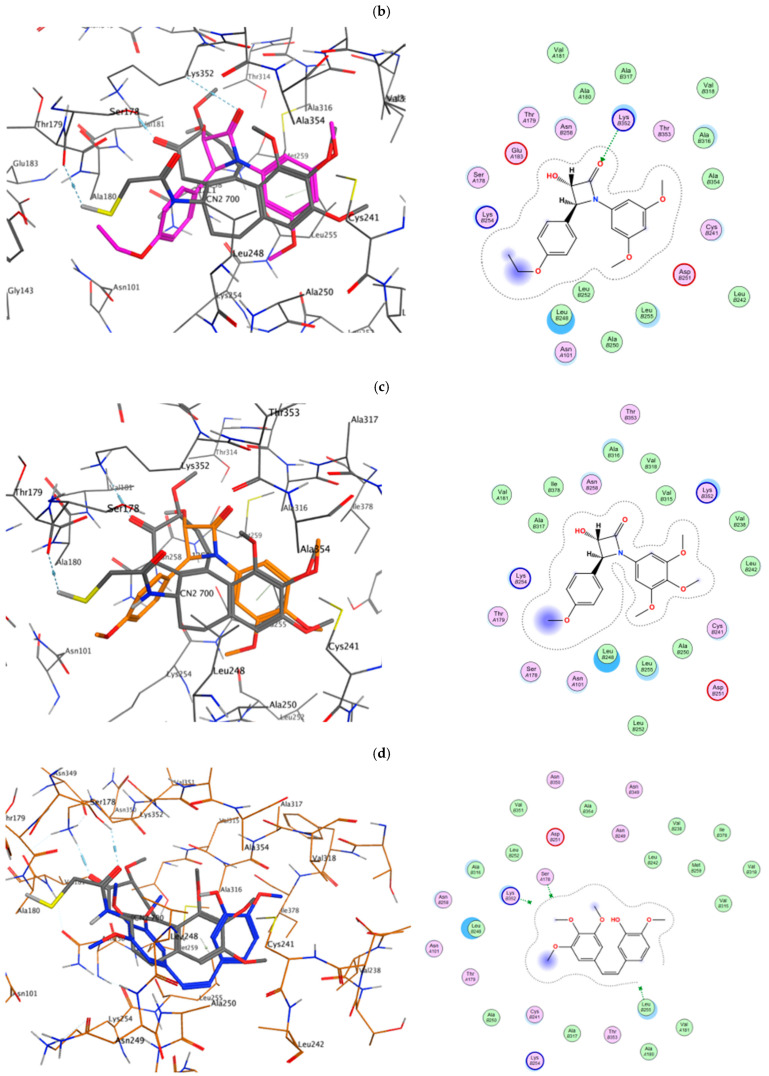
(**a**) A 3D image of the X-ray structure DAMA-colchicine on the left and the ligand interaction map on the right. An overlay of the X-ray structure of tubulin co-crystallised with DAMA-colchicine (colour: grey) (PDB entry 1Z2B) on the best ranked docked pose of (**b**) **12l** (colour: pink), (**c**) **12s** (colour: orange), and (**d**) **CA-4** (colour: blue), with 2D interaction maps of the binding site. Ligands are rendered as tubes and amino acids as lines. The dashes represent hydrogen bonds and hydrophobic interactions. Tubulin amino acids and DAMA-colchicine are coloured by atom type: carbon = grey, hydrogen = white, oxygen = red, nitrogen = blue, and sulphur = yellow. The atoms are coloured by element type. Key amino acid residues are labelled.

**Molecular docking:** The FRED v4.3.1.0 docking software [89], resulted in poses of **12l**, **12s**, and **CA-4** which resembled the pose occupied by DAMA-colchicine (**2**) (CN2) Figure 7. The FRED Chemgauss4 scores of **CA-4**, **12l**, and **12s** were −12.91 kcal/mol, −12.89 kcal/mol, and -12.58 kcal/mol, respectively, suggesting a stronger and more stable interaction for **12l** with the colchicine-binding site, when compared with **12s**. All three compounds overlaid their 3,5-dimethoxyphenyl ring (**12l**) and 3,4,5-trimethoxyphenyl ring CA-4 and **12s** on the 3,4,5-trimethoxyphenyl ring of DAMA-colchicine. Additionally, the docked pose of **12l** maintained the hydrogen bond with Lys352 as it did for DAMA-colchicine but with the carbonyl oxygen (Figure 7a); however, this was not observed for **12s** (Figure 7b).

**Molecular Dynamics:** To explore the dynamic behaviour of the compounds in a complex with tubulin and investigate the stability of the top docked poses, MD simulations were performed. Additionally, we also ran a 100 ns MD simulation on tubulin co-crystalised with DAMA-colchicine (CN2) to compare the MD runs of **CA-4**, **12l**, and **12s** against it. The MD simulations were run in triplicate, three of which showed similar profiles, so the most stable runs were selected to proceed further for analysis.

**Figure 8:**
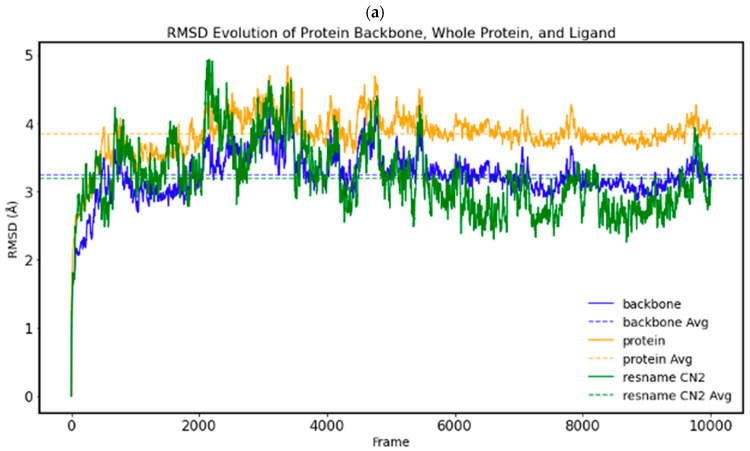

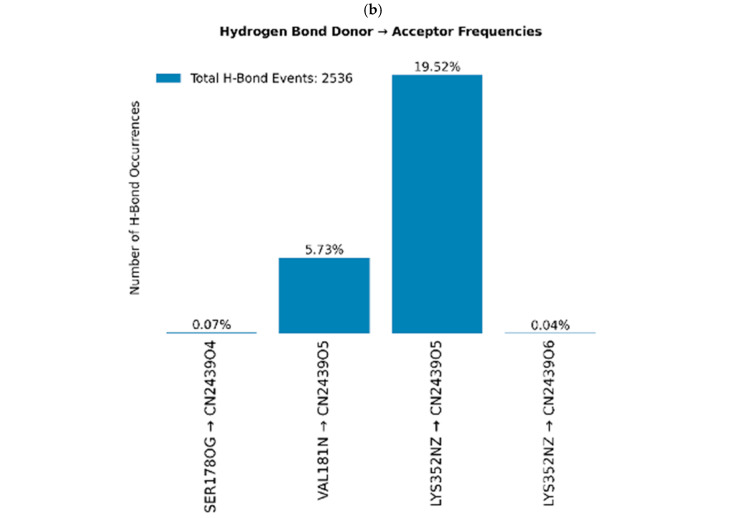
(**a**) The RMSD plot of protein, backbone, and CN2 (DAMA-colchicine) throughout the 100 ns MD run (10,000 frames). (**b**) Hydrogen bond plot between protein residues and CN2 with the contribution of each interaction (represented in percentage) during the MD run. The arrows depict the hydrogen bond donor atoms of protein residues and CN2 atoms.

From the MD simulations, we calculated the following: hydrogen bonds between the compounds and the protein, RMSD fluctuations of the complexes from their initial position, the RMSDs obtained by structurally overlaying the most representative frame from MD simulations over the predicted docked poses, and lastly, the structural overlays of the most representative frame from MD simulations of the two compounds to the representative frame of 1Z2B obtained from the MD simulation. The distance between the compounds and the co-crystallised ligand CN2 was calculated by considering the centre of mass. The MD run of the X-ray structure with co-crystallised CN2 (DAMA-colchicine) maintained a stable RMSD, indicating system stability. The CN2 molecule formed hydrogen bonds with Ser178, Val181, and Lys352 (Figure 8a,b).

**Figure 9:**
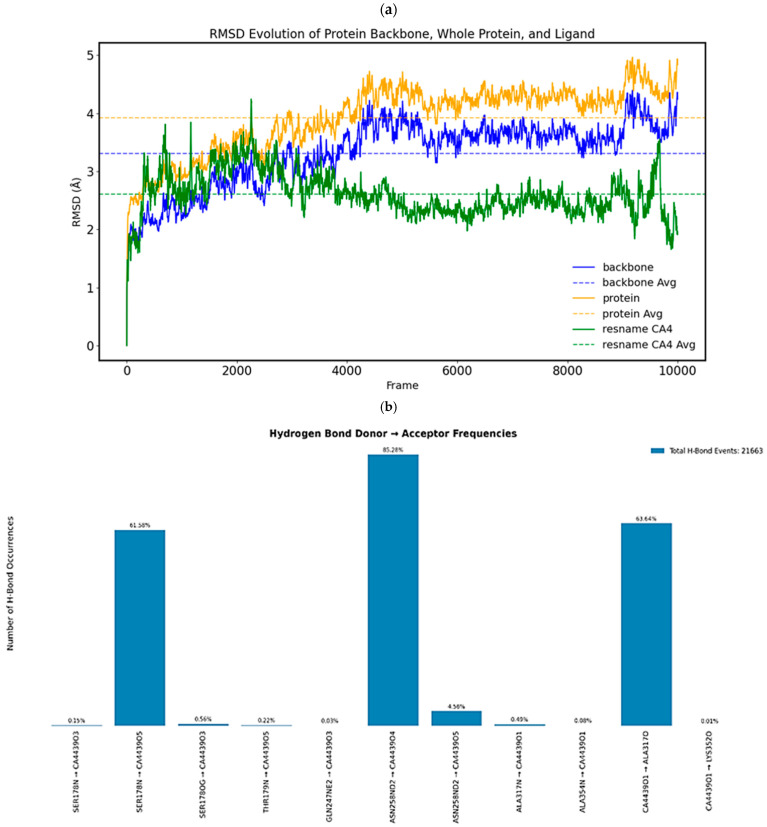

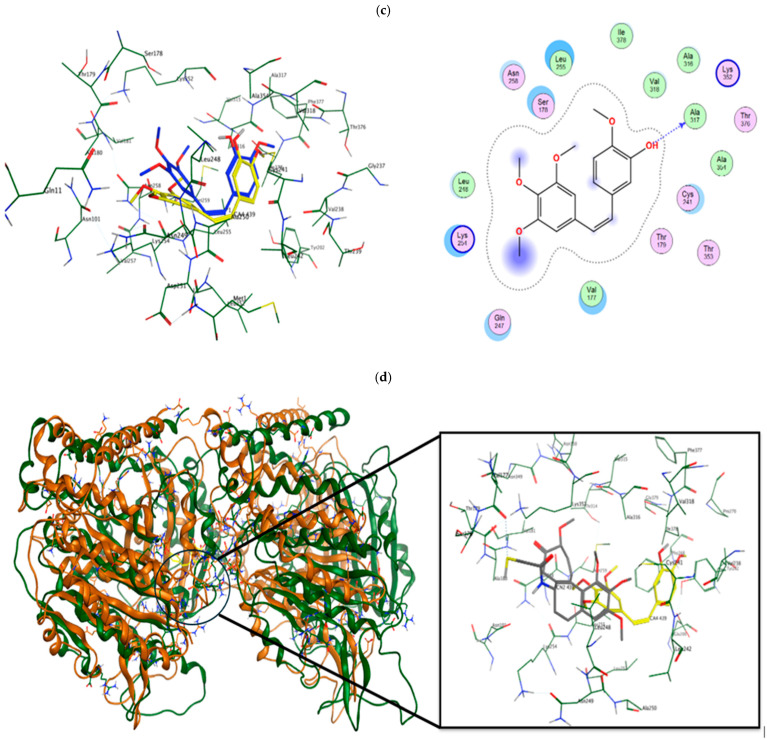
(**a**) The RMSD plot of the protein backbone, CA-4, throughout the 100 ns MD run (10,000 frames). (**b**) Hydrogen bond plot between protein residues and CA-4 with the contribution of each interaction (represented in percentage) during the MD run. The arrows depict the hydrogen bond donor atoms of protein residues and CA4 atoms. (**c**) Structural overlay between the MD representative frame with a 2D interaction map, (colour: yellow) and the MOE docked pose (colour: blue). (**d**) Structural overlay between the MD representative frame pose of CA4 (protein: brown ribbon), (colour: yellow) and the MD representative frame pose of 1Z2B, (protein: green ribbon), (co-crystallised ligand CN2, colour: grey).

For **CA-4**, there was minimal RMSD fluctuation of the protein chains (average 3.92 Å) and CA-4 (average 2.6 Å), suggesting a stable pose with slight conformational changes during the 100 ns MD run (Figure 9a). The hydrogen bond analysis revealed the presence of hydrogen bonds between ligand **CA-4**, and the tubulin residues Asn258 (85.28%), Ala317 (63.64%), and Ser178 (61.58%), as was true for CN2 (DAMA-colchicine) (Figure 9c). The structural overlay between the MD representative frame and the FRED docked pose showed slightly different binding modes of **CA-4** with an RMSD of 2.1 Å. Finally, the structural overlay between the MD representative frame pose of **CA-4** and the MD representative frame pose of 1Z2B (co-crystallised ligand CN2) revealed binding modes with a centre of mass distance of 1.8 Å (Figure 9d).

**Figure 10:**
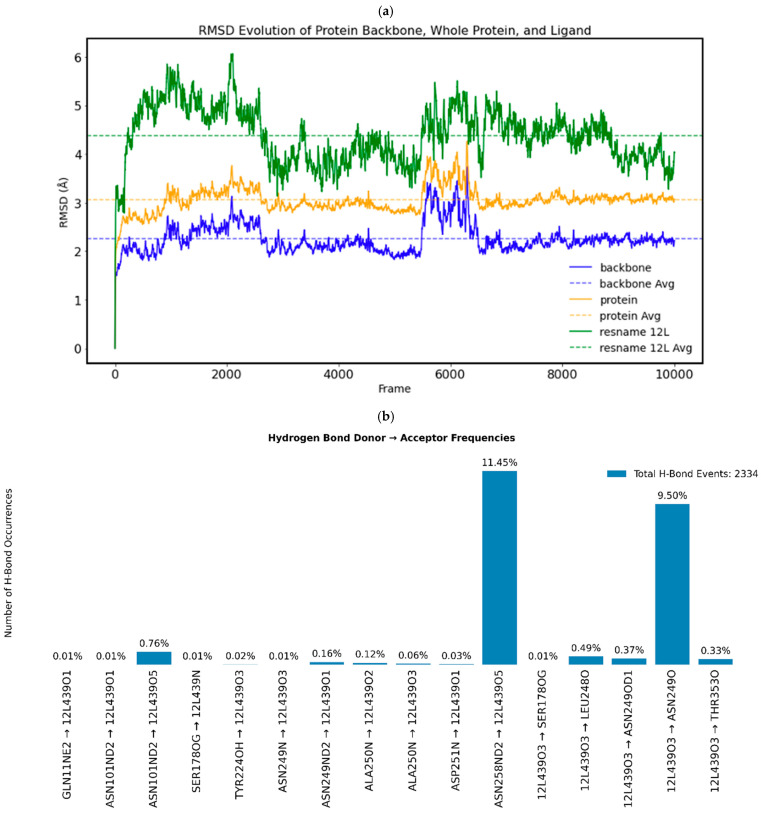

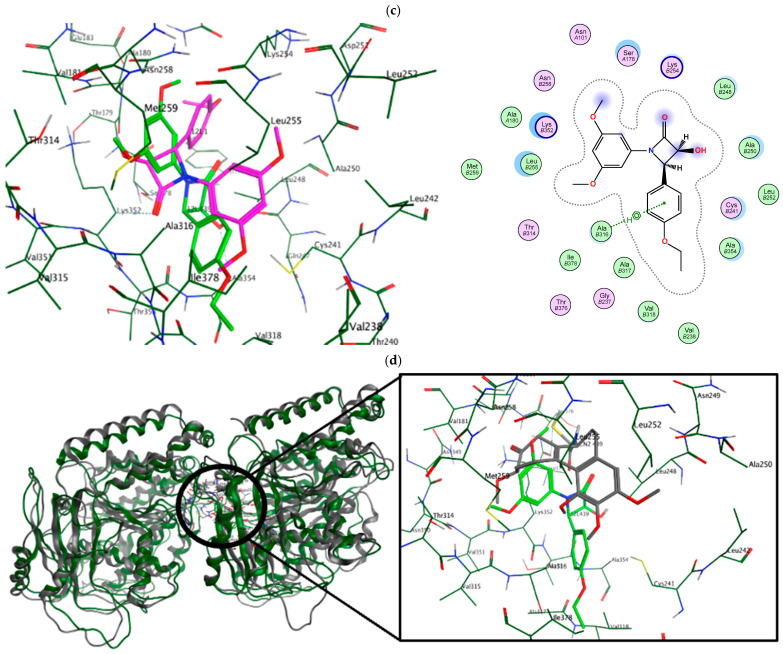
(**a**) The RMSD plot of the protein backbone, **12l**, throughout the 100 ns MD run (10,000 frames). (**b**) Hydrogen bond plot between protein residues and **12l** with the contribution of each interaction (represented in percentage) during the MD run. The arrows depict the hydrogen bond donor atoms of protein residues and **12l** atoms. (**c**) Structural overlay between the MD representative frame with a 2D interaction map (colour: green) and the FRED docked pose (colour: pink). (**d**) Structural overlay between the MD representative frame pose (protein: grey ribbon) of **12l** (colour: green) and the MD representative frame pose (protein: green ribbon) of 1Z2B (co-crystallised ligand CN2, colour: grey).

In the case of the *β*-lactam compound **12l** (3,5-dimethoxyphenyl Ring A), we observed that there was low RMSD fluctuation of the protein chains (average 3.1 Å) and **12l** (average 4.3 Å), suggesting a stable pose with slight conformational changes during the 100 ns MD run (Figure 10a). The hydrogen bond analysis showed the presence of hydrogen bonds between **12l** and the tubulin residues Asn101, Leu248, Asn249, Asn258, and Thr353 (Figure 10b). The compound **12l** maintained hydrogen bonds primarily with Asn249 (9.87%) and Asn258 (11.49%) but also with Ser178, similar to CN2 (DAMA-colchicine) but only for 0.01% of the MD run. The structural overlay between the MD representative frame and the FRED docked pose showed different binding modes of **12l** with an RMSD of 7.2Å; such a high RMSD was due to a 180° flipped pose (Figure 10c). Lastly, structural overlay between the MD representative frame pose of **12l** and the MD representative frame pose of 1Z2B (co-crystallised ligand CN2) showed different binding modes with a centre of mass distance of 3.8Å (Figure 10d).

**Figure 11:**
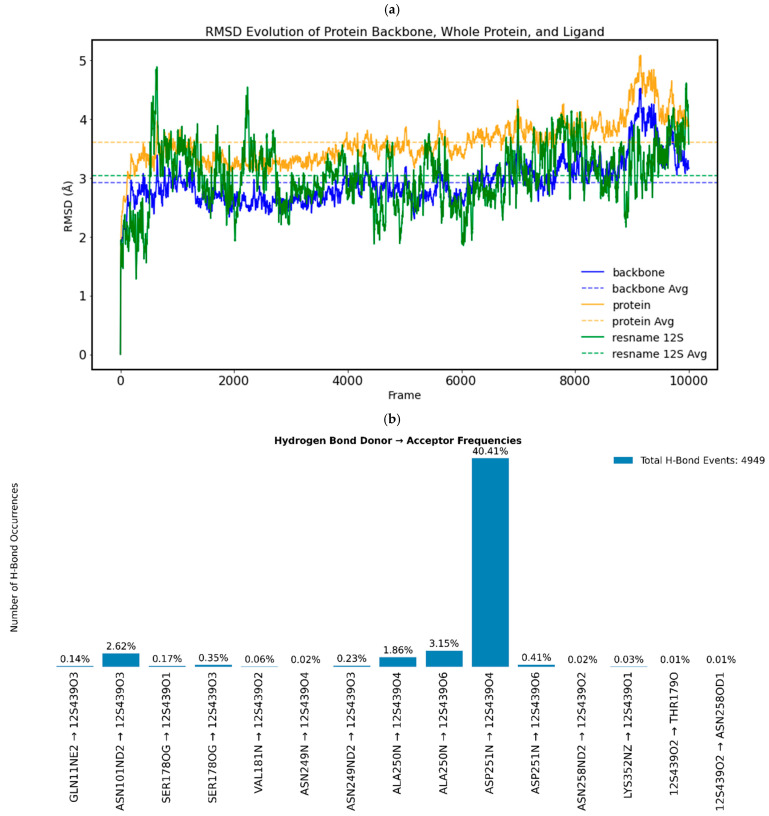

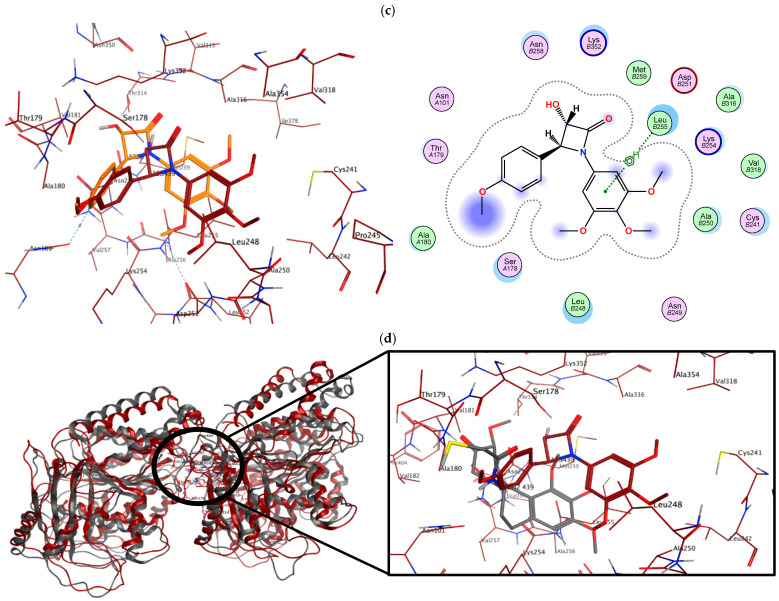
(**a**) The RMSD plot of the protein backbone, **12s**, throughout the 100 ns MD run (10,000 frames). (**b**) Hydrogen bond plot between protein residues and **12s** with the contribution of each interaction (represented in percentage) during the MD run. The arrows depict the hydrogen bond donor atoms of protein residues and **12s** atoms. (**c**) Structural overlay between the MD representative frame with a 2D interaction map (colour: brown) and the FRED docked pose (colour: orange). (**d**) Structural overlay between the MD representative frame pose (protein: grey ribbon) of **12s** (colour: brown) and the MD representative frame pose (protein: dark red ribbon) of 1Z2B (co-crystallised ligand CN2, colour: grey).

For *β*-lactam compound **12s** (3,4,5-trimethoxy Ring A), it was observed that there was low RMSD fluctuation of the protein chains (average 3.7 Å) and **12s** (average 3.1 Å), suggesting a stable pose throughout the 100 ns MD run (Figure 11a). A hydrogen bond analysis showed the presence of hydrogen bonds between **12s** and primarily the tubulin residues Asn101 (2.62%), Ala250 (5.01%), and Asp251 (40.82%). The compound **12s** also maintained hydrogen bonds with Ser178, Val181, and Lys352, similar to CN2, but only for a total of 0.61% of the MD run (Figure 11b). The structural overlay between the MD representative frame and the FRED docked pose showed similar binding modes of **12s**, with an RMSD of 2.6 Å (Figure 11c). In addition, structural overlay between the MD representative frame pose of **12s** and the MD representative frame pose of 1Z2B (co-crystallised ligand CN2) showed slightly different binding modes with a centre of mass distance of 4 Å (Figure 11d).

The molecular docking study on the two selected compounds **12s** and **12l** showed similar binding poses to DAMA-colchicine (CN2). Interestingly, the docked pose of **12l** maintained the hydrogen bond with Lys352, similar to the co-crystallised DAMA-colchicine (CN2). From the docking study, it can be hypothesised that the two compounds occupy the same binding pocket and make similar interactions to CN2. Since the docked poses looked promising, MD simulations were performed to investigate their dynamic behaviour and stability. The MD simulation of **12l** with tubulin showed a steady RMSD during the 100 ns run, which means the system was stable and that **12l** remained in the colchicine-binding site. However, there were small spikes in the RMSD of **12l**, suggesting it was undergoing slight conformational change and exploring different conformations. The hydrogen bond analysis showed that **12l** lost the hydrogen bond with Lys352 and instead, another hydrogen bond with Ser178 was observed, similar to CN2, but only for about 0.01% of the MD run. The compound **12l** explored a different orientation within the binding site and made new hydrogen bonds with Asn101, Leu248, Asn249, Asn258, and Thr353 (Figure 9b). To investigate the probable binding pose of **12l** in a complex with tubulin, the most representative frame was extracted. It showed that **12l** had acquired a different orientation and was flipped 180° from its docked pose, which is also reflected in the RMSD. This orientation aids in the formation of hydrophobic interactions between the 3,5-dimethoxyphenyl ring A and nearby hydrophobic residues such as Ala316 (Figure 9c). The MD representative frame, when compared to the MD representative frame of CN2, showed that **12l** remains near the site where CN2 binds but in a different orientation, indicating an alternative binding mode.

The MD simulation of **12s** in a complex with tubulin also had a stable RMSD throughout the 100 ns MD run. This indicated system stability and that **12s** remains within the colchicine-binding site. The hydrogen bond analysis showed that **12s** maintained hydrogen bonds with Ser178, Val181, and Lys352, similar to CN2, but only for about 0.61% of the MD run. The compound **12s** also made new hydrogen bonds with Asn101, Ala250, and Asp251 (Figure 10b). To investigate the probable binding pose of **12s** in a complex with tubulin, the most representative frame was extracted. It showed that **12s** had a similar orientation to its docked pose with a deviation, which is also reflected in the RMSD. This orientation also aids in the formation of hydrophobic interactions between the 3,4,5-trimethoxyphenyl ring and the nearby hydrophobic residues such as Leu255 (Figure 10c). Upon comparing the MD representative frame of **12s** to the MD representative frame of CN2 (DAMA-colchicine), it was observed that **12s** also remains near the site where CN2 binds but in a slightly different orientation, also pointing to an alternative binding mode.

By performing molecular docking and MD simulations, we have indicated that both *β*-lactam compounds **12l** and **12s** remain in the DAMA-colchicine-binding site by forming hydrogen and hydrophobic interactions with the tubulin backbone. It is clear from the hydrogen bond analysis obtained after an MD simulation that the central 4-methoxy substituent, depicted as 12S439O6 (Figure 10b) in **12s**, contributes to only 3.56% of hydrogen bond formations throughout the MD run. The major contribution to hydrogen bond formation is made by 3-methoxy, depicted as 12S439O4 (Figure 10b), contributing 42.29% of the bonds formed throughout the MD run in **12s**. In the case of **12l**, the major contribution to hydrogen bond formation is made by the 5-methoxy substituent, depicted as 12L439O5 (Figure 9b), with 12.21% of contributions throughout the MD run. We conclude that the central 4-methoxy substituent in **12s** does not contribute significantly to the formation of hydrogen bonds with the tubulin backbone. These results provide a possible explanation for the similar antiproliferative activities observed for both 3,4,5-trimethoxyphenyl Ring A compound **12s** and 3,5-dimethoxypheny Ring A compound **12l** and may rationalise the potent activity of the 3,5-dimethoxypheny Ring A series of synthesised azetidine-2-ones.

## 3. Materials and Methods

### 3.1. Chemistry

Infrared (IR) spectra were recorded as potassium bromide discs or films on NaCl plates using a Perkin Elmer FT-IR Spectrum 100 spectrometer (Perkin Elmer, Waltham, MA, USA). ^1^H and ^13^C nuclear magnetic resonance (NMR) spectra were recorded at 27 °C on a Bruker Avance DPX 400 spectrometer (Bruker, Billerica, MA, USA) (400.13 MHz for ^1^H; 100.61 MHz, for ^13^C) at 20 °C in CDCl_3_ (internal standard TMS) or DMSO-d_6_ School of Chemistry, Trinity College Dublin. ^1^H-NMR spectra in CDCl_3_ were assigned in relation to the TMS peak (0.00 δ) and ^13^C-NMR spectra were assigned with reference to the middle CDCl_3_ triplet peak at 77.00 ppm. Mass spectrometry with electrospray ionisation (ESI-MS) was performed using a liquid chromatography time-of-flight (TOF) mass spectrometer (Micromass LCT, Waters Ltd., Manchester, UK) with an electrospray ionization (ES) interface in positive ion mode and high resolution mass measurement accuracies of <±5 ppm. Melting points were determined on a Gallenkamp SMP 11 melting point apparatus and were uncorrected. R_f_ values for thin layer chromatography (TLC) were determined on silica gel Merck F-254 plates and products were homogenous on thin layer chromatography (TLC). Flash column chromatography was performed using Merck Kieselgel 60 (Merck, Rahway, NJ, USA) (particle size 0.040–0.063 mm) and also using a Biotage SP4 instrument (The Lab World Group, Hudson, MA, USA). Biotage Discover SP4 and CEM microwave synthesisers were used for the microwave experiments with the standard power setting and a maximum power of 300 watts, unless otherwise stated. Analytical high-performance liquid chromatography (HPLC) of products was performed using a Waters 2487 Dual Wavelength Absorbance detector, (Waters, Milford, MA, USA), Waters 1525 binary HPLC pump, Waters In-Line Degasser AF, Waters 717plus Autosampler, and a Varian Pursuit XRs C18 (Agilent, Santa Clara, CA, USA) reverse phase 150 × 4.6 mm chromatography column with detection at 254 nm and mobile phase acetonitrile (80%)–water (20%), at a flow rate (1 mL/min) over 10 min. Imines (**11a**) [61], (**11b)** [61], and azetidine-2-ones **12f**–**12i [61]**, **12j** [33], **12k** [36], **12s** [36], **12t [64]**, and **12u** [32] were prepared following the reported procedures.

#### 3.1.1. General Method I: Preparation of Imines **11a**–**11d**

3,5-Dimethoxyaniline (10 mmol) and the appropriately substituted benzaldehyde (10 mmol) were mixed in water (7 mL) over 30 min. The imine product was extracted with DCM, and the organic solution was dried (anhydrous Na_2_SO_4_) followed by evaporation of the solvent in vacuo.

(*E*)-N-(3,5-Dimethoxyphenyl)-1-(4-methoxyphenyl)methanimine **11a** was synthesized following general method I and was obtained from 4-methoxybenzaldehyde and 3,5-dimethoxyaniline as an oil [61], with a yield of 97%.

(*E*)-N-(3,5-Dimethoxyphenyl)-1-(4-ethoxyphenyl)methanimine **11b** was synthesized following general method I and was obtained from 4-ethoxybenzaldehyde and 3,5-dimethoxyaniline as an oil [61], with a yield of 85%.

(*E*)-N-(3,5-Dimethoxyphenyl)-1-(4-(methylthio)phenyl) methanimine **11c** was prepared using general method I above and was obtained from 4-methylthiobenzaldehyde and 3,5-dimethoxyaniline as an oil, with the following results: yield: 96%; purity (HPLC): 93%; IR ν_max_ (ATR):1596.77 cm^−1^ (C=N); ^1^H NMR (400 MHz, CDCl_3_): δ 2.51 (s, 3H, SCH_3_), 3.80 (s, 6H, OCH_3_), 6.35 (br. s., 3H, ArH), 7.28 (d, *J* = 8.5 Hz, 2 H, ArH), 7.77 (m, *J* = 7.9 Hz, 2H, ArH), and 8.37(s, 1H, CH=N); and ^13^C NMR (100 MHz, CDCl_3_): 15.06, 55.42, 98.18, 98.98, 125.65, 129.12, 132.61, 143.39, 154.26, and 159.84 (imine HC=NC) ppm. The HRMS: [M + H]^+^ calculated for C_16_H_18_NO_2_S, 288.1058, found 288.1068.

(*E*)-*N*-(4-(Ethylthio)benzylidene)-3,5-dimethoxyaniline **11d** was prepared using the general method I above and was obtained from 4-ethylthiobenzaldehyde and 3,5-dimethoxyaniline as an oil, with the following results: yield: 91%; purity (HPLC): 86%; IR ν_max_ (ATR): 1587.34 cm^−1^ (C=N); ^1^H NMR (400 MHz, CDCl_3_): δ 1.35 (t, *J* = 7.6 Hz, 3H, SCH_2_CH_3_), 3.00 (q, *J* = 7.3 Hz, 2H, SCH_2_CH_3_), 3.80 (s, 6H, OCH_3_), 6.32–6.36 (m, 3H, ArH), 7.33 (m, *J* = 8.5 Hz, 2H, ArH), 7.77 (m, *J* = 7.9 Hz, 2H, ArH), and 8.34–8.40 (m, 1H, CH=N); and ^13^C NMR (100 MHz, CDCl_3_): 14.03, 26.50, 55.13, 55.41, 93.75, 98.25, 98.98, 127.30, 129.14, 133.02, 141.96, 154.25, and 159.81 (imine HC=NC) ppm. The HRMS: [M + H]^+^ calculated for C_17_H_20_NO_2_S 302.1215, found 302.1232.

#### 3.1.2. General Method II: Synthesis of Azetidine-2-ones **12a**–**12j**

A solution of the appropriate imine (5 mmol) and acid chloride (7 mmol) in dry toluene (50 mL) was stirred at 0 °C under an inert atmosphere. The solution was gradually warmed to 100 °C, and dry triethylamine (TEA) (9 mmol) was slowly added. The reaction solution was heated at 100 °C for 5 h and then stirred at room temperature for 18 h, with monitoring by TLC. The mixture was then washed with water (2 × 100 mL) and dried with anhydrous Na_2_SO_4_. Evaporation of the solvent in vacuo was followed by purification of the crude product by flash chromatography over silica gel (eluent: 5:1; *n*-hexane–ethyl acetate).

1-(3,5-Dimethoxyphenyl)-4-(4-methoxyphenyl)-3-phenylazetidin-2-one **12a** was synthesised following the procedure in general method II above from imine **11a** and phenylacetyl chloride and afforded the product as a yellow powder, with the following results: yield: 30%; Mp: 123–124 °C; purity (HPLC): 95%; IR ν_max_ (ATR): 1761.1 (C=O) cm^−1^; ^1^H NMR (400 MHz, CDCl_3_): δ 3.69 (s, 6H, OCH_3_), 3.83 (s, 3H, OCH_3_), 4.92 (d, *J* = 1.8 Hz, 1H, H3), 5.06 (d, *J* = 1.2 Hz, 1H, H4), 6.18 (t, *J* = 2.1 Hz, 1H, ArH), 6.50 (d, *J* = 2.4 Hz, 2H, ArH), 6.85 (d, *J* = 8.5 Hz, 2H, ArH), 6.94 (d, *J* = 8.5 Hz, 2H, ArH), 7.00 (t, *J* = 7.3 Hz, 1H, ArH), 7.23 (d, *J* = 8.5 Hz, 2H, ArH), and 7.29–7.35 (m, 2H, ArH); and ^13^C NMR (100 MHz, CDCl_3_): 55.37, 64.01, 87.33, 96.27, 96.96, 114.83, 115.45, 122.28, 127.42, 127.78, 129.66, 138.60, 157.06, 160.23, 161.09, and 162.93 (C_2_, C=O) ppm. The HRMS calculated for C_24_H_23_NNaO_4_ [M + Na]^+^ 412.1525, found 412.1524.

1-(3,5-Dimethoxyphenyl)-4-(4-ethoxyphenyl)-3-phenylazetidin-2-one **12b** was synthesised following the procedure in general method II above, using imine **11b** and phenylacetyl chloride, and afforded the product as a yellow oil, with the following results: yield: 13%; purity (HPLC): 95%; IR ν_max_ (ATR): 1752.7 (C=O) cm^−1^; ^1^H NMR (400 MHz, CDCl_3_): δ 1.40 (t, *J* = 7.0 Hz, 3H, OCH_2_CH_3_), 3.65 (s, 3H, OCH_3_), 3.74 (s, 3H, OCH_3_), 4.01 (q, *J* = 6.9 Hz, 2H, OCH_2_CH_3_), 4.23 (s, 1H, H3), 4.85 (s, 1H, H4), 6.17 (d, *J* = 1.8 Hz, 1H, ArH), 6.54 (s, 2H, ArH), 6.86–6.93 (m, 2H, ArH), and 7.26–7.39 (m, 7H, ArH); and ^13^C NMR (100 MHz, CDCl_3_): 14.79, 55.31, 63.51, 63.74, 65.06, 95.8, 115.16, 127.82, 129.14, 134.77, 139.13, 159.25, and 161.09 (C_2_, C=O) ppm. The HRMS calculated for C_25_H_25_NNaO_4_ [M + Na]^+^ 426.1681, found 426.1694.

1-(3,5-Dimethoxyphenyl)-4-(4-(methylthio)phenyl)-3-phenylazetidin-2-one **12c** was synthesised following the procedure in general method II above from imine **11c** and phenylacetyl chloride and afforded the product as a yellow powder, with the following results: yield: 20%; Mp: 131–132 °C; purity (HPLC): 96%; IRν_max_ (ATR): 1761.2 (C=O) cm^−1^; ^1^H NMR (400 MHz, CDCl_3_): δ 2.47 (s, 3H, SCH_3_), 3.71 (s, 3H, OCH_3_), 3.83 (s, 3H, OCH_3_), 4.21 (d, *J* = 2.5 Hz, 1H, H-3), 4.85 (d, *J* = 2.5 Hz, 1H, H-4), 6.17 (dd, *J* = 2.3 Hz, 1H, ArH), 6.52 (d, *J* = 2.1 Hz, 2H, ArH), 7.26 (s, 2H, ArH), 7.28–7.33 (m, 4H, ArH), and 7.35 (d, *J* = 5.8 Hz, 3H, ArH); and ^13^C NMR (100 MHz, CDCl_3_): 15.55, 55.35, 63.66, 65.05, 95.80, 126.34, 127.02, 129.03, 134.56, 138.99, 139.33, and 161.13 (C_2_, C=O) ppm. The HRMS calculated for C_24_H_24_O_3_S [M + H]^+^ 406.1477, found 406.1480.

1-(3,5-Dimethoxyphenyl)-4-(4-methoxyphenyl)-3-phenoxyazetidin-2-one **12d** was obtained following general method II above using imine **11d** and phenoxyacetyl chloride to afford the product as an oil, leading to the following results: yield: 15%; purity (HPLC): 96%; IR ν_max_ (ATR): 1762.5 (C=O) cm^−1^; ^1^H NMR (400 MHz, CDCl_3_): δ 3.71 (s, 6H, 2 × OCH_3_), 3.81 (s, 3H, OCH_3_), 4.23 (d, *J* = 1.8 Hz, 1H, H-4), 4.85 (d, *J* = 1.8 Hz, 1H, H-3), 6.14–6.19 (m, 1H, ArH), 6.54 (d, *J* = 2.4 Hz, 2H, ArH), 6.92 (d, *J* = 8.5 Hz, 2H, ArH), and 7.29–7.41 (m, 7H, Ar-H); and ^13^C NMR (100 MHz, CDCl_3_): 55.32, 63.71, 65.08, 95.77, 96.40, 114.65, 127.39, 127.82, 128.98, 134.74, 157.30, and 161.07 (C2, C=O) ppm. The HRMS calculated for C_24_H_23_NNaO_5_ [M + Na]^+^ 428.1474, found 428.1478.

1-(3,5-Dimethoxyphenyl)-4-(4-ethoxyphenyl)-3-phenoxyazetidin-2-one **12e** was obtained following general method II above from imine **11b** and phenoxyacetyl chloride to afford the product as an orange powder, leading to the following results: yield: 5%; Mp: 95–96 °C; purity (HPLC): 97%; IRν_max_ (ATR): 1745.8 (C=O) cm^−1^; ^1^H NMR (400 MHz, CDCl_3_): **12e** *trans* δ 1.41 (t, *J* = 7.0 Hz, 3H, OCH_2_CH_3_), 3.68 (s, 3H, OCH_3_), 3.73 (s, 3H, OCH_3_), 4.03 (q, *J* = 6.7 Hz, 2H, OCH_2_CH_3_), 4.90 (d, *J* = 1.8 Hz, 1H, H-4), 5.05 (d, *J* = 1.2 Hz, 1H, H-3), 6.17 (t, *J* = 2.1 Hz, 1H, ArH), 6.49 (d, *J* = 2.4 Hz, 2H, ArH), 6.84 (d, *J* = 7.9 Hz, 2H, ArH), 6.92 (m, *J* = 8.5 Hz, 2H, ArH), 6.98 (t, *J* = 7.3 Hz, 1H, ArH), 7.20–7.26 (m, 2H, ArH), and 7.27–7.33 (m, 2H, ArH); ^13^C NMR (100 MHz, CDCl_3_): 14.77, 55.32, 63.55, 64.01, 96.21, 96.92, 115.40, 122.22, 127.18, 129.60, 138.57, 157.01, 159.59, and 161.03 (C2, C=O) ppm; ^1^H NMR (400 MHz, CDCl_3_): **12e** *cis* δ 1.35 (t, *J* = 7.0 Hz, 3H), 3.70 (s, 6H), 3.95 (qd, *J* = 6.92, 1.8 Hz, 2H), 5.28 (d, *J* = 4.9 Hz, 1H), 5.49 (d, *J* = 4.9 Hz, 1H), 6.18 (t, *J* = 2.1 Hz, 1H), 6.55 (d, *J* = 2.4 Hz, 2H), 6.73–6.80 (m, 4H), 6.90 (t, *J* = 7.6 Hz, 1H), 7.09–7.18 (m, 2H), 7.24–7.29 (m, 2H); and ^13^C NMR (101 MHz, CDCl_3_): **12e** *cis* 14.74 (s, 1 C), 55.34 (s, 1 C), 62.03 (s, 1 C), 63.34 (s, 1 C), 76.69 (s, 1 C), 77.00 (s, 1 C), 77.32 (s, 1 C), 81.05 (s, 1 C), 96.20 (s, 1 C), 96.82 (s, 1 C), 114.37 (s, 1 C), 115.70 (s, 1 C), 122.13 (s, 1 C), 124.12 (s, 1 C), 129.23 (s, 1 C), 129.35 (s, 1 C), 138.54 (s, 1 C), 156.97 (s, 1 C), 159.24 (s, 1 C), 161.06 (s, 1 C), and 163.34 (s, 1 C) ppm. The HRMS calculated for C_25_H_25_NNaO_5_ [M + Na]^+^ 442.1630, found 442.1643.

3-Chloro-1-(3,5-dimethoxyphenyl)-4-(4-methoxyphenyl) azetidin-2-one **12f [61]** was synthesised as we previously discussed following general method II above from imine **11a** and chloroacetyl chloride to afford the product as a yellow powder, with the following results: yield: 8%, Mp: 93–94 °C, and purity (HPLC): 94%.

3-Chloro-1-(3,5-dimethoxyphenyl)-4-(4-ethoxyphenyl)azetidin-2-one **12g [61]** was synthesised as we previously reported following general method II above from imine **11b** and chloroacetyl chloride to afford the product as an oil with the following results: yield: 20%, Mp: 129–130 °C, and purity (HPLC): 94%.

3,3-Dichloro-1-(3,5-dimethoxyphenyl)-4-(4-methoxyphenyl) azetidin-2-one **12h [61]** was synthesised as we previously reported following method II above from imine **11a** and dichloroacetyl chloride to afford the product as a yellow oil, with the following results: yield: 13%, and purity (HPLC): 95%.

3,3-Dichloro-1-(3,5-dimethoxyphenyl)-4-(4-ethoxyphenyl) azetidin-2-one **12i [61]** was synthesised as we previously reported following method II above from imine **11b** and dichloroacetyl chloride to afford the product as a brown oil, which crystallised on standing and had a yield of 7%, and purity (HPLC) of 96%.

1-(3,5-Dimethoxyphenyl)-4-(4-methoxyphenyl)-3-vinylazetidin-2-one **12j [33]** was synthesised as we previously reported following method II above from imine **11a** and crotonyl chloride to afford the product as a brown oil with a yield of 17%, and purity (HPLC) of 92%.

#### 3.1.3. General Method III: Synthesis of 3-Unsubstituted Azetidine-2-ones **12o**–**12r**

A mixture of activated zinc powder (9 mmol) and trimethylchlorosilane (7 mmol) in anhydrous benzene (4 mL) was heated at 40 °C for 15 min. The mixture was then placed in a microwave reactor and heated at 100 °C for 2 min and then cooled to room temperature. The required imine (2 mmol) was then added together with ethyl bromoacetate (5 mmol), and the mixture was then heated in the microwave reactor for 30 min at 100 °C. The reaction mixture was cooled and filtered; the solution was diluted with dichloromethane (30 mL) and washed with an ammonium chloride solution (20 mL, saturated), ammonium hydroxide (20 mL, 25%), HCl (10%, 40 mL), and water (40 mL). The organic solution was dried (anhydrous Na_2_SO_4_), followed by evaporation of the solvent and purification of the crude product by flash column chromatography over silica gel (eluent: hexane–ethyl acetate gradient).

1-(3,5-Dimethoxyphenyl)-4-(4-methoxyphenyl)azetidin-2-one **12o** was obtained following the general Reformatsky method III from imine **11a** and ethyl 2-bromoacetate to yield the product as a brown oil, which crystallised on standing, with the following results: yield: 19%; purity (HPLC): 97%; IR ν_max_ (ATR): 1751.2 (C=O) cm^−1^; ^1^H NMR (400 MHz, CDCl_3_): δ 2.88 (dd, *J* = 15.3, 2.4 Hz, 1H, H3), 3.49 (dd, *J* = 15.3, 6.1 Hz, 1H, H3), 3.68 (s, 6H, 2 × OCH_3_), 3.78 (s, 3H, OCH_3_), 4.89 (dd, *J* = 5.8, 2.8 Hz, 1H, H4), 6.12 (t, *J* = 2.4 Hz, 1H, H4), 6.46 (d, *J* = 2.4 Hz, 2H, ArH), 6.86 (d, *J* = 3.1 Hz, 1H, ArH), 6.88 (d, *J* = 3.1 Hz, 1H, ArH), 7.27 (d, *J* = 3.7 Hz, 1H, ArH), and 7.29 (d, *J* = 3.1 Hz, 1H, ArH); and ^13^C NMR (100 MHz, CDCl_3_): 46.98, 53.98, 55.28, 60.79, 95.39, 96.12, 113.88, 114.51, 126.93, 127.13, 130.02, 139.41, 161.03, and 164.88 (C_2_, C=O) ppm. The HRMS calculated for C_18_H_19_NNaO_4_ [M + Na]^+^ 336.1212, found 336.1230.

1-(3,5-Dimethoxyphenyl)-4-(4-ethoxyphenyl) azetidin-2-one **12p** was obtained following general Reformatsky method III from imine **11b**, and ethyl 2-bromoacetate to afford the product as a yellow powder, with the following results: yield: 10%; Mp: 96–97 °C; purity (HPLC): 99%; IRν_max_ (ATR): 1755.2 (C=O) cm^−1^; ^1^H NMR (400 MHz, CDCl_3_): δ 1.39 (t, *J* = 6.7 Hz, 3H, OCH_2_CH_3_), 2.88 (dd, *J* = 15.3, 3.1 Hz, 1H, H3), 3.48 (dd, *J* = 15.3, 6.1 Hz, 1H, H3), 3.68 (s, 6H, 2 × OCH_3_), 3.96–4.02 (m, 2H, OCH_2_CH_3_), 4.89 (dd, *J* = 5.5, 2.4 Hz, 1H, H4), 6.12 (t, *J* = 2.14Hz, 1H, ArH), 6.47 (d, *J* = 2.4 Hz, 2H, ArH), 6.84–6.88 (m, 2H, ArH), and 7.24–7.27 (m, 2H, ArH); and ^13^C NMR (100 MHz, CDCl_3_): 14.77, 46.98, 54.02, 55.29, 63.47, 96.13, 114.45, 115.02, 126.90, 127.12, 129.83, 139.43, 159.08, 161.02, and 164.90 (C2, C=O) ppm. The HRMS calculated for C_19_H_21_NNaO_4_ [M + Na]^+^ 350.1368, found 350.1366.

1-(3,5-Dimethoxyphenyl)-4-(4-(methylthio)phenyl)azetidin-2-one **12q** was prepared following general Reformatsky method III from imine **11c** and ethyl 2-bromoacetate to afford the product as a yellow powder, with the following results: yield: 21%; Mp: 95–96 °C; purity (HPLC): 97%; IRν_max_ (ATR): 1753.7 (C=O) cm^−1^; ^1^H NMR (400 MHz, CDCl_3_): δ 2.44 (s, 3H, SCH_3_), 2.86 (dd, *J* = 15.3, 3.1 Hz, 1H, H-3), 3.49 (dd, *J* = 15.3, 6.1 Hz, 1H, H-3), 3.68 (s, 6H, 2 × OCH_3_), 4.89 (dd, *J* = 5.8, 2.6 Hz, 1H, H-4), 6.13 (t, *J* = 2.1 Hz, 1H, ArH), 6.45 (m, *J* = 2.4 Hz, 2H, ArH), 7.19–7.23 (m, 3H, ArH), and 7.27 (d, *J* = 1.2 Hz, 1H, ArH); and ^13^C NMR (100 MHz, CDCl_3_): 14.14, 43.22, 46.87, 53.93, 55.31, 60.86, 69.91, 96.12, 126.21, 126.35, 126.68, 126.91, 134.79, 139.07, 139.29, 161.06, and 164.65 (C2, C=O) ppm. The HRMS calculated for C_18_H_19_NNaO_3_S [M + Na]^+^ 352.0983, found 352.0984.

1-(3,5-Dimethoxyphenyl)-4-(4-(ethylthio)phenyl)azetidin-2-one **12r** was prepared following general Reformatsky method III from imine **11d** and ethyl 2-bromoacetate to afford the product as a brown solid, with the following results: yield: 12%; Mp: 89–90 °C; purity (HPLC): 95%; IR ν_max_ (ATR): 1755.4 (C=O) cm^−1^; ^1^H NMR (400 MHz, CDCl_3_): δ 1.27–1.32 (m, 3H, SCH_2_CH_3_), 2.88–2.96 (m, 3H, SCH_2_CH_3_, H3), 3.50 (dd, *J* = 15.0, 5.8 Hz, 1H, H3), 3.68 (s, 6H, OCH_3_), 4.90 (dd, *J* = 5.5, 3.1 Hz, 1H, H4), 6.09–6.14 (m, 1H, ArH), 6.45 (d, *J* = 2.4 Hz, 2H, ArH), and 7.25–7.29 (m, 4H, ArH); ^13^C NMR (100 MHz, CDCl_3_): 14.23, 27.27, 46.87, 53.94, 55.3, 96.17, 126.35, 129.04, 135.48, 137.44, 139.29, 161.06, and 164.62 (C2, C=O) ppm. The HRMS calculated for C_19_H_21_NNaO_3_S [M + Na]^+^ 366.1140, found 366.1145.

#### 3.1.4. General Method IV: Preparation of 3-Hydroxyazetidin-2-ones (**12k**–**12n**)

To a stirred solution of the appropriate imine **11a**–**11d** (5 mmol) in toluene (50 mL), 2-acetoxyacetyl chloride (7 mmol) was added. The solution was maintained under nitrogen at 0 °C during the addition. The solution then slowly warmed to 100 °C and TEA (9 mmol) was added gradually to the reaction mixture over 10 min; the mixture was maintained at 100 °C for 5 h, then cooled, washed with water (50 mL × 2), dried over anhydrous Na_2_SO_4_, and the solvent was removed in vacuo. The crude product was purified by flash chromatography over silica gel (eluent: *n*-hexane–ethyl acetate, 2:1). A solution of the crude product in methanol (30 mL) was treated with hydrazine dihydrochloride (5 mmol) at 0 °C and under nitrogen. TEA (9 mmol) was added slowly over 10 min. The mixture was then warmed to room temperature and then heated at reflux for 2–4 h until the reaction was complete. Evaporation of the solvent afforded the crude product, which was treated with a saturated solution of KHSO_4_, extracted with ethyl acetate (3 × 50 mL, and dried over anhydrous Na_2_SO_4_. Evaporation of the solvent at reduced pressure afforded the crude residue, which was purified by flash chromatography over silica gel (eluent: *n*-hexane–ethyl acetate, 6:1) to give the desired product.

1-(3,5-Dimethoxyphenyl)-3-hydroxy-4-(4-methoxyphenyl)azetidin-2-one **12k** [36] was synthesized following general procedure IV using imine **11a**. The product was afforded as a yellow powder, with the following results: yield: 65%; Mp: 123–124 °C; IR (NaCl, film) ν_max_: 3399.77 (OH), 1740.10 (C=O, *β*-lactam) cm^−1^; ^1^H NMR (400 MHz, CDCl_3_): δ 3.66 (s, 6 H, 2 × OCH_3_), 3.78 (s, 3 H, OCH_3_), 4.63–4.71 (m, 1H, OH), 4.78 (d, *J* = 1.8 Hz, 1H, H3), 6.14 (t, *J* = 2.1 Hz, 1H, H4), 6.43 (d, *J* = 1.8 Hz, 3H, H2′, H6′, H4′), 6.84–6.91 (m, 2H, H_3_′, H5′), and 7.19–7.24 (m, 2H, H_2_″, H_6_″); and ^13^C NMR (100 MHz, CDCl_3_): 55.29 (OCH_3_), 60.42 (C3), 65.44 (C4), 96.21, 96.77, 114.55, 127.34, 133.98, 137.54, 160.99, and 164.75 (C2) ppm. The HRMS (*m*/*z*) calculated for C_18_H_19_NNaO_5_, [M + Na]^+^: 352.1155, found 352.1156.

1-(3,5-Dimethoxyphenyl)-4-(4-ethoxyphenyl)-3-hydroxyazetidin-2-one **12l** was synthesized following general procedure IV using imine **12b**. The product was afforded as a pale-yellow powder, with the following results: yield: 48%; Mp: 130–132 °C; purity (HPLC): 100%; IR (NaCl, film) ν_max_: 3429.14 (OH), 1736.34 (C=O, *β*-lactam); ^1^H NMR (400 MHz, CDCl_3_): δ 1.39 (t, *J* = 7.0 Hz, 4H, CH_2_CH_3_), 3.67 (s, 6H, 2 × OCH_3_), 4.00 (q, *J* = 6.7 Hz, 2H, CH_2_CH_3_), 4.68 (d, *J* = 1.8 Hz, 1H, H3), 4.77 (d, *J* = 1.8 Hz, 1H, H4), 6.14 (t, *J* = 2.1 Hz, 1H, H4′), 6.43 (d, *J* = 2.4 Hz, 2H, H2′, H6′), 6.82–6.89 (m, 2H, H3′, H5′), and 7.19–7.23 (m, 2H, H2″, H6″); ^13^C NMR (100 MHz, CDCl_3_): 14.75 (CH_2_CH_3_), 55.29 (OCH_3_), 60.65 (C3), 63.56 (OCH_2_CH_3_), 66.43(C4), 96.20, 96.76, 115.06, 127.32, 160.99, and 167.11 (C2) ppm. The HRMS calculated for C_19_H_22_NO_5_, 344.1498 [M + H]^+^, found 344.1504.

1-(3,5-Dimethoxyphenyl)-3-hydroxy-4-(4-(methylthio)phenyl)azetidin-2-one **12m** was synthesized using method IV above from imine **11c**. The product was afforded as a pale-yellow powder, with the following results: yield: 41%, purity (HPLC): 99%; Mp: 142–143 °C; IR (NaCl, film) ν_max_: 3411.43 (OH), 1735.66 (C=O, *β*-lactam) cm^−1^; ^1^H NMR (400 MHz, CDCl_3_): δ 2.45 (s, 3H, SCH_3_), 3.66 (s, 6H, 2 × OCH_3_), 3.70 (s, 1H, OH), 3.97 (d, *J* = 5.5 Hz, 1H, H3), 4.66 (d, *J* = 4.9 Hz, 1H, H4), 6.09–6.18 (m, 1H, H4′), 6.40 (d, *J* = 1.8 Hz, 2H, H2′, H6′), and 7.17–7.22 (m, 4H, Ar-H); and ^13^C NMR (100 MHz, CDCl_3_): 15.42 (SCH_3_), 55.29, 55.37 (OCH_3_), 60.51 (C3), 65.45 (C4), 83.59, 96.25, 96.78, 126.86, 127.87, 132.53, 138.38, 139.42, 161.00, and 166.81 (C2) ppm. The HRMS calculated for C_18_H_19_NNaO_4_S, 368.0932 [M + Na]^+^, found 398.0932.

4-(4-(Ethylthio)phenyl)-3-hydroxy-1-(3,5-dimethoxyphenyl) azetidin-2-one **12n** was prepared using general procedure IV above from imine **11d**. The product was afforded as a brown oil, with the following results: yield: 32%; purity (HPLC): 100%; IR (NaCl, film) ν_max_: 3412.80 (OH), 1747.92 (C=O, *β*-lactam) cm^−1^; ^1^H NMR (400 MHz, CDCl_3_): δ 1.29 (t, *J* = 7.0 Hz, 3H, SCH_2_CH_3_), 2.93 (d, *J* = 7.3 Hz, 2H, SCH_2_CH_3_), 3.67 (s, 6H, 2 × OCH_3_), 4.78 (s, 1H, H4), 6.47 (s, 2H, H2′, H6′), and 7.27–7.32 (m, 4H, Ar-H); and ^13^C NMR (100 MHz, CDCl_3_): 14.15 (SCH_2_CH_3_), 27.22 (SCH_2_CH_3_), 55.99 (OCH_3_), 60.91(C3), 65.50 (C4), 95.29, 110.00, 116.02, 126.62, 129.01, 133.03, 133.22, 138.03, 153.45, 162.45, and 165.98 (C2) ppm. The HRMS calculated for C_19_H_22_NNaO_5_, 382.1268 [M + Na]^+^, found 382.1261.

3-Hydroxy-4-(4-methoxyphenyl)-1-(3,4,5-trimethoxyphenyl) azetidin-2-one **12s** was prepared as previously reported following general procedure IV from imine (*E*)-*N*-(3,4,5-trimethoxyphenyl)-1-(4-methoxyphenyl)methanimine to give the product as a white powder, with a yield of 30%, an Mp of 135–136 °C [64], and a purity (HPLC) of 97.5%.

4-(4-Ethoxyphenyl)-3-hydroxy-1-(3,4,5-trimethoxyphenyl) azetidin-2-one **12t** [64] was prepared as we previously reported using general procedure IV above from imine (*E*)-*N*-(3,4,5-trimethoxyphenyl)-1-(4-methoxyphenyl)methanimine to give the product as a white solid, with a yield of 20%, an Mp of 128–129 °C, and a purity (HPLC) of 100%.

3-Hydroxy-4-(4-methoxy-3-methylphenyl)-1-(3,4,5-trimethoxyphenyl)azetidin-2-one **12u** [32] was obtained as we previously reported using general procedure IV above from imine (*E*)-*N*-(3,4,5-trimethoxyphenyl)-1-(4-(methoxy-3-methyl)phenyl) methanimine to give the product as a grey crystalline solid, with a yield of 40%, an Mp of 146 °C, and a purity (HPLC) of 96%.

### 3.2. Biochemical Evaluation: Materials and Methods

#### 3.2.1. Cell Culture

All biochemical materials, reagents, and growth media for cell cultures were purchased from BD Biosciences (Edmund Halley Road, Oxford, UK). The biochemical assays were performed in triplicate and mean values were reported. The MCF-7 cell line (human breast carcinoma) was obtained from the European Collection of Animal Cell Cultures (ECACC). The cells were cultured in an Eagles minimum essential medium with 10% foetal bovine serum, 2 mM L-glutamine, and 100 mg/mL penicillin/streptomycin and supplemented with 1% non-essential amino acids. HT-29 cells (human colon adenocarcinoma) from the ECACC were grown in DMEM (Dulbecco’s modified Eagle’s medium) GlutaMAX media and supplemented with 10% foetal bovine serum (FBS). Cells were maintained at 37 °C in 5% CO_2_ in a humidified incubator and sub-cultured 3 times/week by trypsinisation.

#### 3.2.2. Cell Viability Assay

Cells were seeded at a density of 5x10^3^ cells/well (MCF-7 cells) and 10^4^ cells/well (HT-29 cells) in triplicate in 96-well plates. After 24 h, cells were then treated with the medium alone, or a vehicle [1% ethanol (*v*/*v*)] or with selected dilutions of control CA-4 or the synthesised azetidine-2-one compounds in the concentration range 1 nM–50 μM, as previously reported [64]. Cell proliferation for MCF-7 cells was analysed using the Alamar Blue assay (Invitrogen Corp., Waltham, MA, USA). After 72 h, Alamar Blue [10% (*v*/*v*)] was added to each well and the plates were incubated in the dark for 3–5 h at 37˚C. Fluorescence was determined with a 96-well fluorimeter with excitation (530 nm) and emission (590 nm); results were presented as viability (%) relative to vehicle control (100%). Fluorescence was read using the BMG-Labtech, FLUOstar Optima plate reader (Ortenberg, Germany) and the Gemini Spectramax plate reader (Molecular Devices, San Jose, CA, USA). IC_50_ values were obtained from the dose response curves using GraphPad PRISM (version 5) software (Graphpad Software Inc., San Diego, CA, USA). Experiments were performed in triplicate on at least three separate occasions.

#### 3.2.3. Lactate Dehydrogenase Cytotoxicity Assay

The cytotoxicity of the compounds was determined using the CytoTox 96 non-radioactive cytotoxicity assay (Promega, Madison, WI, USA) [76], as previously reported [33]. The MCF-7 cells (seeding density 5 × 10^3^ cells/well) in the 96-well plates were incubated for 24 h. The cells were treated with test compounds **12a**, **12b**, **12c**, **12f**, **12j**, and CA-4 (10 μM), as performed for the cell viability assay above. At 72 h, 20 μL of 'lysis solution (10X)' was added to the control wells. After a further 1 h of incubation, the supernatant (50 μL) was removed from each well and transferred to a 96-well plate. Cytotox 96R Reagent (50 μL) was added to each well and the plate was retained in darkness at 20 °C for 30 min. A total of 50 μL of ‘stop solution’ was then added to each well and the absorbance was determined at 490 nm using a Dynatech MR5000 plate reader (Dynex Technologies, Chantilly, VA, USA), to obtain cell death (%) at 10 μM.

### 3.3. Crystallography

Crystals for **12i**–**12s** were mounted on a MiTeGen micromount with NVH immersion oil. Data were collected from a shock-cooled single crystal at 100(2) K on an APEX2 Kappa Duo (Bruker AXS, Karlsruhe, Germany) diffractometer with a standard sealed X-ray tube using a graphite as monochromator (compounds **12i**–**12p**) and a microfocus sealed X-ray tube using a mirror optics (compound **12u**) as a monochromator and an APEX2 detector. The diffractometer was equipped with a Cobra (Oxford Cryosystems Ltd., Oxford, UK) low temperature device and used Mo*K_α_* (λ = 0.71073 Å) and Cu*K_α_* (λ = 0.71073 Å) radiation. All data were integrated with SAINT v8.434A and multi-scan absorption correction using SADABS 2014/2 was applied [90,91]. Structures were solved by dual methods with SHELXT v2014/5 [92] and refined by full-matrix least-squares methods against *F*^2^ using SHELXL [93].

Crystals for **12o** were mounted on a glass fibre using superglue and data were collected at 93(2) K using a Rigaku Saturn 724 instrument (Mo Kα radiation, λ = 0.71073 Å) equipped with a Rigaku X-Stream low temperature device (Tokyo, Japan). Indexing [94], data reduction and correction for Lorenz, polarization, and absorption were performed using CrystalClear (Rigaku) software [95]. The structure was solved by direct methods using SHELXS [96] and refined by the least squares method on F^2^ using SHELXL [93]. See Table S1, Supplementary Information for collection and refinement details. All non-hydrogen atoms were refined with anisotropic displacement parameters. All C-bound hydrogen atoms were refined isotropic on calculated positions using a riding model with their *U*_iso_ values constrained to 1.5 times the *U*_eq_ of their pivot atoms for terminal sp^3^ carbon atoms and 1.2 times for all other carbon atoms. Crystallographic data for the structures reported in this paper have been deposited with the Cambridge Crystallographic Data Centre [97]. CCDC depositions 2452665, 2452666, 2452667, 2452668, and 2452669 contain the supplementary crystallographic data for this paper. These data can be obtained free of charge from the Cambridge Crystallographic Data Centre via www.ccdc.cam.ac.uk/structures, (accessed on 29 August 2025).

### 3.4. 3D Protein–Ligand Modelling

#### 3.4.1. Structure Preparation

The protein structure was prepared using the MOE 2022.02 (MOE, 2022) Quick Prep option [98] with the default Amber10:EHT force field in order to consider explicit hydrogen atoms, tautomeric states, and resolve the breaks Ser38-Asp46, Arg278-Ala285, Asp438-Glu448, and Thr439-Ala455 in protein structure prior to conducting restrained all-atom molecular mechanics minimisation and electrostatics calculations.

#### 3.4.2. Molecular Docking

To model the interactions between **CA-4**, the most potent compound **12l,** as well as **12s** and the tubulin protein, molecular docking was performed using OpenEye’s FRED v4.3.1.0 [89] software, which requires a set of low-energy conformations for each compound. One hundred conformations were generated by OMEGA v5.1.0.0 (https://www.eyesopen.com/omega, accessed on 29 August 2025) [99] for each compound. The active site for docking was prepared using the Make Receptor (OEDOCKING 4.3.2.1.) tool [100]. A box of the dimensions 19.30 Å × 21.26 Å × 17.90 Å was created around the co-crystallised ligand (CN2) present in 1Z2B. The poses generated after docking were sorted by their FRED Chemgauss4 score from best to worst. The top poses for each compound were selected to perform the molecular dynamics simulations.

#### 3.4.3. Molecular Dynamics (MD) Simulations

The molecular dynamics (MD) simulations were performed using GROMACS 2020.7 [88]. The protein chains were parameterised using the CHARMM36 [101] force field, whereas the compounds were parameterised using the CGenFF server [102]. The protein–ligand complex was centred in a cubic water box with an edge distance of 20.0 Å. The box was solvated using the TIP3P water model, and the system was then neutralised with 31 Na^+^ ions. The simulations were conducted within periodic boundary conditions. Energy minimisation was performed using the steepest descent for 5,000,000 steps. The minimised system was then equilibrated with a constant number of particles, volume, and temperature (NVT) ensemble, subjected to a 100-picoseconds (ps) run followed by another equilibration run with a constant number of particles, pressure (1 atm), and temperature (NPT) ensemble, at a constant temperature of 300 K for 100 ps. The MD production runs for each pose were set to 100 ns, and all restraints were removed. The GROMACS in-built tools were used to calculate the number of hydrogen bonds, root-mean-square deviations (RMSDs) between the chains as well as the compounds, and to perform a clustering analysis to output the most representative frame from the MD simulation. The output files were visualised using XMGRACE software [103], the most representative frame was structurally overlaid on the predicted docked pose with MOE, and the distance between the overlaid compounds was calculated considering the centre of mass (COM) using PyMOL (PyMOL v3.0) [104]. The graphs were generated using in-house Python scripts.

## 4. Conclusions

The four-membered *β*-lactam ring is a well-recognised pharmacophore, which demonstrates diverse biological activities including enzyme inhibition, antibacterial, antifungal, and antitubercular properties. We previously reported the synthesis, antiproliferative activity, and tubulin-targeting effects of azetidine-2-ones, containing the characteristic 3,4,5-trimethoxyphenyl Ring A of the antimitotic stilbene combretastatin CA-4, together with chloro, aryl, vinyl, and hydroxyl substituents at C-3 of the heterocycle [32,33,34,58]. Additionally, investigations of azetidine-2-ones related in structure to CA-4 with antimitotic and anticancer activity have also reported [35,36,37], which highlight the potential of this class of heterocyclic compounds for additional investigations. In the present work, a further series of azetidine-2-ones were developed in which the 3,5-dimethoxyphenyl substituent at N-1 of the azetidine-2-one replaces the characteristic 3,4,5-trimethoxyphenyl Ring A of CA-4, with the objective of investigating the antiproliferative activity of the products in MCF-7 breast cancer cells and HT-29 chemoresistant colon cancer cells. Phenoxy, aryl, and hydroxyl substituents were studied at C-3 of the four-membered ring, together with 3-unsubstituted examples. These compounds were designed as potential antiproliferative microtubule-targeting agents. The structures of compounds **12i**, **12k**, **12o**, **12p**, and **12u** were determined by single crystal X-ray analysis and confirmed the *trans* stereochemistry of the *β*-lactam ring protons at C-3 and C-4 for compounds **12k** and **12u**.

The inclusion of the 3,5-dimethoxyphenyl substituent at N-1 resulted in azetidine-2-one products, with potent antiproliferative activity in MCF-7 human breast cancer cells for **12a** (25 nM), **12b** (45 nM), **12l** (7 nM), **12m** (23 nM), and **12n** (31 nM). Similarly potent activities in HT-29 colon cancer cells were obtained for **12l** (3 nM), **12o** (IC_50_ = 89 nM), and **12p** (IC_50_ = 78 nM). In the MCF-7 cell line, low levels of LDH were released (2–9%) at 10 μM for selected compounds, indicating low cytotoxicity.

We explored the effect of the removal of the Ring A *para*-methoxy substituent and decreased steric bulk on drug potency and binding site interactions. By performing molecular docking and MD simulations, we demonstrated that the major contribution to hydrogen bond formation with the tubulin protein in **12s** is made by the 3-methoxy substituent, (42%) whereas for **12l**, the major contribution is made by 5-methoxy (12%) throughout the MD run. We conclude that the central 4-methoxy in **12s** does not contribute significantly to the formation of hydrogen bonds with the tubulin protein backbone. We can thus correlate the antiproliferative activities of the compounds in MCF-7 and HT-29 cancer cells with the calculated docking scores and molecular dynamics results to provide a possible explanation for the similar antiproliferative activities observed for both 3,4,5-trimethoxyphenyl Ring A compound **12s** and 3,5-dimethoxypheny Ring A compound **12l**, and rationalise the potent activity of the 3,5-dimethoxypheny Ring A series of synthesised azetidine-2-ones.

*β*-Lactam compound **12l**, with predicted drug-like physiochemical characteristics, can be considered as an improved derivative of our previous lead molecule in the search for new anticancer therapies. Future preclinical studies will investigate the wider applications for these compounds as potential antiproliferative microtubule-targeting chemotherapeutic agents. The structural study of these compounds will facilitate the further design of more effective and diverse *β*-lactams for potential development in breast and chemoresistant colon cancer applications.

## Figures and Tables

**Table 1:** X-Ray crystallography data, ring angles, and torsions for compounds **12i**, **12k**, **12o**, **12p**, **12u**, and **CA-4**.

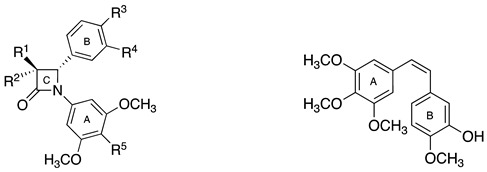							
		**12i** R^1^ = R^2^ = Cl, R^3^ = OCH_2_CH_3_, R^4^ = H, R^5^ = H **12k** R^1^ = OH, R^2^ = H, R^3^ = OCH_3_, R^4^ = H, R^5^ = H **12o** R^1^ = R^2^ = H, R^3^ = OCH_3_, R^4^ = H, R^5^ = H **12p** R^1^ = R^2^ = H, R^3^ = OCH_2_CH_3_, R^4^ = H, R^5^ = H **12u** R^1^ = OH, R^2^ = H, R^3^ = OCH_3_, R^4^ = CH_3_, R^5^ = OCH_3_				**CA-4**	
**Compound**	**Ring Plane** **Normal** **AB Angle (°) ^a^**	**Ring Plane** **Normal** **BC Angle (°) ^a^**	**Ring Plane** **Normal** **AC Angle (°) ^a^**	**Ring A to Central** **Torsion (°) ^a,b^**	**Ring B to Central** **Torsion** **(°) ^a,c^**	**Ring AB** **Torsion (°) ^a,d^**	**Ring BC** **Torsion** **(°) ^a,e^**
12i	73.84(3)	72.30(4)	4.50(7)	4.75(10)	−159.42(11)	63.36(16)	−117.16(10)
12k	80.66(3)	76.92(4)	6.02(8)	−3.39(19)	−149.88(11)	−62.89(16)	120.74(11)
12o	76.14(5)	78.93(6)	7.23(10)	−1.9(2)	147.69(11)	−57.13(16)	130.58(12)
12p	89.98(3)	89.65(3)	7.700(8)	10.83(16)	−123.83(10)	62.53(11)	−127.59(10)
12u	77.81(3)	79.45(5)	5.25(11)	2.1(2)	−145.76(11)	60.46(16)	−116.87(12)
CA-4	53.0(1)	-	-		144.0(4)	−9.4(8)	

^a^ The numbers in parentheses refer to the second crystallography independent molecule determined in the asymmetric unit. Atom numbering for torsions: ^b^ C15-C14-N1-C2, C18-C13-N1-C2, C15-C14-N1-C2, C19-C14-N1-C2, C19-C14-N1-C2 and ^d^ C5-C4-C3-Cl1 (down), C5-C4-C3-O24 (down), C5-C4-C3-H3B (down), C5-C4-C3-H3A (down), C5-C4-C3-O27 (down) for **12i**, **12k**, **12o**, **12p**, and **12u**, respectively; and ^c^ C10-C5-C4-N1, C6-C5-C4-N1, C6-C5-C4-N1, C10-C5-C4-N1, C6-C5-C4-N1, C10-C9-C2-C1 and ^e^ C14-N1-C4-C5, C13-N1-C4-C5, C14-N1-C4-C5, C14-N1-C4-C5, C14-N1-C4-C5, C3-C1-C2-C9 for **12i**, **12k**, **12o**, **12p**, **12u**, and **CA-4**, respectively.

**Table 2:** Structural parameters for **12i**, **12k**, **12o**, **12p**, and **12u**.

	12i (Å)	12k (Å)	12o (Å)	12p (Å)	12u (Å)
**C=O**	1.2023(15)	1.2181(14)	1.2104(16)	1.2140(12)	1.2222(16)
**C-2/C-3**	1.5415(16)	1.5428(16)	1.5260(19)	1.5244(14)	1.5354(17)
**N1/C-4**	1.4859(15)	1.4860(14)	1.4874(16)	1.4910(12)	1.4951(15)
**C-2/N1**	1.3689(15)	1.3661(14)	1.3772(16)	1.3754(12)	1.3589(16)

## Data Availability

The original contributions presented in this study are included in the article/Supplementary Material. Further inquiries can be directed to the corresponding author(s).

## Supplementary Materials

The following supporting information can be downloaded at https://www.mdpi.com/article/10.3390/ph18091330/s1: Table S1: X-Ray crystallography: Collection and refinement data for compounds **12i**, **12k**, **12o**, **12p**, and **12s**; Table S2: Cytotoxicity of 3,5-dimethoxyphenyl ring A *β*-lactams in MCF-7 breast cancer cells; Tables S3–S9: Physicochemical properties, Lipophilicity descriptors, Water solubility estimations, Pharmacokinetics, Drug-likeness, Medicinal Chemistry descriptors, and Toxicity Prediction for compounds **12a**–**12t**; Figure S1: Structures of azetidin-2-ones **12i**, **12k**, **12o**, **12p**, and **12s** for crystallography; Figure S2: Bioavailability Radar for 3,4-diaryl-2-azetidinones **12b**, **12l**, **12e**, **12m**, **12o**, and **12p**; Figure S3: The BOILED-Egg evaluation of passive gastrointestinal absorption (HIA) and brain penetration (BBB) of 3,4-diarylazetidin-2-ones **12b**, **12c**, **12l**, **12n**, **12o**, and **12p**; Figures S4–S49: ^1^H-NMR and ^13^C NMR spectra; Figures S50–S54: Hirshfeld surface analysis of 3,4-diarylazetidin-2-ones **12i**, **12k**, **12o 12p**, and **12u**.

## Abbreviations

ADC	Antibody–drug conjugate
ADME	Absorption, distribution, metabolism, and excretion
BBB	Blood–brain barrier
CA-4	Combretastatin A-4
CBSI	Colchicine binding site inhibitor
CCDC	Cambridge Crystallographic Data Centre
CDK	Cyclin-dependent kinase
CRC	Colorectal cancer
CYP2D6	Cytochrome P450 2D6
DAMA-colchicine	N-deacetyl-N-(2-mercaptoacetyl)-colchicine
DCM	Dichloromethane
DMEM	Dulbecco’s modified Eagle’s medium
ECACC	European Collection of Animal Cell Cultures
ESI	Electrospray Ionisation
EGF	Endothelial growth factor
ER	Estrogen receptor
FBS	Foetal bovine serum
GI	Gastrointestinal
HER2	Human epidermal growth factor receptor 2
HPLC	High Performance Liquid Chromatography
HRMS	High Resolution Mass Spectrometry
HR	Hormone Receptor
IC	Inhibitory concentration
IR	Infrared
LDH	Lactate dehydrogenase
mCRC	Metastatic colorectal cancer
MD	Molecular Dynamics
NMR	Nuclear Magnetic Resonance
PAINS	Pan-Assay Interference Compounds
PARP	Poly(adenosine diphosphate–ribose) polymerase
PD-1	Programmed cell death protein 1
P-gp	P-glycoprotein
PR	Progesterone receptor
RNS	Reactive nitrogen species
ROS	Reactive oxygen species
SAR	Structure–activity relationship
SERD	Selective estrogen receptor degrader
SERM	Selective estrogen receptor modulator
SIRT1	Sirtuin 1
TBNC	Triple-negative breast cancer
TEA	Triethylamine
TLC	Thin layer chromatography
TMS	Tetramethylsilane
TPSA	Topological Polar Surface Area
UGT	UDP-glucuronosyltransferase
VEGF	Vascular endothelial growth factor
