# Supplementary material for: Synthesis, Computational Studies, and Structural Analysis of 1-(3,5-Dimethoxyphenyl)azetidin-2-ones with Antiproliferative Activity in Breast Cancer and Chemoresistant Colon Cancer
Source: Pharmaceuticals (Basel). 2025 Sep 5;18(9):1330. doi: 10.3390/ph18091330 (PMC12472776; doi:10.3390/ph18091330)
Supplement: Supplementary file 1 [file pharmaceuticals-18-01330-s001.zip › pharmaceuticals-3774371-supplementary.pdf]

Supplementary Information

Synthesis, computational studies and structural analysis of 1-(3,5-dimethoxyphenyl)azetidin-2-ones with antiproliferative activity in breast cancer and chemoresistant colon cancer

Azizah M. Malebari[1], Shubhangi Kandwal[2,3,4], Abdirahman Ali[2,4], Darren Fayne[2,4], Brendan Twamley[5], Daniela M. Zisterer[6] and Mary J Meegan[7*]

[1] Department of Pharmaceutical Chemistry, College of Pharmacy, King Abdulaziz University, Jeddah, 21589, Saudi Arabia

[2] Molecular Design Group, School of Chemical Sciences, Dublin City University, Glasnevin, D09 V209 Dublin, Ireland

[3] Molecular Design Group, School of Biochemistry and Immunology, Trinity Biomedical Sciences Institute, Trinity College Dublin, 152-160 Pearse St, Dublin 2, D02 R590

[4] DCU Life Sciences Institute, Dublin City University, Glasnevin, D09 V209 Dublin, Ireland

[5] School of Chemistry, Trinity College Dublin, D02 P3X2 Dublin, Ireland

[6] Trinity Biomedical Sciences Institute, School of Biochemistry and Immunology, Trinity College Dublin, 152-160 Pearse Street, Dublin 2, D02 R590, Ireland

[7] School of Pharmacy and Pharmaceutical Sciences, Panoz Institute, Trinity College Dublin, D02 PN40 Dublin, Ireland

*Correspondence: mmeegan@tcd.ie

Table S1: X-Ray crystallography: Collection and refinement data for compounds **12i**, **12k**, **12o**, **12p** and **12u**

Table S2: Cytotoxicity of 3,5-dimethoxyphenyl ring A β-lactams in MCF-7 breast cancer cells

Table S3: Physicochemical properties of 3,4-diarylazetidin-2-ones **12a-12u** and CA-4

Table S4: Lipophilicity descriptors of 3,4-diarylazetidin-2-ones **12a-12u** and CA-4

Table S5: Water solubility estimations for 3,4-diarylazetidin-2-ones **12a-12u** and CA-4

Table S6: Pharmacokinetics for 3,4-diarylazetidin-2-ones **12a-12u** and CA-4

Table S7: Drug-likeness for 3,4-diarylazetidin-2-ones **12a-12u** and CA-4

Table S8: Medicinal Chemistry descriptors for 3,4-diarylazetidin-2-ones **12a-12u** and CA-4

Table S9: Toxicity Prediction for 3,4-diarylazetidin-2-ones **12a-12u** and CA-4

Figure S1: Structures of azetidin-2-ones **12i, 12k, 12o, 12p** and **12u** for crystallography.

Figure S2: Bioavailability Radar for 3,4-diarylazetidin-2-ones **12b, 12l, 12e, 12m, 12o** and **12p**

Figure S3: The BOILED-Egg evaluation of passive gastrointestinal absorption (HIA) and brain penetration (BBB) for 3,4-diarylazetidin-2-ones **12b, 12c, 12l, 12n, 12o** and **12p**

Figure S4-S49: ^{1}H-NMR and ^{13}C NMR spectra

Figure S50-S54: Hirshfeld surface analysis of 3,4-diarylazetidin-2-ones **12i, 12k, 12o 12p** and **12u**

Table S1. Collection and refinement data for compounds 12i, 12k, 12o, 12p and 12u

Compound No.	**12i**	**12k**	**12o**	**12p**	**12u**
CCDC number	**2452665**	**2452666**	**2452667**	**2452668**	**2452669**
Empirical formula	$C_{19}H_{19}Cl_2NO_4$	$C_{18}H_{19}NO_5$	$C_{18}H_{19}NO_4$	$C_{19}H_{21}NO_4$	$C_{20}H_{23}NO_6$
Formula weight	396.25	329.34	313.34	327.37	373.39
Temperature [K]	100(2)	100(2)	93(2)	100(2)	100(2)
Crystal system	triclinic	monoclinic	monoclinic	monoclinic	monoclinic
Space group (number)	$P\overline{1}$ (2)	$P2_1/n$ (14)	$P2_1/n$ (14)	$P2_1/n$ (14)	$P2_1/c$ (14)
a [Å]	9.3229(3)	9.8263(5)	6.2914(13)	4.9322(3)	11.8506(5)
b [Å]	9.9977(3)	10.9867(6)	23.683(5)	16.8720(9)	6.7029(3)
c [Å]	11.5014(4)	15.3860(8)	11.023(2)	20.0509(11)	23.2328(10)
α [°]	86.9426(14)	90	90	90	90
β [°]	70.4870(13)	105.4940(10)	105.92(3)	93.3589(17)	94.8910(10)
γ [°]	68.7191(12)	90	90	90	90
Volume [$Å^3$]	938.73(5)	1600.69(15)	1579.5(6)	1665.69(16)	1838.74(14)
Z	2	4	4	4	4
ρ_{calc} [gcm^{-3}]	1.402	1.367	1.318	1.305	1.349
μ [mm^{-1}]	0.370	0.100	0.093	0.092	0.829
F(000)	412	696	664	696	792
Crystal size [mm^3]	0.190×0.130×0.110	0.280×0.170×0.040	0.26×0.11×0.11	0.320×0.140×0.040	0.23×0.21×0.14
Crystal colour	colorless	colorless	clear colourless	colorless	clear colourless
Crystal shape	fragment	plate	block	fragment	block
Radiation	MoK_α (λ=0.71073 Å)	MoK_α (λ=0.71073 Å)	MoK_α (λ=0.71073 Å)	MoK_α (λ=0.71073 Å)	CuK_α (λ=1.54178 Å)
2Θ range [°]	3.77 to 57.74 (0.74 Å)	4.44 to 60.32 (0.71 Å)	4.21 to 54.97 (0.77 Å)	3.16 to 58.32 (0.73 Å)	7.49 to 140.04 (0.82 Å)
Reflections collected	69851	68047	26860	64771	21675
Independent reflections	4935<br>R_{int} = 0.0282<br>R_{sigma} = 0.0135	4737<br>R_{int} = 0.0457<br>R_{sigma} = 0.0199	3612<br>R_{int} = 0.0256<br>R_{sigma} = 0.0144	4485<br>R_{int} = 0.0285<br>R_{sigma} = 0.0127	3463<br>R_{int} = 0.0319<br>R_{sigma} = 0.0204
Data / Restraints / Parameters	4935/0/238	4737/0/217	3612/0/211	4485/0/220	3463/0/252
Goodness-of-fit on F^2	1.024	1.025	1.104	1.016	1.050
Final *R* indexes [$I \geq 2\sigma(I)$]	R_1 = 0.0282<br>wR_2 = 0.0726	R_1 = 0.0413<br>wR_2 = 0.0988	R_1 = 0.0456<br>wR_2 = 0.1064	R_1 = 0.0351<br>wR_2 = 0.0873	R_1 = 0.0416<br>wR_2 = 0.1135
Final *R* indexes [all data]	R_1 = 0.0376<br>wR_2 = 0.0794	R_1 = 0.0602<br>wR_2 = 0.1091	R_1 = 0.0476<br>wR_2 = 0.1078	R_1 = 0.0447<br>wR_2 = 0.0942	R_1 = 0.0420<br>wR_2 = 0.1141
Largest peak/hole [$eÅ^{-3}$]	0.41/-0.22	0.45/-0.23	0.26/-0.20	0.35/-0.23	0.26/-0.26

Table S2: Cytotoxicity of 3,5-dimethoxyphenyl ring A β-lactams in MCF-7 breast cancer cells[a]

Compound	% cell death[a]
**12a**	3.3
**12b**	2.5
**12c**	2.4
**12f**	8.9
**12j**[b]	3.2
**CA-4**	1.1
**Control (Lysis solution)**	100

[a] MCF-7 breast cancer cells were treated with β-lactams **12a**, **12b**, **12c**, **12f**, **12j** and CA-4 at 10 μM for 24 h. LDH release was determined with Promega LDH assay kit (G1780) [1] and % cell death at 10 μM is shown. [b][2]

Table S3: Physicochemical properties of 3,4-diarylazetidin-2-ones 12a-12u and CA-4[a]

	Formula	MW	#Heavy atoms	#Aromatic heavy atoms	Fraction Csp^3	#Rotatable bonds	#H-bond acceptors	#H-bond donors	MR	TPSA
**Code**										
**CA-4**	$C_{18}H_{20}O_5$	316.35	23	12	0.22	6	5	1	89.80	57.15
**12a**	$C_{24}H_{23}NO_4$	389.44	29	18	0.21	6	4	0	114.93	48.00
**12b**	$C_{25}H_{25}NO_4$	403.47	30	18	0.24	7	4	0	119.73	48.00
**12c**	$C_{24}H_{23}NO_3S$	405.51	29	18	0.21	6	3	0	120.16	64.07
**12d**	$C_{24}H_{23}NO_5$	405.44	30	18	0.21	7	5	0	116.45	57.23
**12e**	$C_{25}H_{25}NO_5$	419.47	31	18	0.24	8	5	0	121.26	57.23
**12f**	$C_{18}H_{18}ClNO_4$	347.79	24	12	0.28	5	4	0	95.24	48.00
**12g**	$C_{19}H_{20}ClNO_4$	361.82	25	12	0.32	6	4	0	100.04	48.00
**12h**	$C_{18}H_{17}Cl_2NO_4$	382.24	25	12	0.28	5	4	0	100.07	48.00
**12i**	$C_{19}H_{19}Cl_2NO_4$	396.26	26	12	0.32	6	4	0	104.88	48.00
**12j**	$C_{20}H_{21}NO_4$	339.39	25	12	0.25	6	4	0	99.58	48.00
**12k**	$C_{18}H_{19}NO_5$	329.35	24	12	0.28	5	5	1	91.60	68.23
**12l**	$C_{19}H_{21}NO_5$	343.37	25	12	0.32	6	5	1	96.41	68.23
**12m**	$C_{18}H_{19}NO_4S$	345.41	24	12	0.28	5	4	1	96.83	84.30
**12n**	$C_{19}H_{21}NO_4S$	359.44	25	12	0.32	6	4	1	101.64	84.30
**12o**	$C_{18}H_{19}NO_4$	313.35	23	12	0.28	5	4	0	90.44	48.00
**12p**	$C_{19}H_{21}NO_4$	327.37	24	12	0.32	6	4	0	95.25	48.00
**12q**	$C_{18}H_{19}NO_3S$	329.41	23	12	0.28	5	3	0	95.67	64.07
**12r**	$C_{19}H_{21}NO_3S$	343.44	24	12	0.32	6	3	0	100.48	64.07
**12s**	$C_{18}H_{19}NO_5$	329.35	24	12	0.28	5	5	1	91.60	68.23
**12t**	$C_{20}H_{23}NO_6$	373.40	27	12	0.35	7	6	1	102.90	77.46
**12u**	$C_{20}H_{23}NO_6$	373.40	27	12	0.35	6	6	1	103.06	77.46

[a]SwissADME http://www.swissadme.ch;

Table S4: Lipophilicity descriptors of 3,4-diarylazetidin-2-ones 12a-12u and CA-4[a].

	iLOGP	XLOGP3	WLOGP	MLOGP	Silicos-IT Log P	Consensus Log P[b]	Molecular Volume[c] (Å^3)
**Code**							
**CA-4**	3.42	3.72	3.38	2.10	3.73	3.27	293.07
**12a**	3.96	4.13	3.88	3.72	4.15	3.97	258.08
**12b**	4.22	4.49	4.27	3.93	4.54	4.29	374.88
**12c**	4.01	4.67	4.59	4.55	4.71	4.51	369.06
**12d**	3.82	4.23	3.54	3.17	3.68	3.69	383.87
**12e**	4.11	4.60	3.93	3.38	4.08	4.02	367.22
**12f**	3.34	3.15	2.70	2.85	3.19	3.05	300.21
**12g**	3.64	3.52	3.09	3.08	3.58	3.38	317.01
**12h**	3.61	3.66	3.27	3.08	3.81	3.49	313.42
**12i**	3.70	4.03	3.66	3.30	4.20	3.78	330.22
**12j**	3.61	3.28	2.90	3.00	3.45	3.25	314.40
**12k**	2.91	1.88	1.46	1.81	2.07	2.02	294.69
**12l**	3.43	2.25	1.85	2.04	2.46	2.40	311.49
**12m**	2.87	2.42	2.17	2.62	2.63	2.54	303.83
**12n**	3.50	2.79	2.56	2.85	3.02	2.94	320.63
**12o**	3.26	2.43	2.49	2.62	2.97	2.75	286.64
**12p**	3.68	2.80	2.88	2.85	3.35	3.11	303.45
**12q**	3.19	2.97	3.20	3.45	3.53	3.27	295.79
**12r**	3.50	3.34	3.59	3.68	3.91	3.60	312.59
**12s**	2.91	1.88	1.46	1.81	2.07	2.02	320.23
**12t**	3.23	2.22	1.85	1.73	2.53	2.31	337.04
**12u**	3.15	2.21	1.77	1.73	2.66	2.31	336.80

[a]SwissADME http://www.swissadme.ch; [b]Average of all five predictions; [c] Molinspiration Cheminformatics free web services, https://www.molinspiration.com, Slovensky Grob, Slovakia. Method for calculation of molecule volume developed at Molinspiration is based on group contributions. These have been obtained by fitting sum of fragment contributions to "real" 3D volume for a training set of about twelve thousand, mostly drug-like molecules. 3D molecular geometries for a training set were fully optimized by the semiempirical AM1 method

Table S5: Water solubility estimations for 3,4-diarylazetidin-2-ones 12a-u and CA-4[a, b]

	ESOL Log S	ESOL Solubility (mg/ml)	ESOL Solubility (mol/l)	ESOL Class[c]	Ali Log S	Ali Solubility (mg/ml)	Ali Solubility (mol/l)	Ali Class[c]	Silicos-IT LogSw	Silicos-IT Solubility (mg/ml)	Silicos-IT Solubility (mol/l)	Silicos-IT class[c]
**Code**												
**CA-4**	-4.14	0.02320	0.000073	Moderately soluble	-4.61	0.007740	0.000024	Moderately soluble	-4.92	0.003790	1.200000e-05	Moderately soluble
**12a**	-4.92	0.00468	0.000012	Moderately soluble	-4.84	0.005570	0.000014	Moderately soluble	-7.34	0.000018	4.580000e-08	Poorly soluble
**12b**	-5.15	0.00284	0.000007	Moderately soluble	-5.22	0.002440	0.000006	Moderately soluble	-7.73	0.000007	1.850000e-08	Poorly soluble
**12c**	-5.36	0.00177	0.000004	Moderately soluble	-5.74	0.000733	0.000002	Moderately soluble	-7.70	0.000008	2.010000e-08	Poorly soluble
**12d**	-5.00	0.00405	0.000010	Moderately soluble	-5.14	0.002920	0.000007	Moderately soluble	-7.07	0.000035	8.590000e-08	Poorly soluble
**12e**	-5.24	0.00241	0.000006	Moderately soluble	-5.53	0.001250	0.000003	Moderately soluble	-7.46	0.000015	3.480000e-08	Poorly soluble
**12f**	-4.02	0.03320	0.000095	Moderately soluble	-3.83	0.051700	0.000149	Soluble	-5.47	0.001180	3.390000e-06	Moderately soluble
**12g**	-4.26	0.01990	0.000055	Moderately soluble	-4.21	0.022200	0.000061	Moderately soluble	-5.87	0.000494	1.360000e-06	Moderately soluble
**12h**	-4.54	0.01100	0.000029	Moderately soluble	-4.36	0.016800	0.000044	Moderately soluble	-6.28	0.000201	5.250000e-07	Poorly soluble
**12i**	-4.78	0.00656	0.000017	Moderately soluble	-4.74	0.007190	0.000018	Moderately soluble	-6.67	0.000084	2.120000e-07	Poorly soluble
**12j**	-3.97	0.03640	0.000107	Soluble	-3.96	0.037000	0.000109	Soluble	-5.30	0.001680	4.950000e-06	Moderately soluble
**12k**	-3.11	0.25800	0.000783	Soluble	-2.93	0.383000	0.001160	Soluble	-4.29	0.016900	5.120000e-05	Moderately soluble
**12l**	-3.35	0.15500	0.000451	Soluble	-3.32	0.165000	0.000480	Soluble	-4.69	0.007060	2.060000e-05	Moderately soluble
**12m**	-3.55	0.09820	0.000284	Soluble	-3.83	0.050800	0.000147	Soluble	-4.65	0.007720	2.230000e-05	Moderately soluble
**12n**	-3.79	0.05890	0.000164	Soluble	-4.22	0.021800	0.000061	Moderately soluble	-5.05	0.003230	8.990000e-06	Moderately soluble
**12o**	-3.37	0.13400	0.000427	Soluble	-3.08	0.260000	0.000830	Soluble	-5.11	0.002460	7.850000e-06	Moderately soluble
**12p**	-3.61	0.08080	0.000247	Soluble	-3.46	0.112000	0.000343	Soluble	-5.50	0.001030	3.150000e-06	Moderately soluble
**12q**	-3.81	0.05110	0.000155	Soluble	-3.98	0.034600	0.000105	Soluble	-5.47	0.001130	3.420000e-06	Moderately soluble
**12r**	-4.05	0.03080	0.000090	Moderately soluble	-4.36	0.014900	0.000043	Moderately soluble	-5.86	0.000472	1.370000e-06	Moderately soluble
**12s**	-3.11	0.258	0.000783	Soluble	-2.93	0.383000	0.00116	Soluble	-4.29	0.01690	5.120000e-05	Moderately soluble
**12t**	-3.42	0.14200	0.000380	Soluble	-3.48	0.123000	0.000330	Soluble	-4.79	0.006010	1.610000e-05	Moderately soluble
**12u**	-3.48	0.12400	0.000331	Soluble	-3.47	0.126000	0.000338	Soluble	-4.78	0.006250	1.670000e-05	Moderately soluble

[a]SwissADME http://www.swissadme.ch; [b]Topological method to predict Water Solubility included in SwissADME using an implementation of the ESOL model; [c]Solubility class: Log S scale: Insoluble < -10 < Poorly < -6 < Moderately < -4 < Soluble < -2 Very < 0 < Highly

Table S6: Pharmacokinetics for 3,4-diarylazetidin-2-ones 12a-12u and CA-4[a]

	GI absorption[b]	BBB permeant[b]	Pgp substrate	CYP1A2 inhibitor	CYP2C19 inhibitor	CYP2C9 inhibitor	CYP2D6 inhibitor	CYP3A4 inhibitor	log Kp (cm/s)[c]
**Code**									
**CA-4**	High	Yes	No	Yes	Yes	Yes	Yes	Yes	-5.59
**12a**	High	Yes	No	No	Yes	Yes	Yes	Yes	-5.74
**12b**	High	Yes	Yes	No	Yes	Yes	Yes	Yes	-5.57
**12c**	High	Yes	Yes	No	Yes	Yes	Yes	Yes	-5.46
**12d**	High	Yes	No	No	Yes	Yes	Yes	Yes	-5.77
**12e**	High	Yes	No	No	Yes	Yes	Yes	Yes	-5.59
**12f**	High	Yes	No	No	Yes	Yes	Yes	Yes	-6.19
**12g**	High	Yes	No	No	Yes	Yes	Yes	Yes	-6.01
**12h**	High	Yes	No	No	Yes	Yes	Yes	Yes	-6.03
**12i**	High	Yes	No	No	Yes	Yes	Yes	Yes	-5.86
**12j**	High	Yes	No	No	Yes	Yes	Yes	Yes	-6.04
**12k**	High	Yes	No	No	No	No	Yes	No	-6.97
**12l**	High	Yes	No	No	No	No	Yes	No	-6.80
**12m**	High	No	No	No	Yes	No	Yes	No	-6.69
**12n**	High	No	No	No	Yes	Yes	Yes	Yes	-6.51
**12o**	High	Yes	No	No	Yes	Yes	Yes	Yes	-6.49
**12p**	High	Yes	No	No	Yes	Yes	Yes	Yes	-6.31
**12q**	High	Yes	No	No	Yes	Yes	Yes	Yes	-6.20
**12r**	High	Yes	No	No	Yes	Yes	Yes	Yes	-6.02
**12s**	High	Yes	No	No	No	No	Yes	No	-6.97
**12t**	High	No	Yes	No	No	No	Yes	No	-7.00
**12u**	High	No	Yes	No	No	No	Yes	No	-7.01

[a] SwissADME: a free web tool to evaluate pharmacokinetics, drug-likeness and medicinal chemistry friendliness of small molecules http://www.swissadme.ch; [b]According to the yolk of the BOILED-Egg; [c]Skin permeation

Table S7: Drug-likeness for 3,4-diarylazetidin-2-ones 12a-12u and CA-4[a,d]

	Lipinski #violations[b]	Ghose #violations[b]	Veber #violations[b]	Egan #violations[b]	Muegge #violations[b]	Bioavailability Score[c]
**Code**						
**CA-4**	0	0	0	0	0	0.55
**12a**	0	0	0	0	0	0.55
**12b**	0	0	0	0	0	0.55
**12c**	1	0	0	0	0	0.55
**12d**	0	0	0	0	0	0.55
**12e**	0	0	0	0	0	0.55
**12f**	0	0	0	0	0	0.55
**12g**	0	0	0	0	0	0.55
**12h**	0	0	0	0	0	0.55
**12i**	0	0	0	0	0	0.55
**12j**	0	0	0	0	0	0.55
**12k**	0	0	0	0	0	0.55
**12l**	0	0	0	0	0	0.55
**12m**	0	0	0	0	0	0.55
**12n**	0	0	0	0	0	0.55
**12o**	0	0	0	0	0	0.55
**12p**	0	0	0	0	0	0.55
**12q**	0	0	0	0	0	0.55
**12r**	0	0	0	0	0	0.55
**12s**	0	0	0	0	0	0.55
**12t**	0	0	0	0	0	0.55
**12u**	0	0	0	0	0	0.55

[a]SwissADME http://www.swissadme.ch; [b]Additional druglikeness assessment methods including Ghose, Veber and Muegge filters together with medicinal chemistry structure alert assessments PAINS (pan assay interference compounds) and Brenk filters were used to identify compounds which are potentially toxic, chemically reactive, metabolically unstable compounds or having poor pharmacokinetics [c]The bioavailability score is the parameter which predict the probability of more than 10% oral bioavailability in rat based on PSA (potential surface area) and Lipinski rule-of-five. This parameter falls on four classes of probabilities (11%, 17%, 56% or 85%) and allows to filter off the molecules with cell-permeability issues

Table S8: Medicinal Chemistry descriptors for 3,4-diarylazetidin-2-ones 12a-12u and CA-4[a]

	PAINS #alerts[b]	Brenk #alerts[c]	Leadlikeness #violations[d]	Synthetic Accessibility
**Code**				
**CA-4**	0	1	1	2.65
**12a**	0	0	2	3.39
**12b**	0	0	2	3.51
**12c**	0	0	2	3.42
**12d**	0	0	2	3.57
**12e**	0	0	3	3.68
**12f**	0	1	0	3.04
**12g**	0	1	2	3.15
**12h**	0	1	2	2.81
**12i**	0	1	2	2.92
**12j**	0	1	0	3.25
**12k**	0	0	0	2.99
**12l**	0	0	0	3.12
**12m**	0	0	0	3.04
**12n**	0	0	1	3.22
**12o**	0	0	0	2.59
**12p**	0	0	0	2.71
**12q**	0	0	0	2.65
**12r**	0	0	0	2.84
**12s**	0	0	0	2.99
**12t**	0	0	1	3.32
**12u**	0	0	1	3.34

[a]SwissADME http://www.swissadme.ch [b]Pan Assay Interference Structures: (PAINS); [c]Brenk Structural Alert e.g. Michael acceptor, more than 2 esters; nitro group, oxygen-nitrogen single bond, phthalimide, alkyl halide; [d]druglikeness assessment methods including Ghose, Veber and Muegge filters e.g. MW>350 together with medicinal chemistry structure alert assessments PAINS (pan assay interference compounds) and Brenk filters were used to identify compounds which are potentially toxic, chemically reactive, metabolically unstable compounds or having poor pharmacokinetics.

Table S9: Toxicity Prediction for 3,4-diarylazetidin-2-ones 12a-12u and CA-4[a]

Compound No.	AMES Toxicity	Max. tolerated dose (human) (log mg/kg/day)	Hepatotoxicity
**Code**			
**CA-4**	No	0.743	No
**12a**	No	0.309	Yes
**12b**	No	0.438	No
**12c**	Yes	0.238	No
**12d**	Yes	0.715	No
**12e**	Yes	0.753	No
**12f**	No	0.489	No
**12g**	Yes	0.544	No
**12h**	Yes	0.736	No
**12i**	Yes	0.776	No
**12j**	Yes	0.489	No
**12k**	Yes	0.605	No
**12l**	Yes	0.612	Yes
**12m**	Yes	0.384	No
**12n**	Yes	0.686	No
**12o**	No	0.625	No
**12p**	No	0.664	No
**12q**	No	0.606	No
**12r**	No	0.704	No
**12s**	Yes	0.107	No
**12t**	No	0.963	No
**12u**	Yes	0.481	No

[a]Predictions from the pkCSM (http://biosig.unimelb.edu.au/pkcsm/prediction) web interface

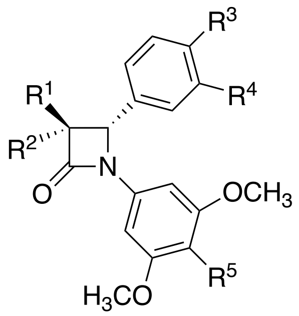

12i $R^1=R^2=Cl$, $R^3=OCH_2CH_3$, $R^4=H$, $R^5=H$
12k $R^1=$ OH, $R^2=H$, $R^3=OCH_3$, $R^4=H$, $R^5=H$
12o $R^1=R^2=H$, $R^3=OCH_3$, $R^4=H$, $R^5=H$
12p $R^1=R^2=H$, $R^3=OCH_2CH_3$, $R^4=H$, $R^5=H$
12u $R^1=OH$, $R^2=H$, $R^3=OCH_3$, $R^4=CH_3$, $R^5=OCH_3$

Figure S1: Structures of azetidin-2-ones **12i, 12k, 12o, 12p** and **12u** for crystallography **12b**

12e

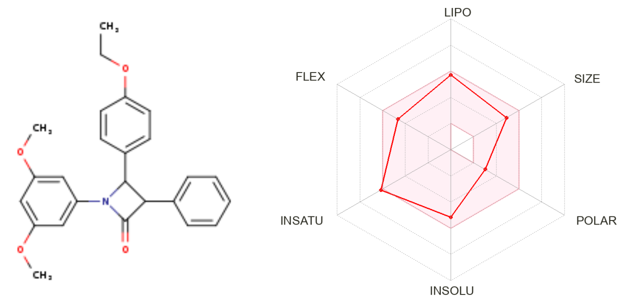

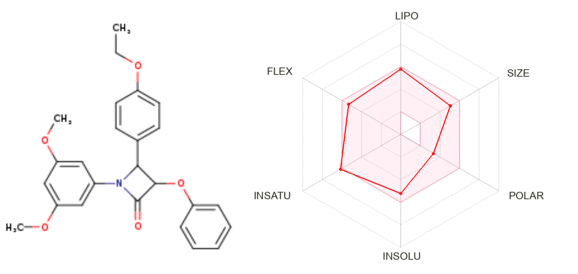

12l

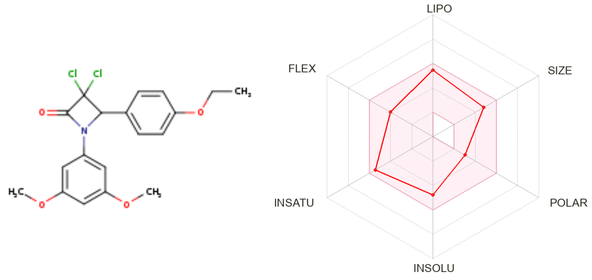

12m

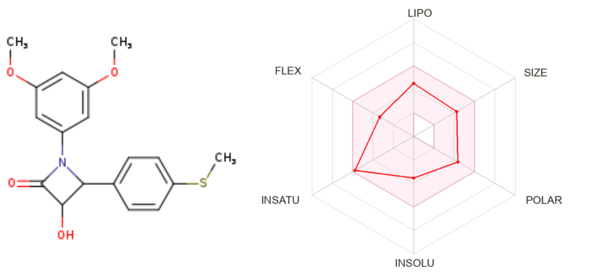

12o

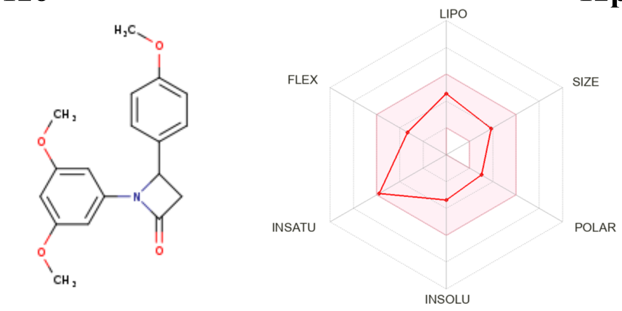

12p

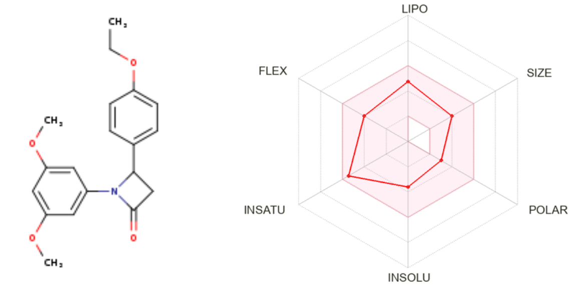

Figure S2: Bioavailability Radar for 3,4-diarylazetidin-2-ones **12b, 12l, 12e, 12m, 12o** and **12p**

The six physicochemical properties considered for analysis of drug-likeness in the SwissADME model are: lipophilicity (XLOGP3), size (MW), polarity (TPSA, topological polar surface area), solubility (logS), saturation (fsp^3) and flexibility (number of rotatable bonds). A physicochemical range on each axis is depicted as a pink area in which the radar plot of the molecule has to fall entirely to be considered drug-like. The pink area represents the optimal range for each properties (lipophilicity: XLOGP3 between −0.7 and +5.0, size: MW between 150 and 500 g/mol, polarity: TPSA between 20 and 130 Å^2, solubility: log S not higher than 6, saturation: fraction of carbons in the sp^3 hybridization not less than 0.25, and flexibility: no more than 9 rotatable bonds. (SwissADME http://www.swissadme.ch).

12b

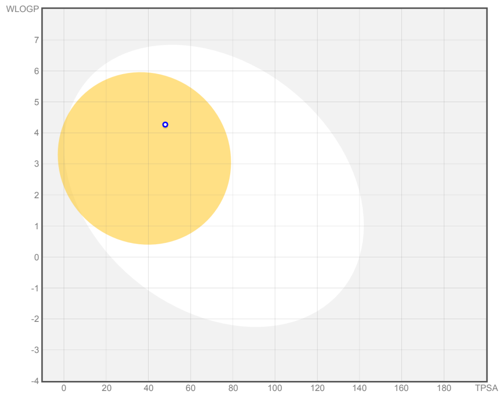

12e

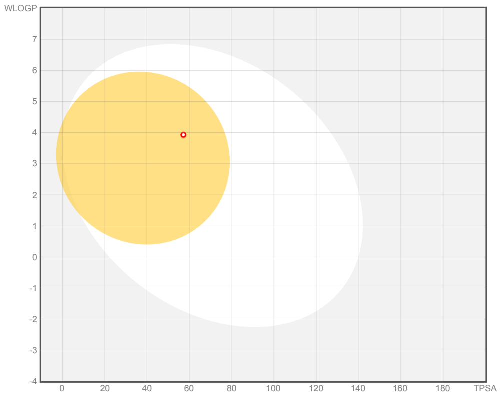

12l

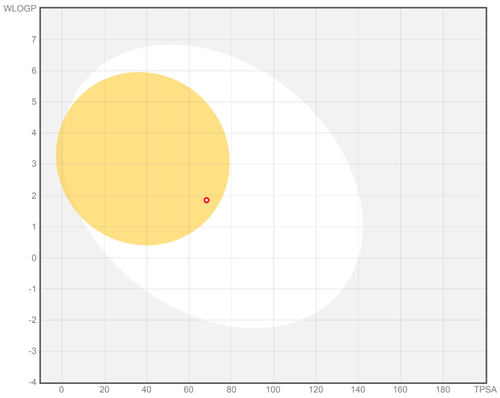

12n

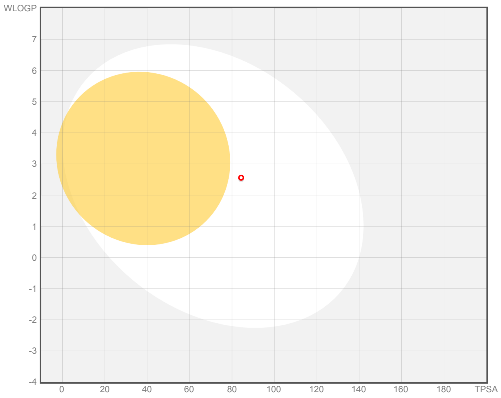

12o

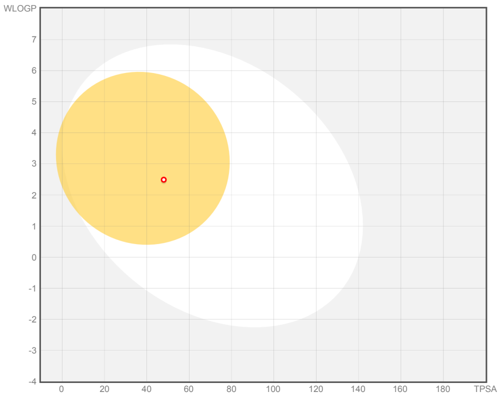

12p

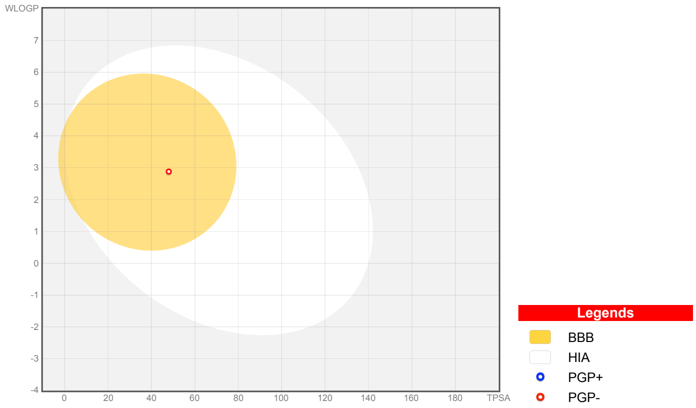

Figure S3: The Brain Or Intestinal EstimateD permeation method (BOILED-Egg) for evaluation of passive gastrointestinal absorption (HIA) and brain penetration (BBB) of 3,4-diarylazetidin-2-ones **12b, 12c, 12l, 12n, 12o** and **12p**

BOILED-Egg is a visual prediction model for the evaluation of passive gastrointestinal absorption (HIA) and blood-brain-barrier (BBB) penetration. The data for compounds **12b, 12c, 12l, 12n, 12o** and **12p** is visually analysed on the same 2D map with WLOGP and TPSA plot. (SwissADME http://www.swissadme.ch).

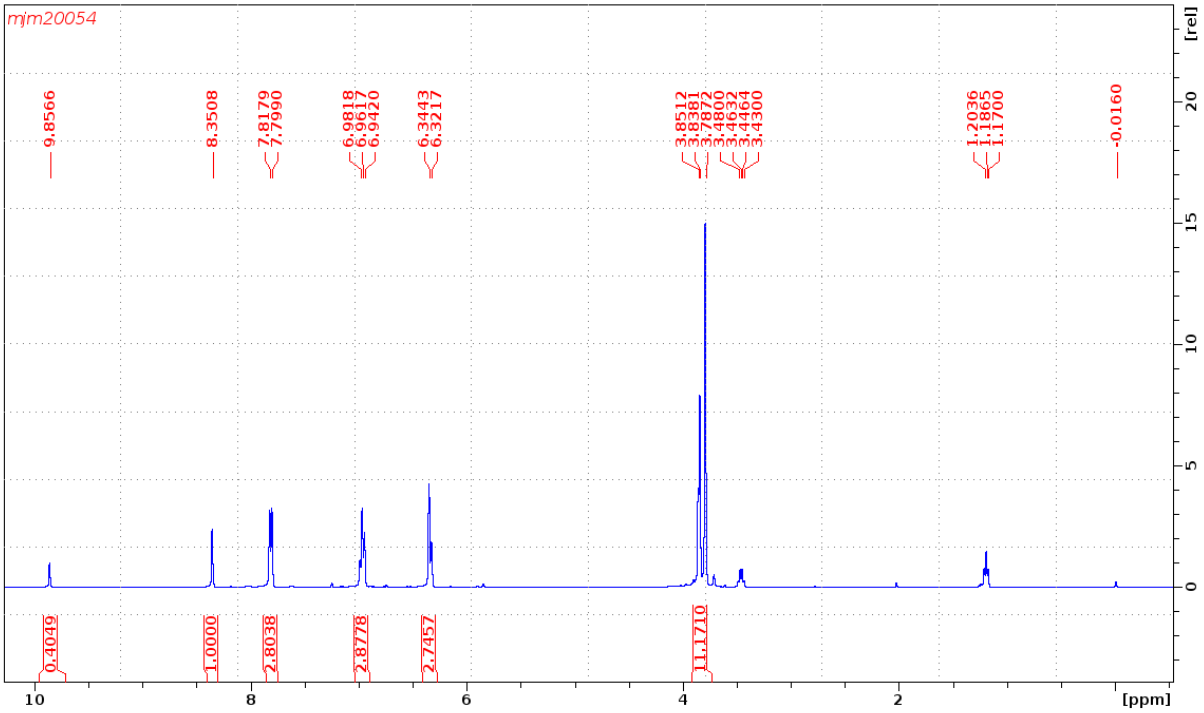

Figure S4: ^{1}H NMR Spectrum (*E*)-N-(3,5-Dimethoxyphenyl)-1-(4-methoxyphenyl)methanimine **(11a)**; [Diethyl ether δ 1.18(t), δ 3.46(q)]

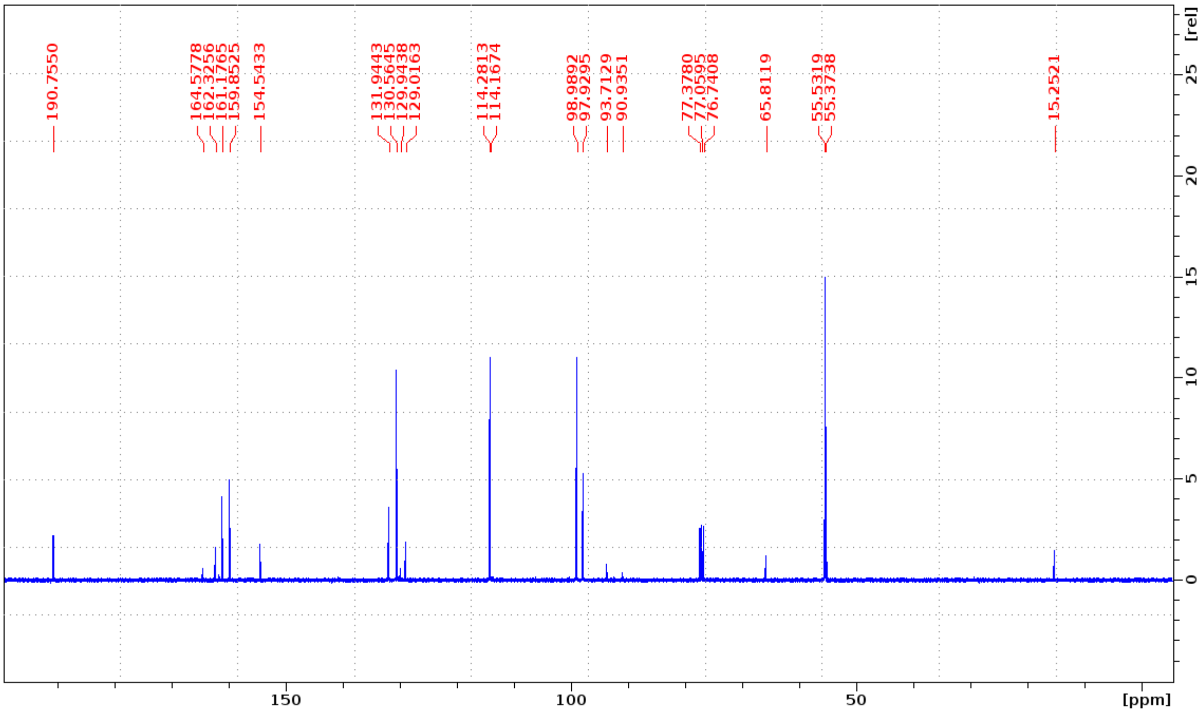

Figure S5: ^{13}C NMR Spectrum (*E*)-N-(3,5-Dimethoxyphenyl)-1-(4-methoxyphenyl)methanimine **(11a)**

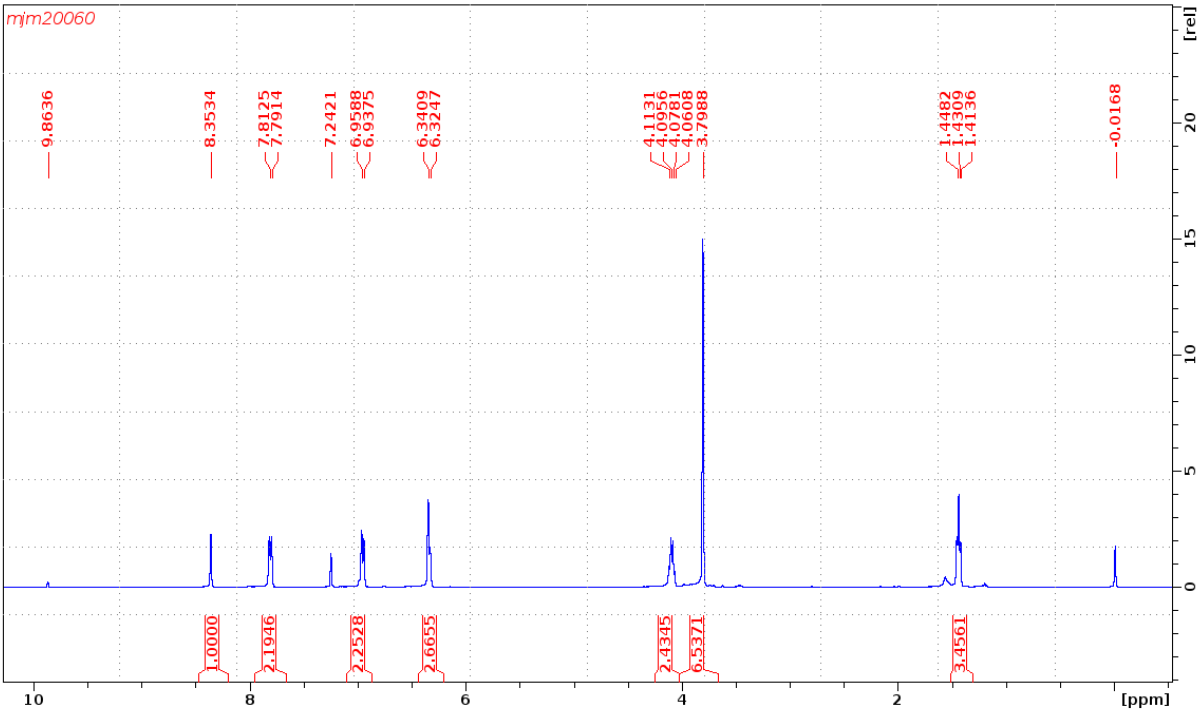

Figure S6: ¹H NMR Spectrum (*E*)-*N*-(3,5-Dimethoxyphenyl)-1-(4-ethoxyphenyl)methanimine **(11b)** (impurity H_2O at δ 1.55)

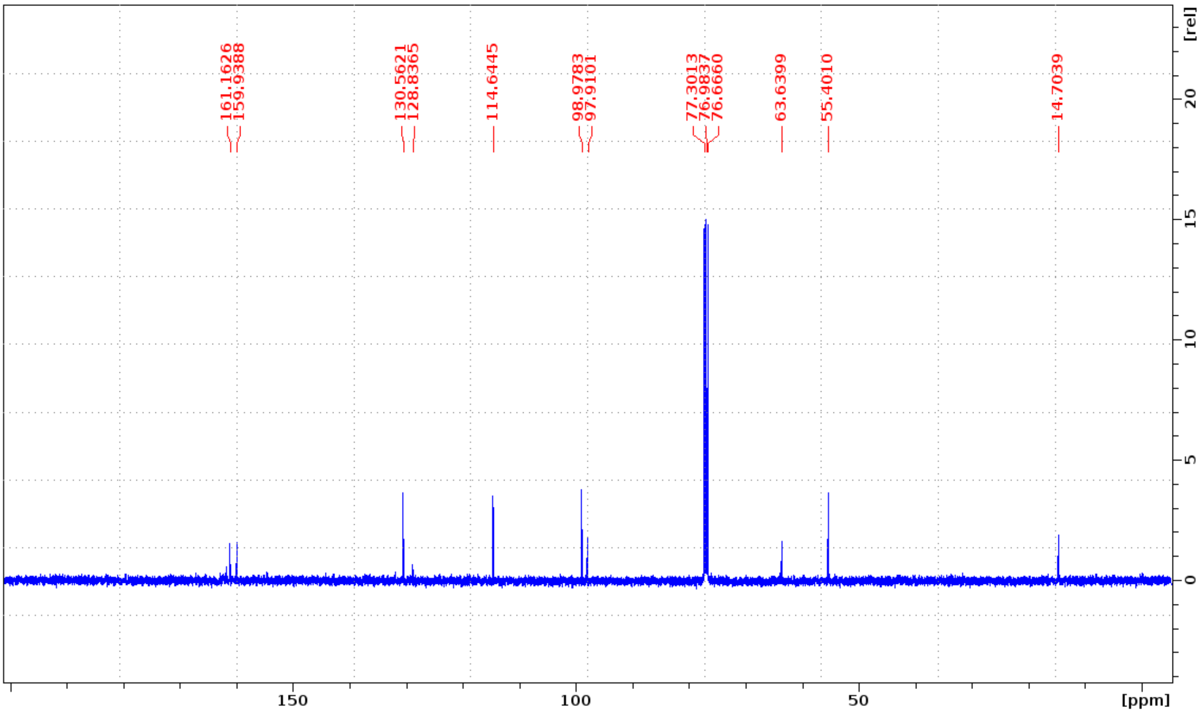

Figure S7: ¹³C NMR Spectrum (*E*)-*N*-(3,5-Dimethoxyphenyl)-1-(4-ethoxyphenyl)methanimine **(11b)**

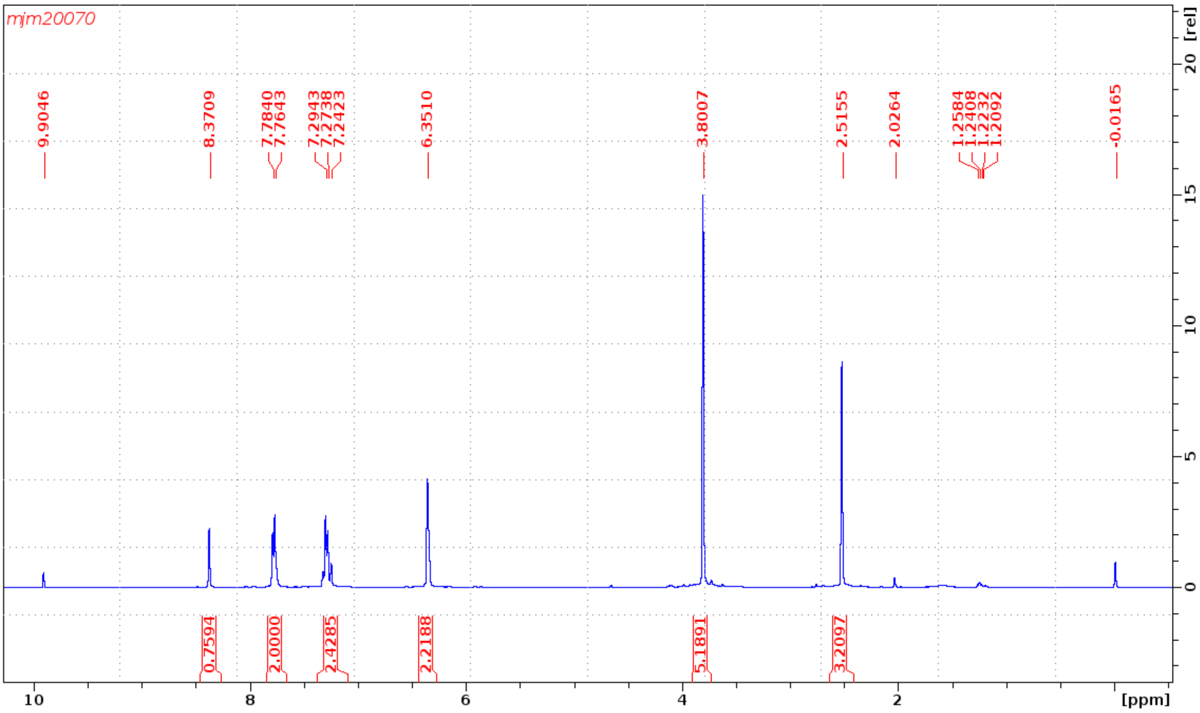

Figure S8: ^{1}H NMR Spectrum (*E*)-N-(3,5-Dimethoxyphenyl)-1-(4-(methylthio)phenyl)methanimine **(11c)** [Ethyl acetate impurity (δ 1.24, 2.02, 4.12), 4-methylthio)benzaldehyde (δ 9.90)].

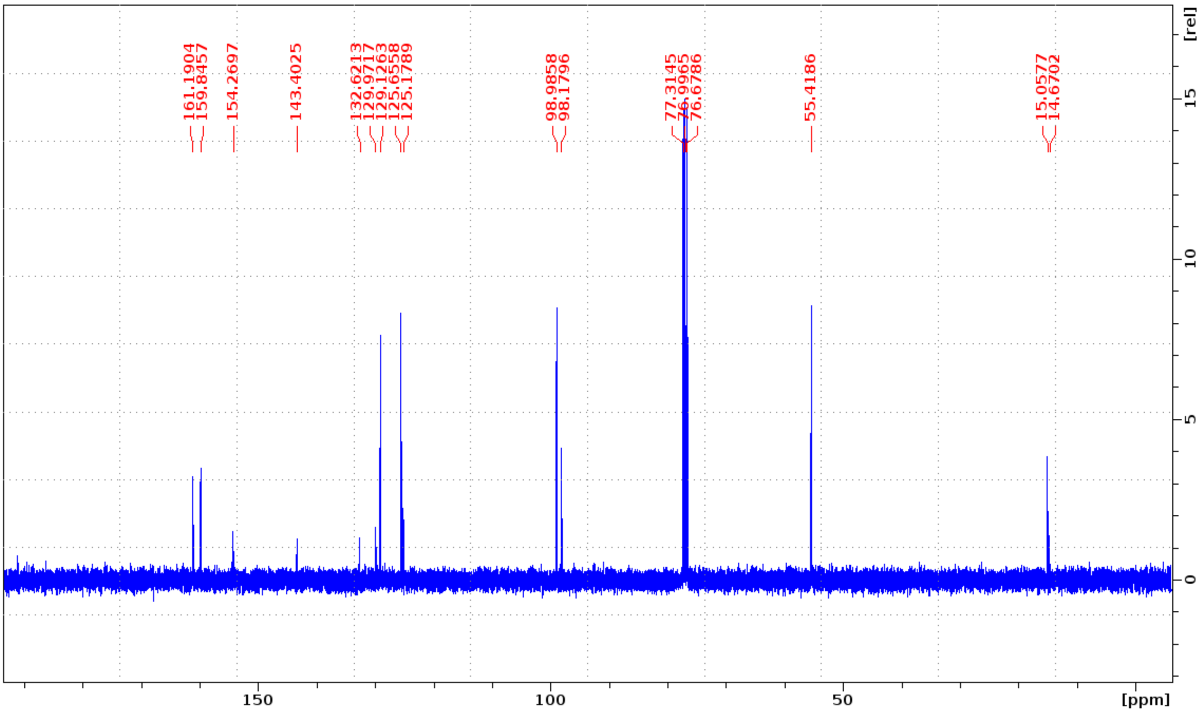

Figure S9: ^{13}C NMR Spectrum (*E*)-N-(3,5-Dimethoxyphenyl)-1-(4-(methylthio)phenyl)methanimine **(11c)**

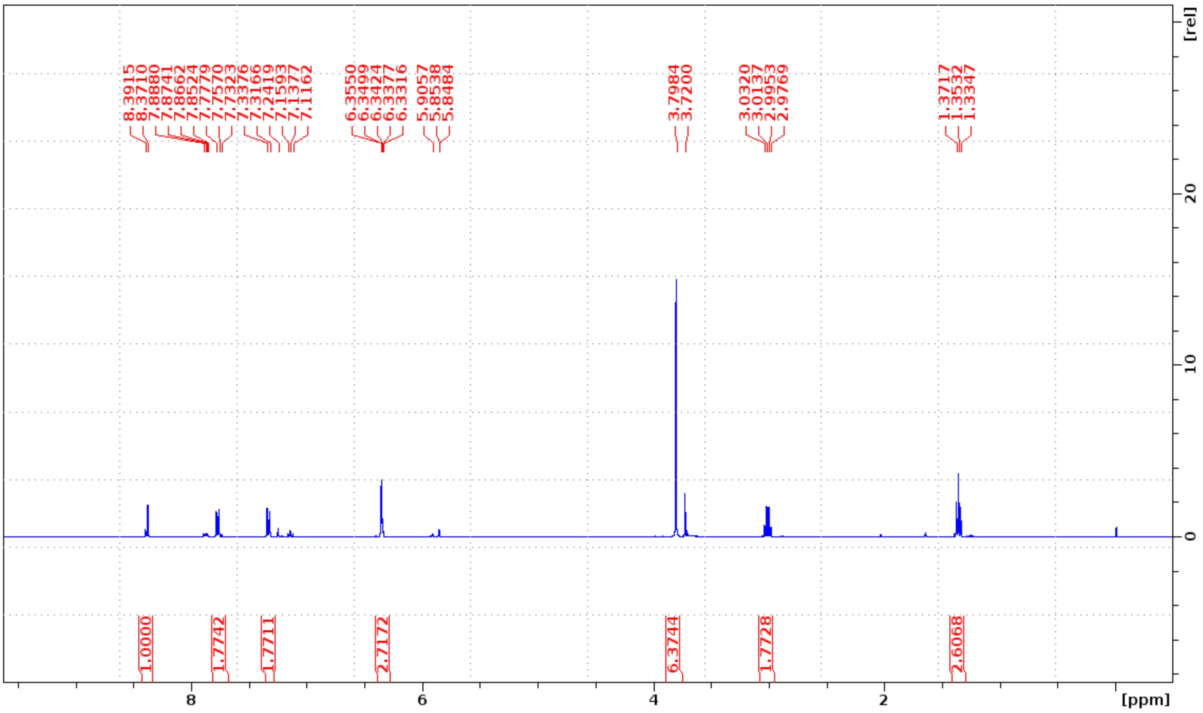

Figure S10: ^{1}H NMR Spectrum (*E*)-*N*-(4-(Ethylthio)benzylidene)-3,5-dimethoxyaniline **(11d)** [δ 3.73 impurity]

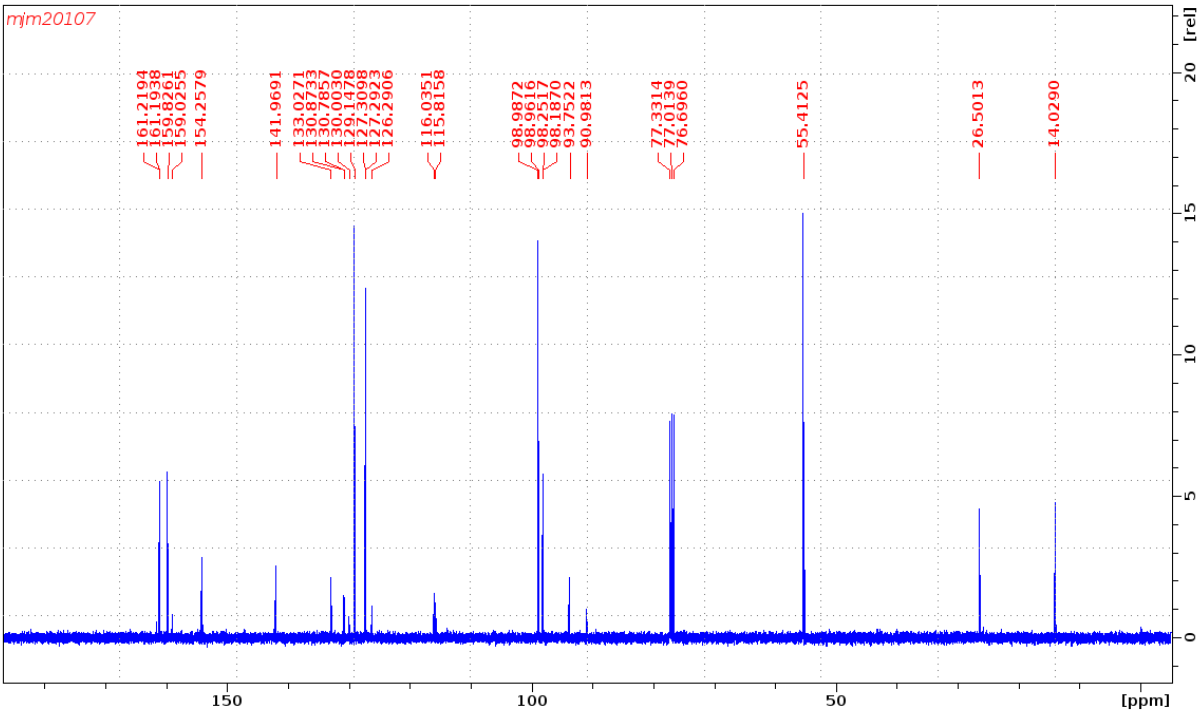

Figure S11: ^{13}C NMR Spectrum (*E*)-*N*-(4-(Ethylthio)benzylidene)-3,5-dimethoxyaniline **(11d)**

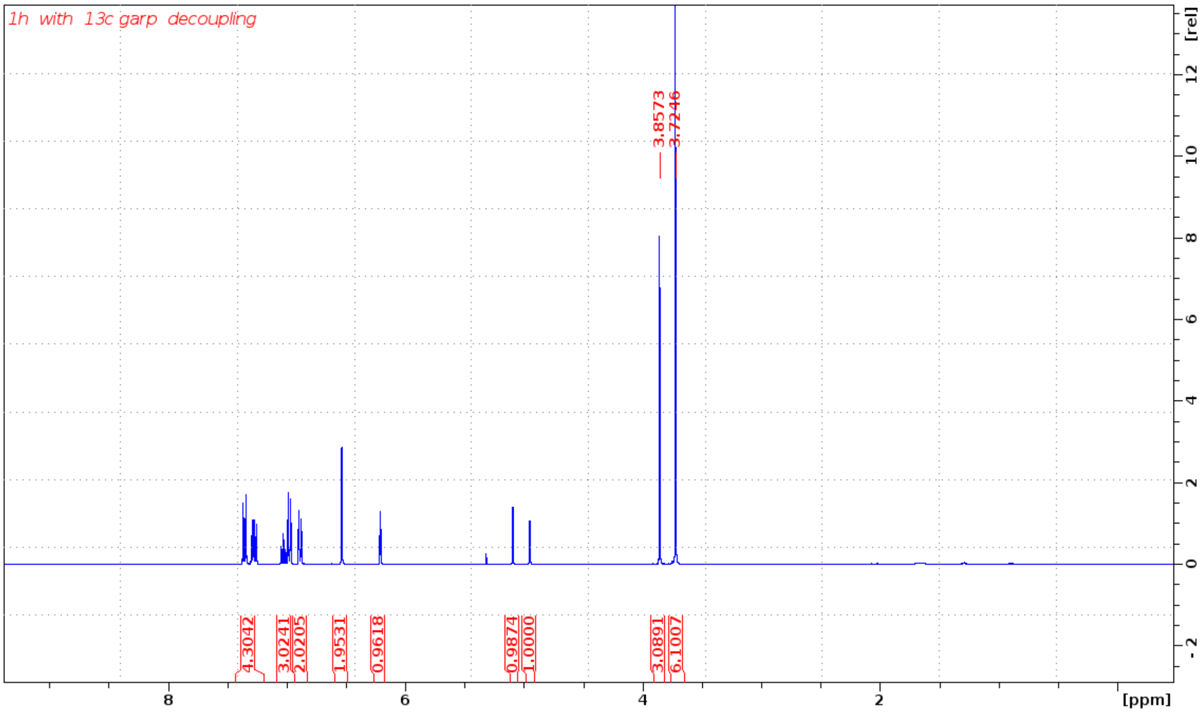

Figure S12: ^{1}H NMR Spectrum 1-(3,5-Dimethoxyphenyl)-4-(4-methoxyphenyl)-3-phenylazetidin-2-one (**12a)** [δ 1.52 (impurity H_2O), δ 2.04 (impurity acetone), δ 3.79]

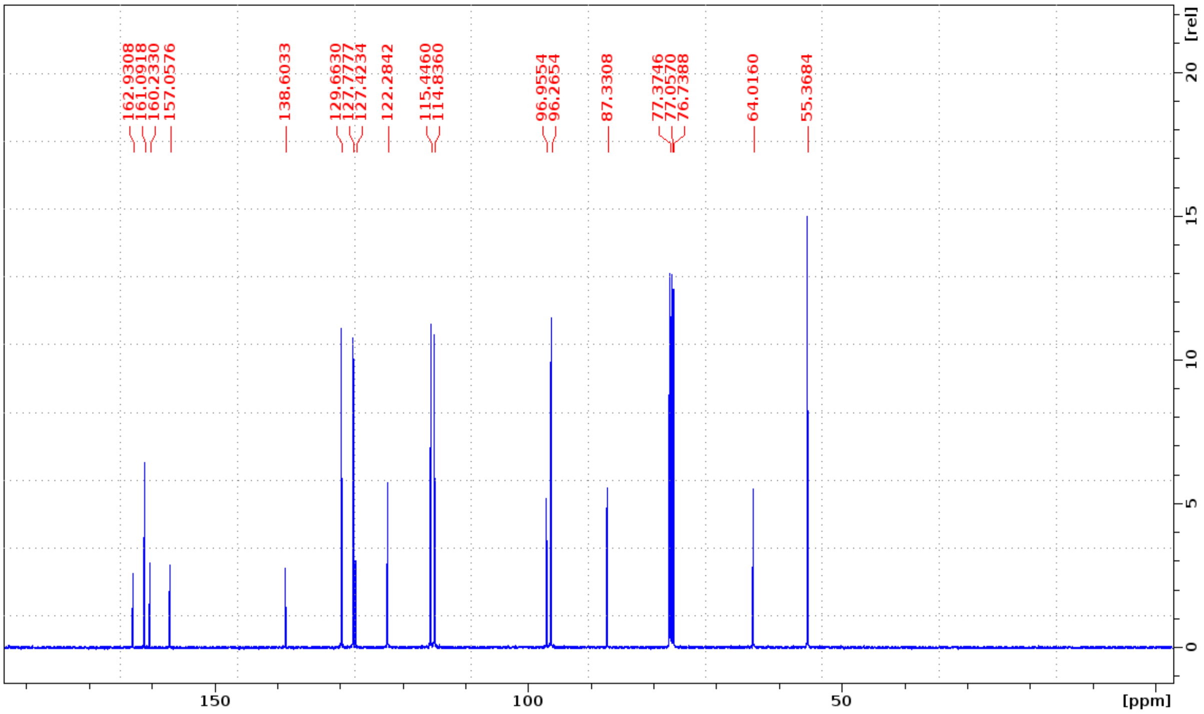

Figure S13: ^{13}C NMR Spectrum 1-(3,5-Dimethoxyphenyl)-4-(4-methoxyphenyl)-3-phenylazetidin-2-one (12a)

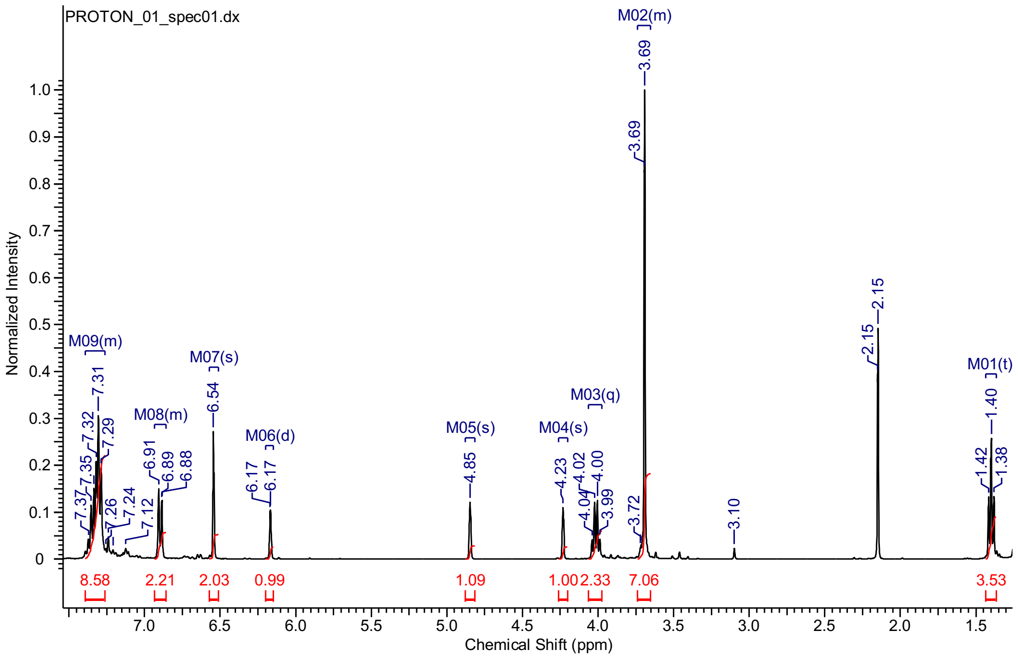

Figure S14: ^{1}H NMR Spectrum 1-(3,5-Dimethoxyphenyl)-4-(4-ethoxyphenyl)-3-phenylazetidin-2-one (12b) [Impurity δ 2.15 (acetone), δ 3.72, δ 3.10]

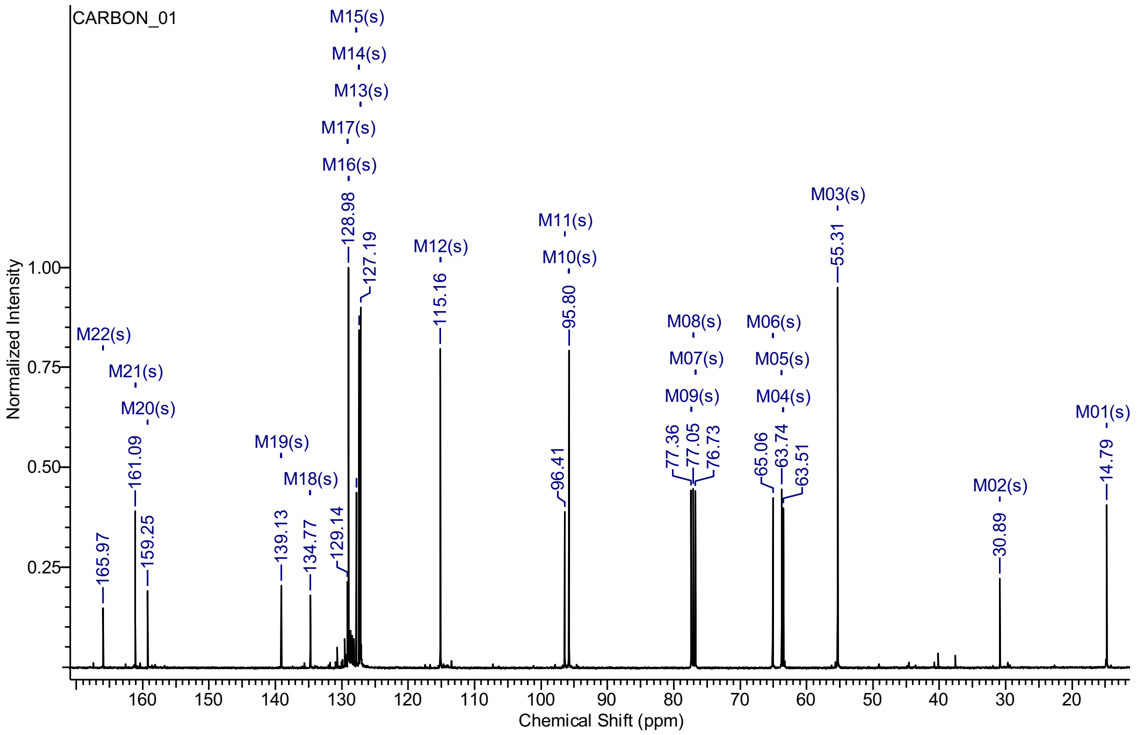

Figure S15: ^{13}C NMR Spectrum 1-(3,5-Dimethoxyphenyl)-4-(4-ethoxyphenyl)-3-phenylazetidin-2-one (12b)

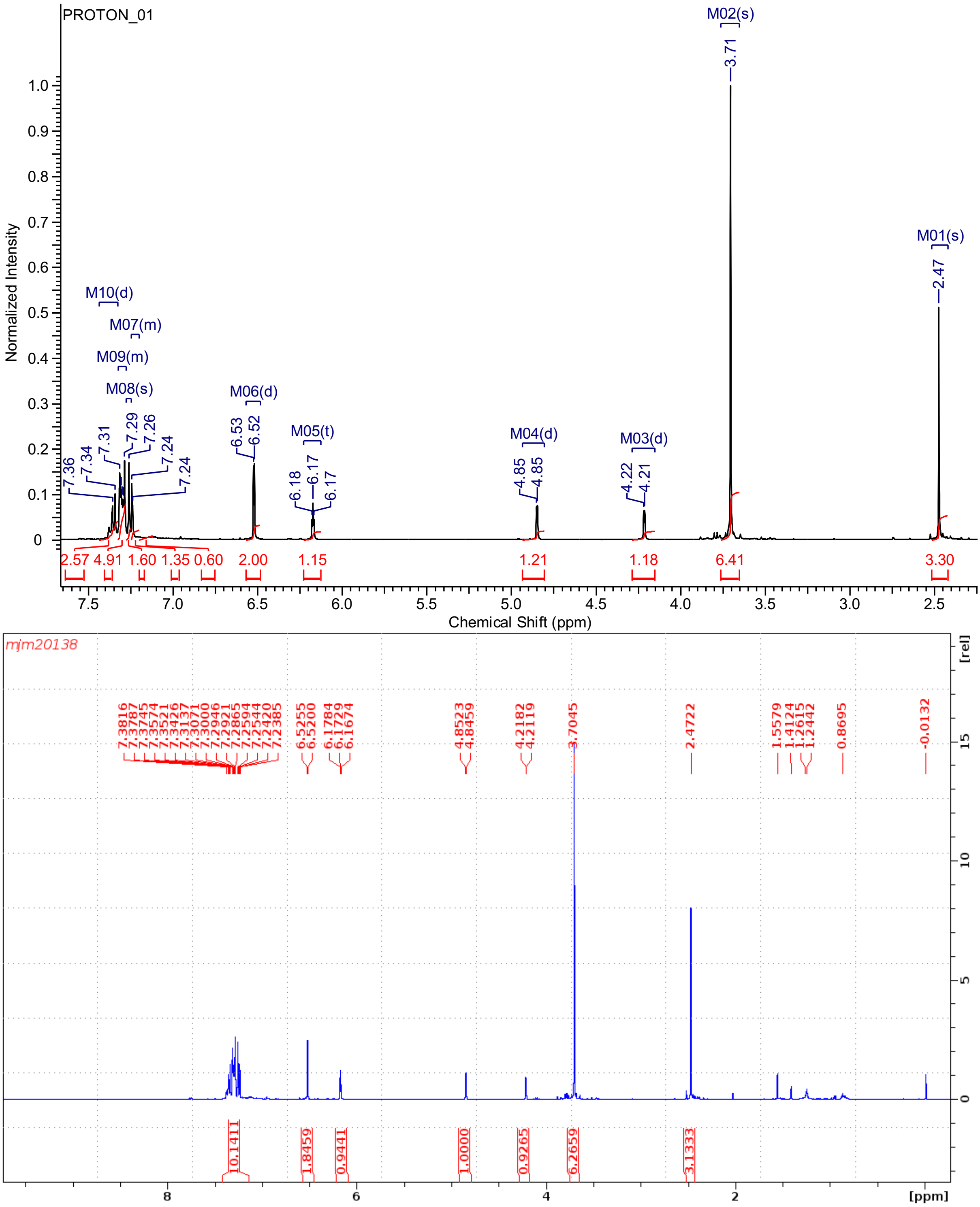

Figure S16: ^{1}H NMR Spectrum 1-(3,5-Dimethoxyphenyl)-4-(4-(methylthio)phenyl)-3-phenylazetidin-2-one (12c) [impurity H_2O at δ 1.56, Ethyl acetate impurity at δ 1.24, 2.02, 4.12]

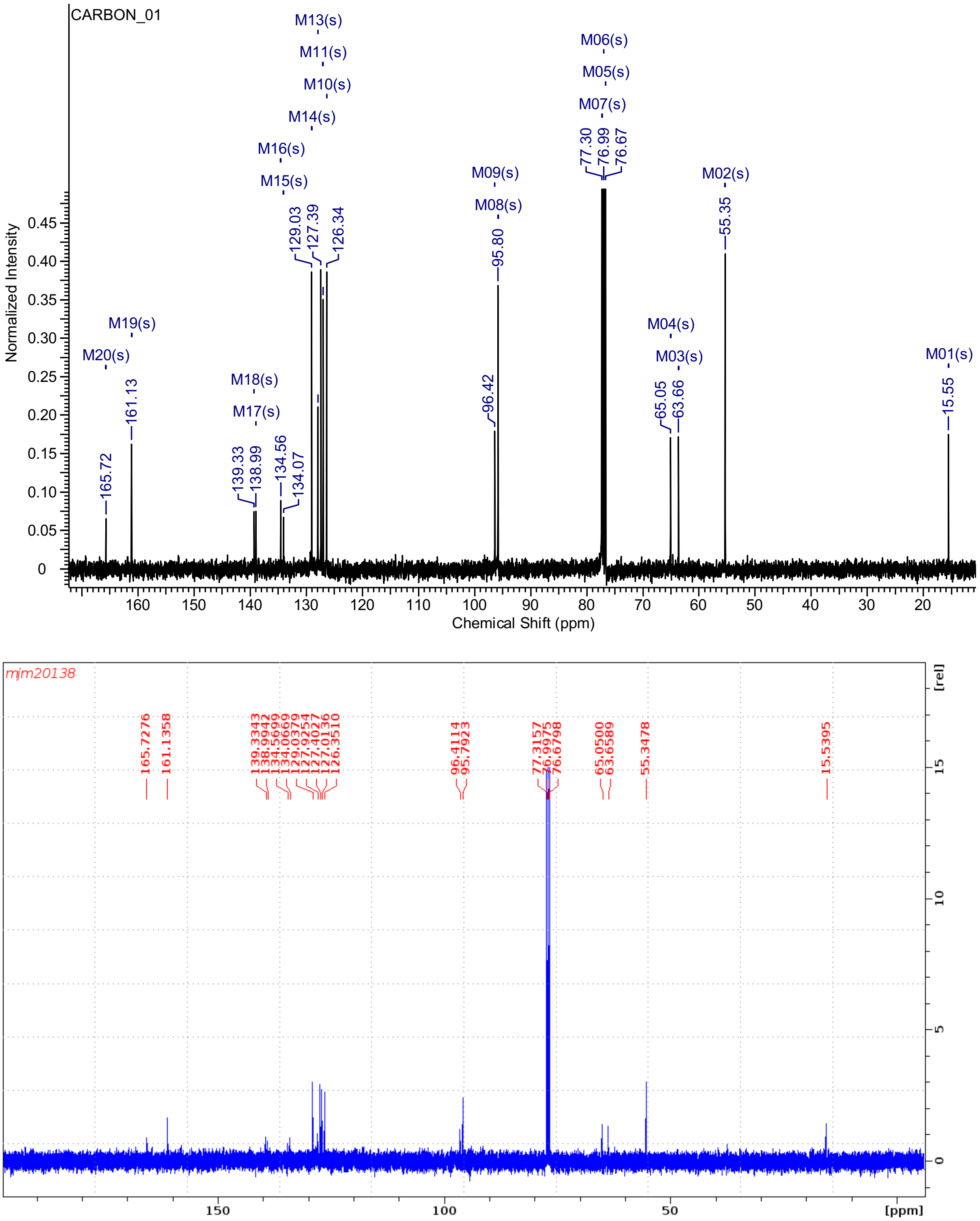

Figure S17: ^{13}C NMR Spectrum 1-(3,5-Dimethoxyphenyl)-4-(4-(methylthio)phenyl)-3-phenylazetidin-2-one (12c)

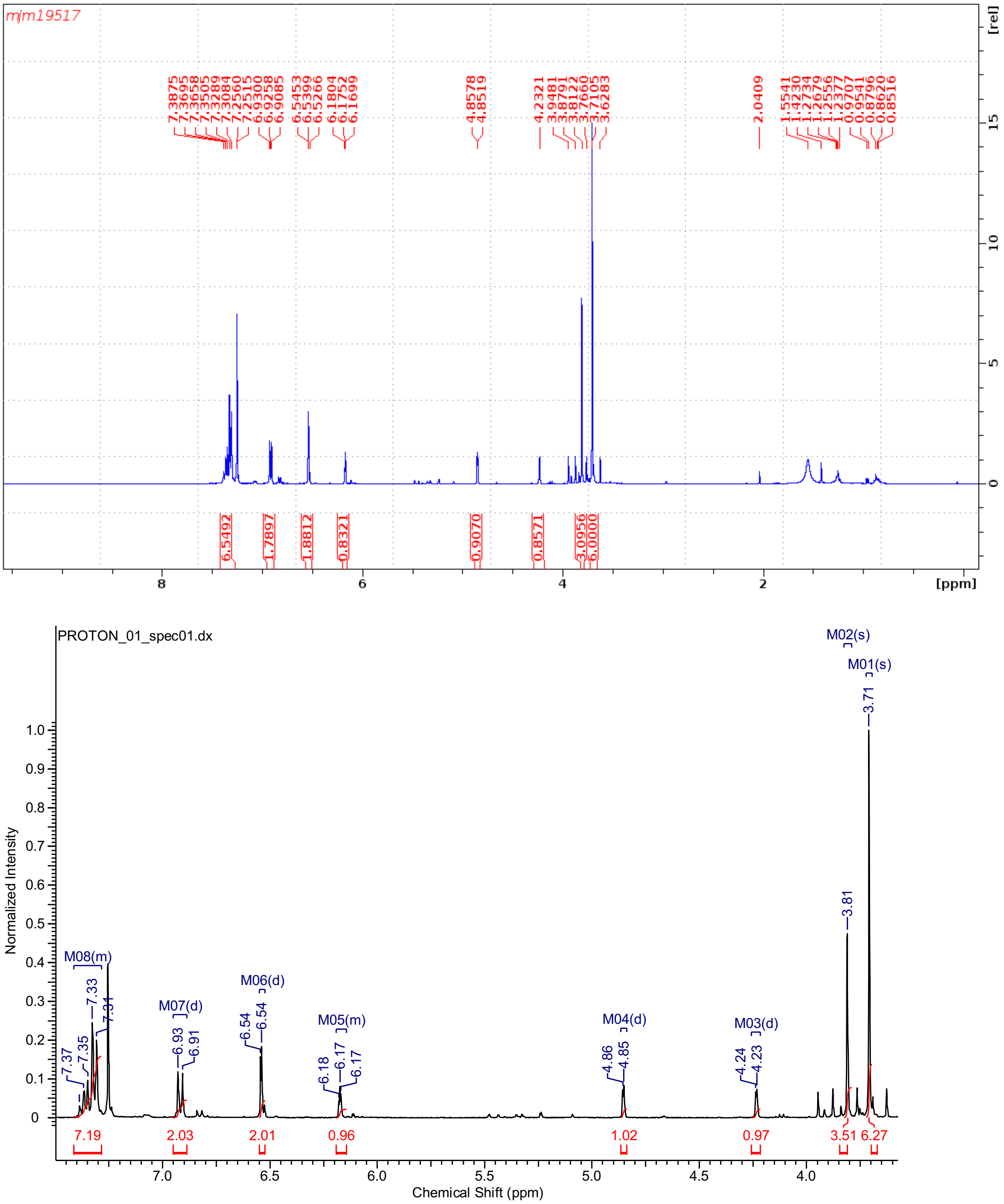

Figure S18: ^{1}H NMR Spectrum 1-(3,5-Dimethoxyphenyl)-4-(4-methoxyphenyl)-3-phenoxyazetidin-2-one (12d) [δ 1.55 impurity H_2O), δ 2.02 (acetone), δ 1.24, 2.02, 4.12 (Ethyl acetate)]

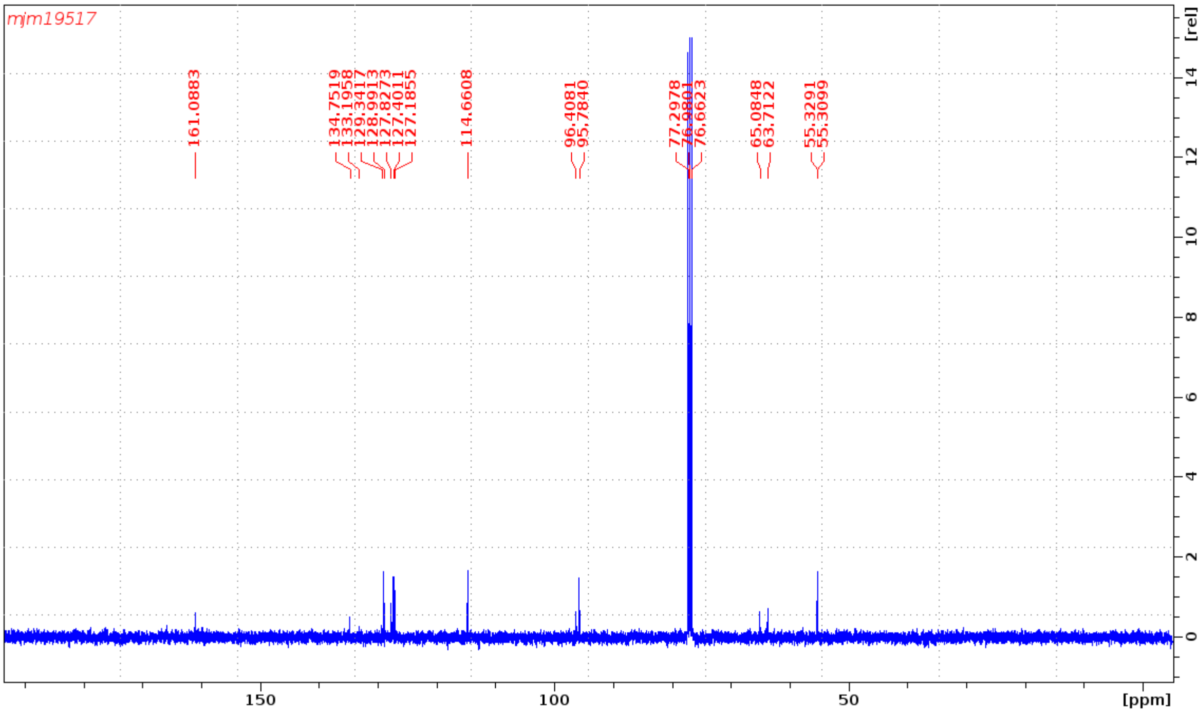

Figure S19: ^{13}**C NMR Spectrum 1-(3,5-Dimethoxyphenyl)-4-(4-methoxyphenyl)-3-phenoxyazetidin-2-one (12d)**

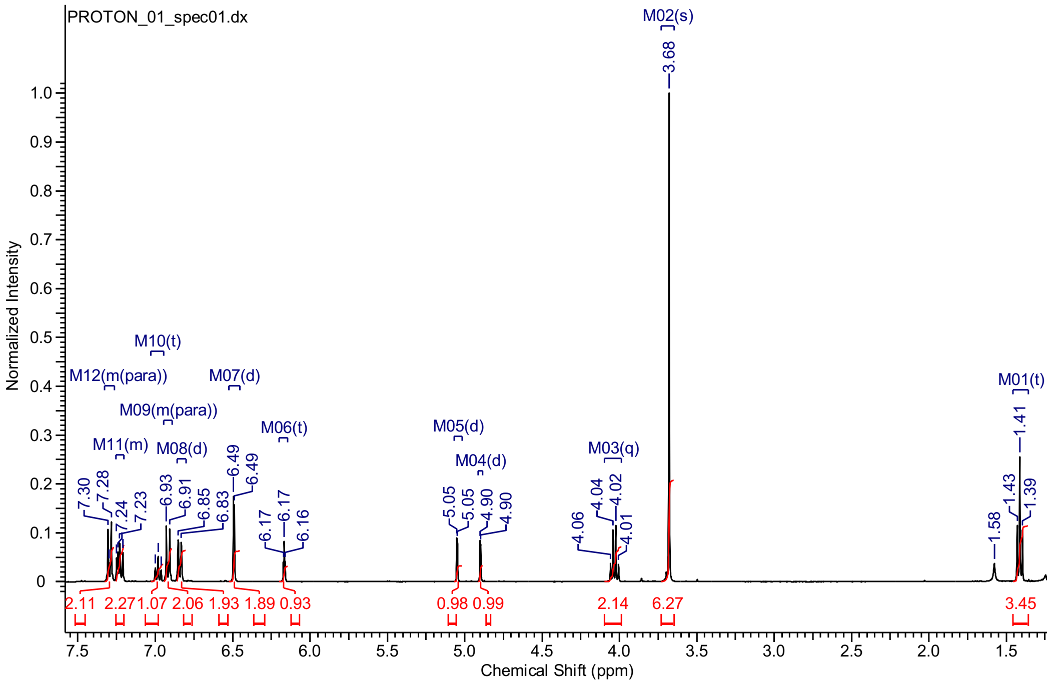

Figure S20: ^{1}H NMR Spectrum 1-(3,5-Dimethoxyphenyl)-4-(4-ethoxyphenyl)-3-phenoxyazetidin-2-one *trans* (**12e**) (impurity H_2O at δ 1.58)

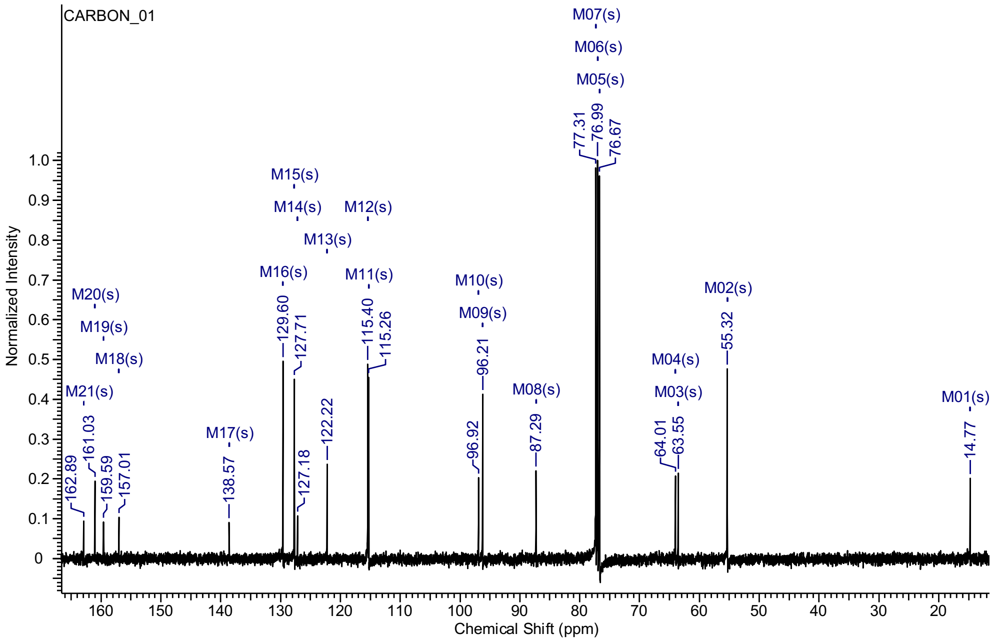

Figure S21: ^{13}C NMR Spectrum 1-(3,5-Dimethoxyphenyl)-4-(4-ethoxyphenyl)-3-phenoxyazetidin-2-one (12e) *trans*

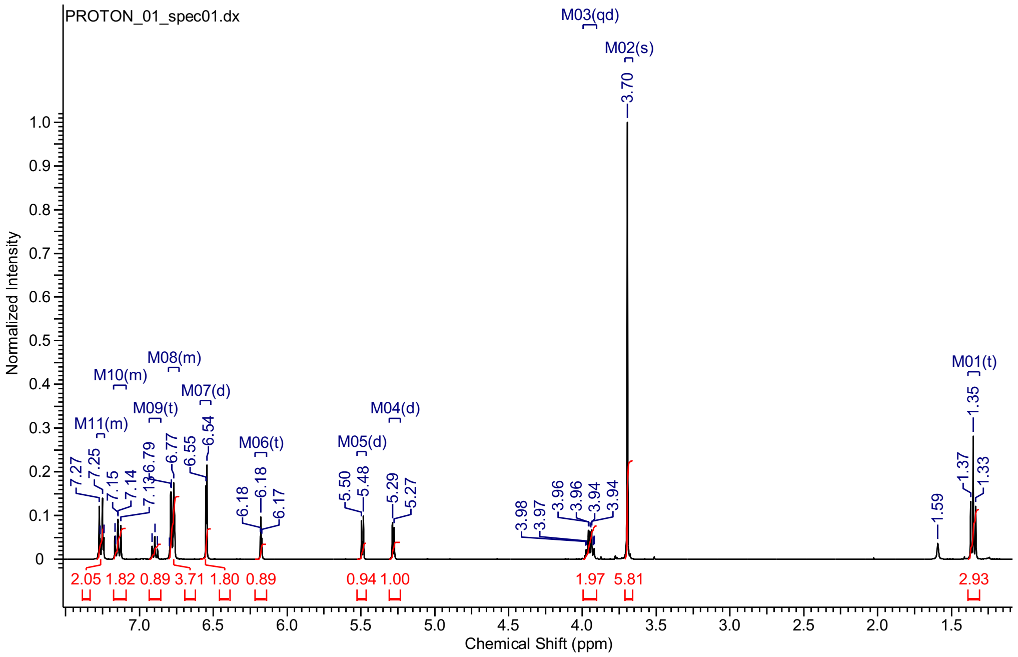

Figure S22: ^{1}H NMR Spectrum 1-(3,5-Dimethoxyphenyl)-4-(4-ethoxyphenyl)-3-phenoxyazetidin-2-one *cis* **(12e)** [impurity H_2O at δ 1.59]

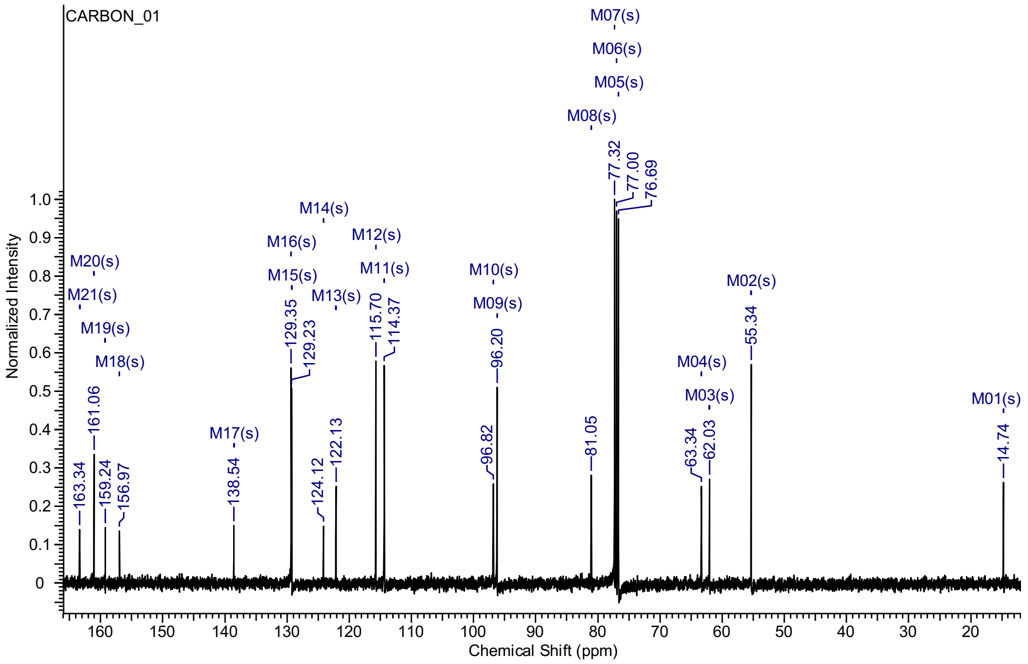

Figure S23: ^{13}C NMR Spectrum 1-(3,5-Dimethoxyphenyl)-4-(4-ethoxyphenyl)-3-phenoxyazetidin-2-one (**12e**) *cis*

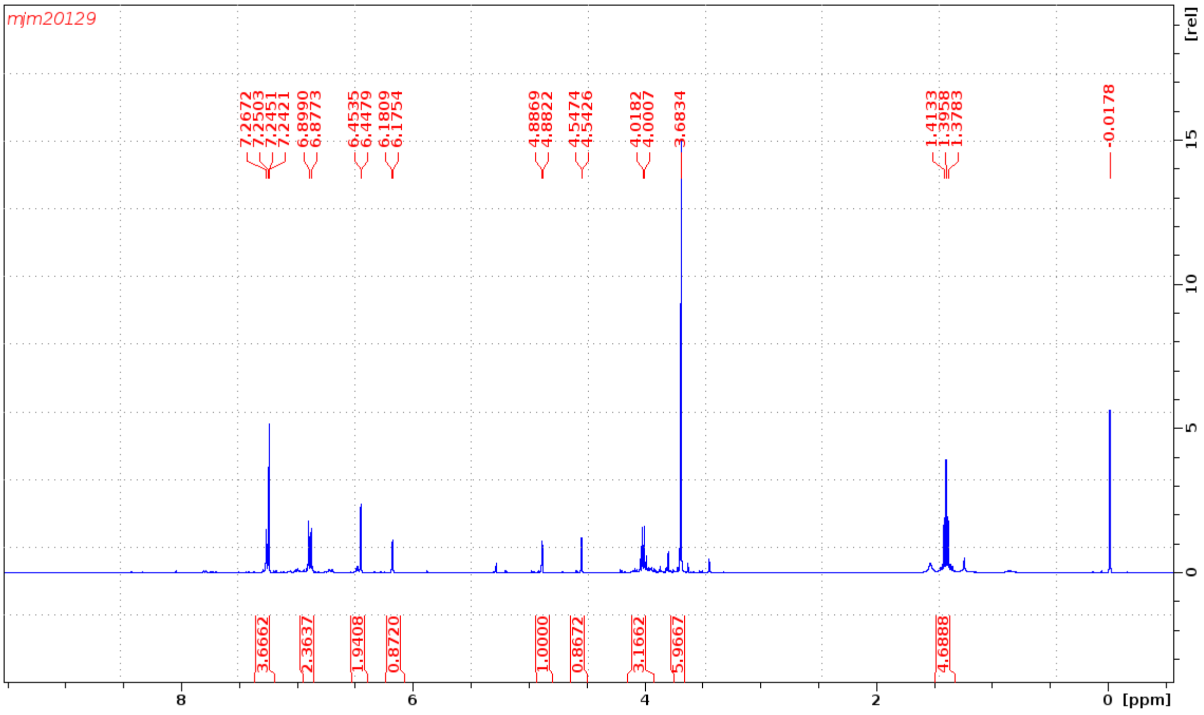

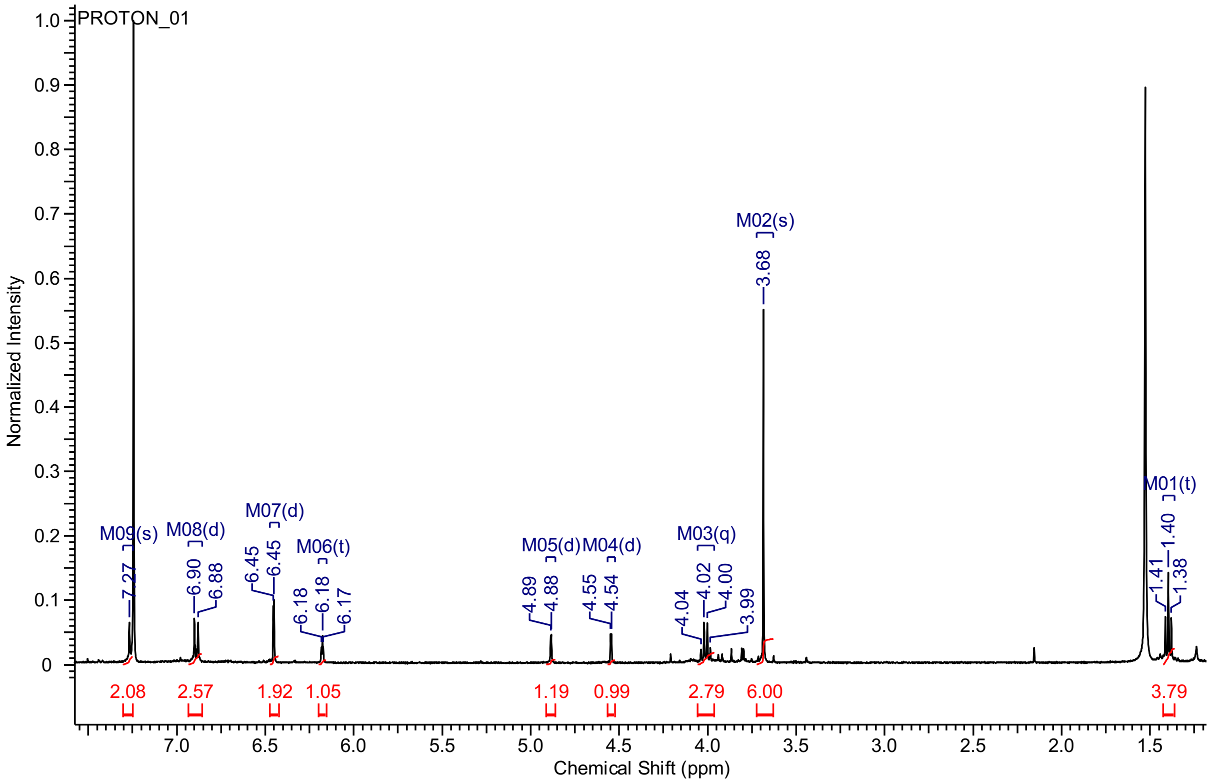

Figure S24: ^{1}H NMR Spectrum 3-Chloro-1-(3,5-dimethoxyphenyl)-4-(4-ethoxyphenyl) azetidin-2-one **(12g)** [impurity H_2O at δ 1.58]

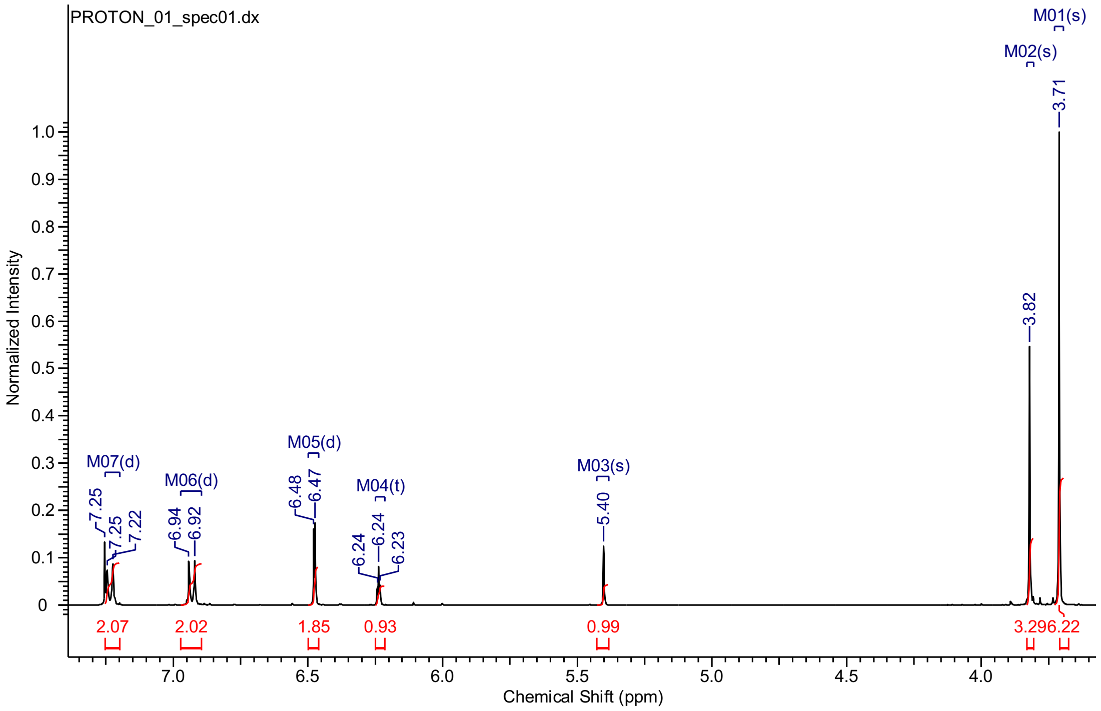

Figure S25: ^{1}H NMR Spectrum 3,3-Dichloro-1-(3,5-dimethoxyphenyl)-4-(4-methoxyphenyl) azetidin-2-one **(12h)**

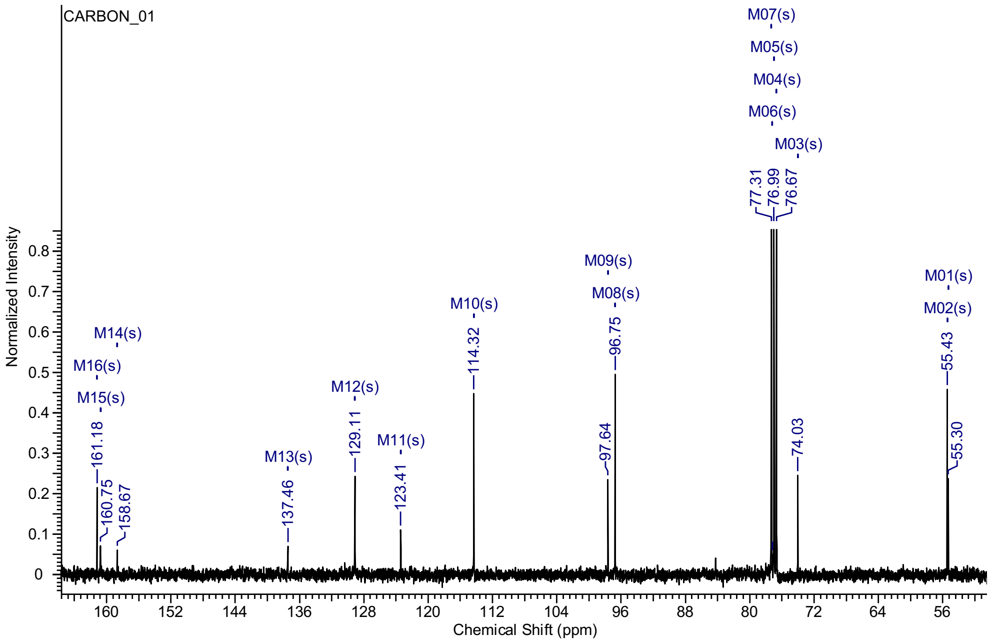

Figure S26: ^{13}C NMR Spectrum 3,3-Dichloro-1-(3,5-dimethoxyphenyl)-4-(4-methoxyphenyl) azetidin-2-one **(12h)**

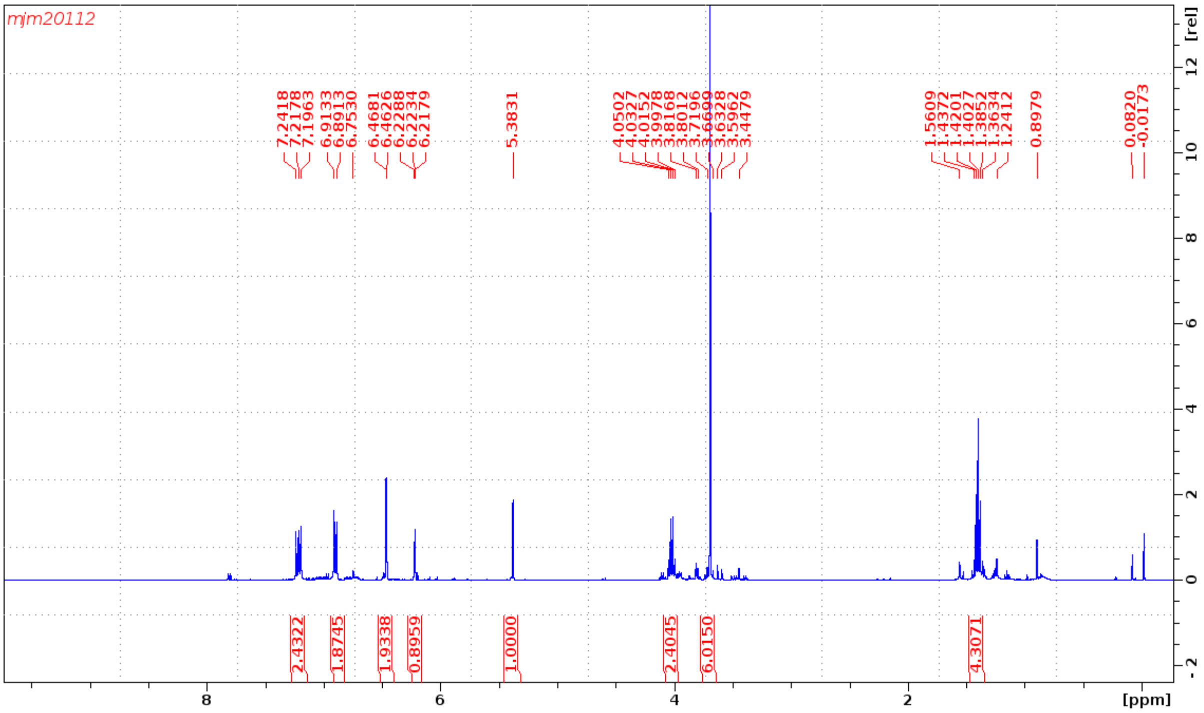

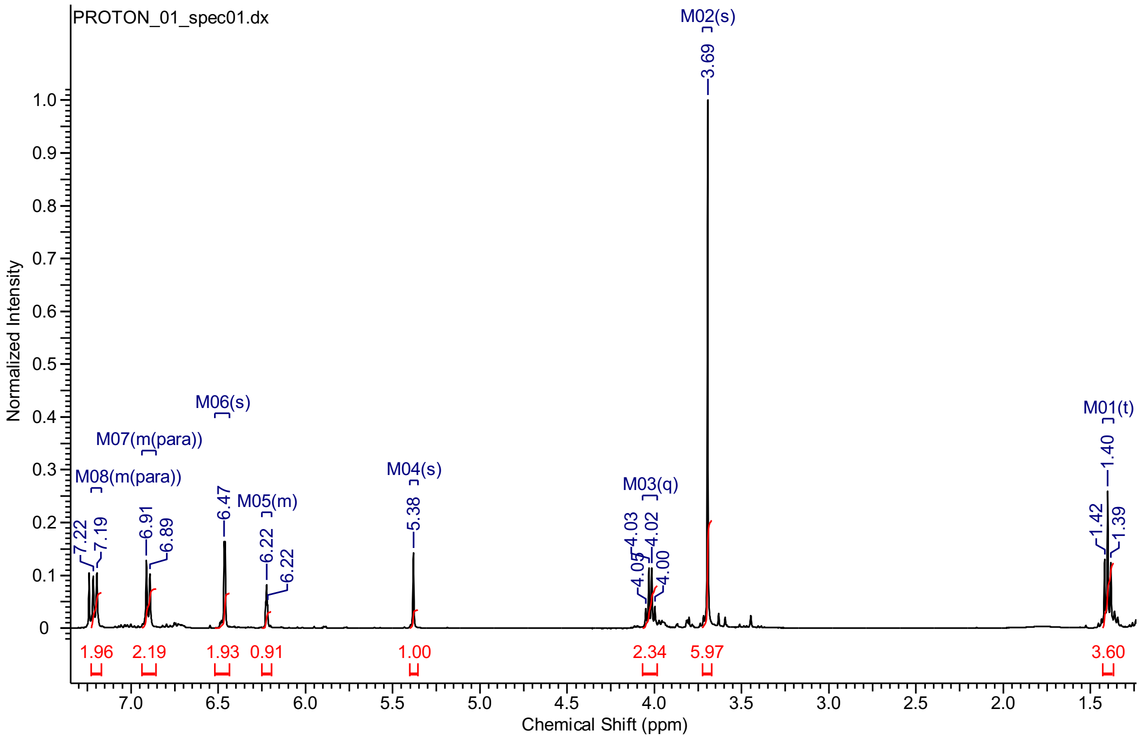

Figure S27: ^{1}H NMR Spectrum 3,3-Dichloro-1-(3,5-dimethoxyphenyl)-4-(4-ethoxyphenyl) azetidin-2-one **(12i)** [impurity H_2O at δ 1.56]

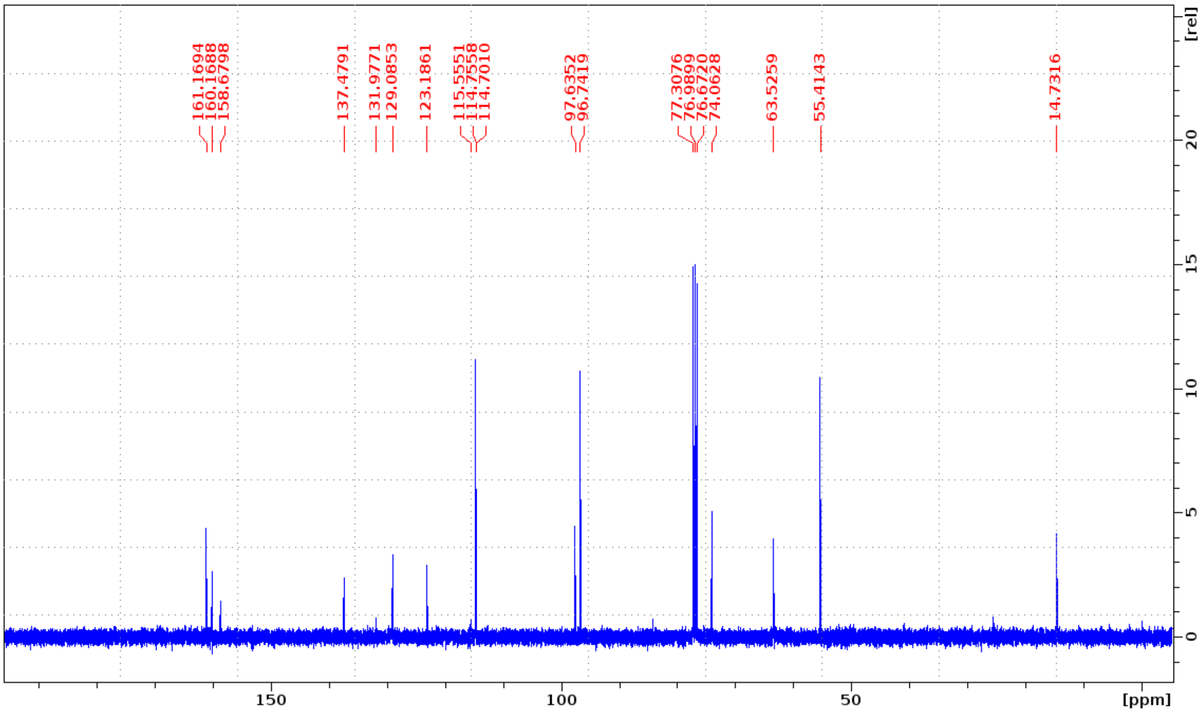

Figure S28: ^{13}C NMR Spectrum 3,3-Dichloro-1-(3,5-dimethoxyphenyl)-4-(4-ethoxyphenyl) azetidin-2-one **(12i)**

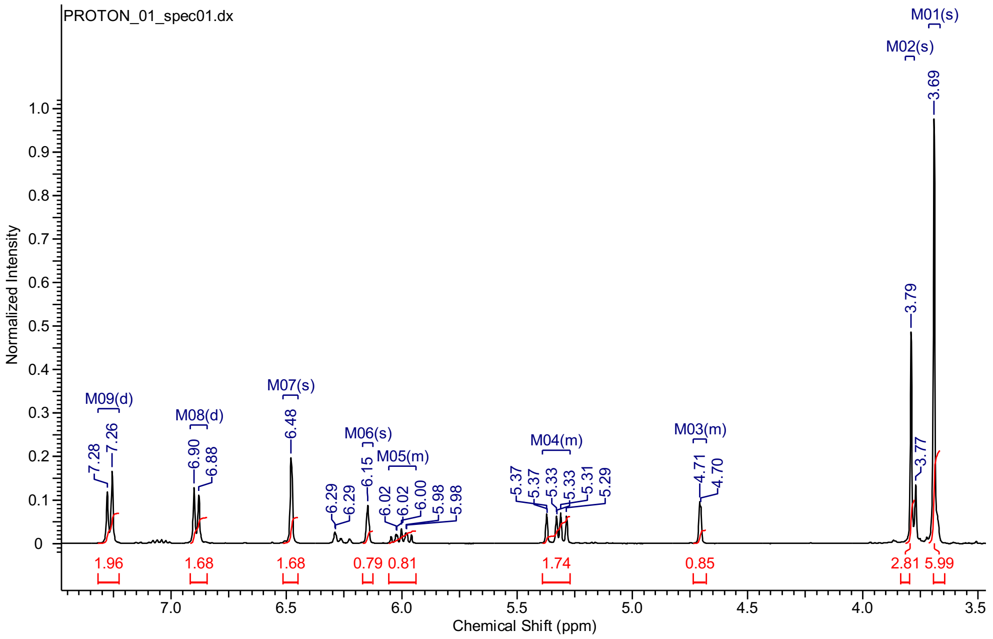

Figure S29: ^{1}H NMR Spectrum 1-(3,5-Dimethoxyphenyl)-4-(4-methoxyphenyl)-3-vinylazetidin-2-one **(12j)** [Impurity δ 3.77]

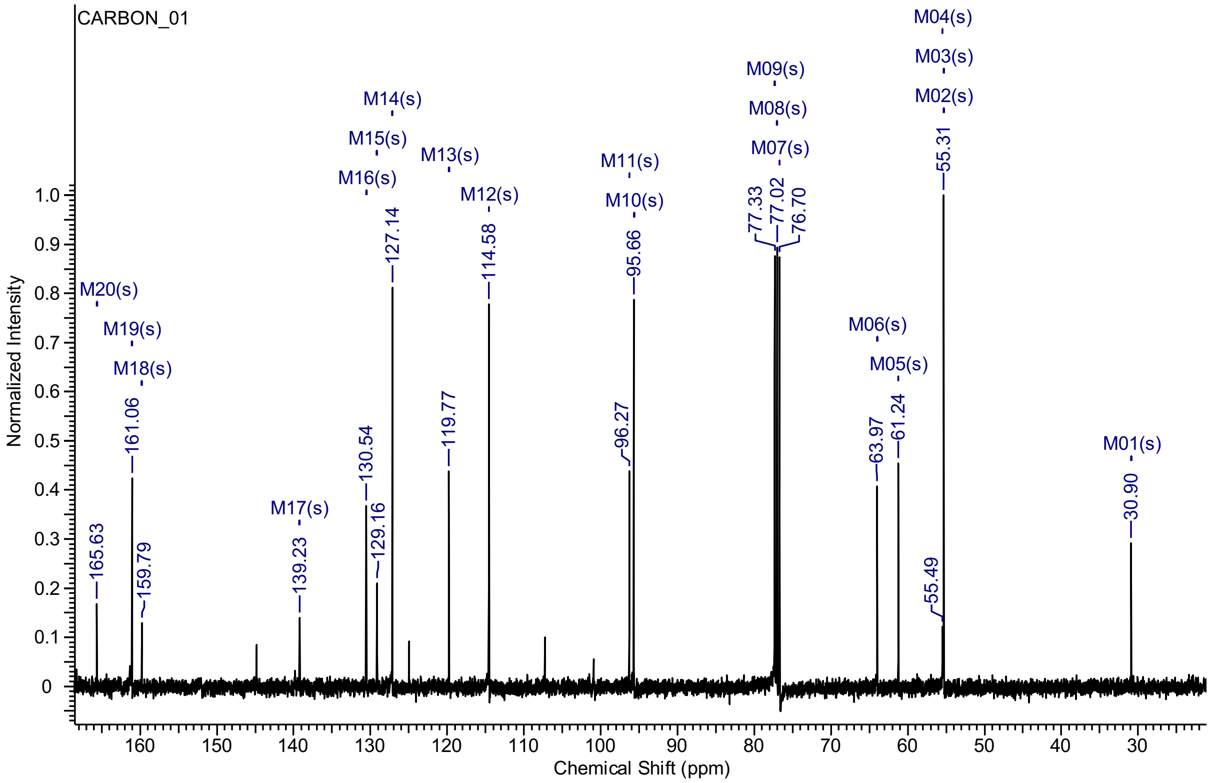

Figure S30: ^{13}C NMR Spectrum 1-(3,5-Dimethoxyphenyl)-4-(4-methoxyphenyl)-3-vinylazetidin-2-one **(12j)**

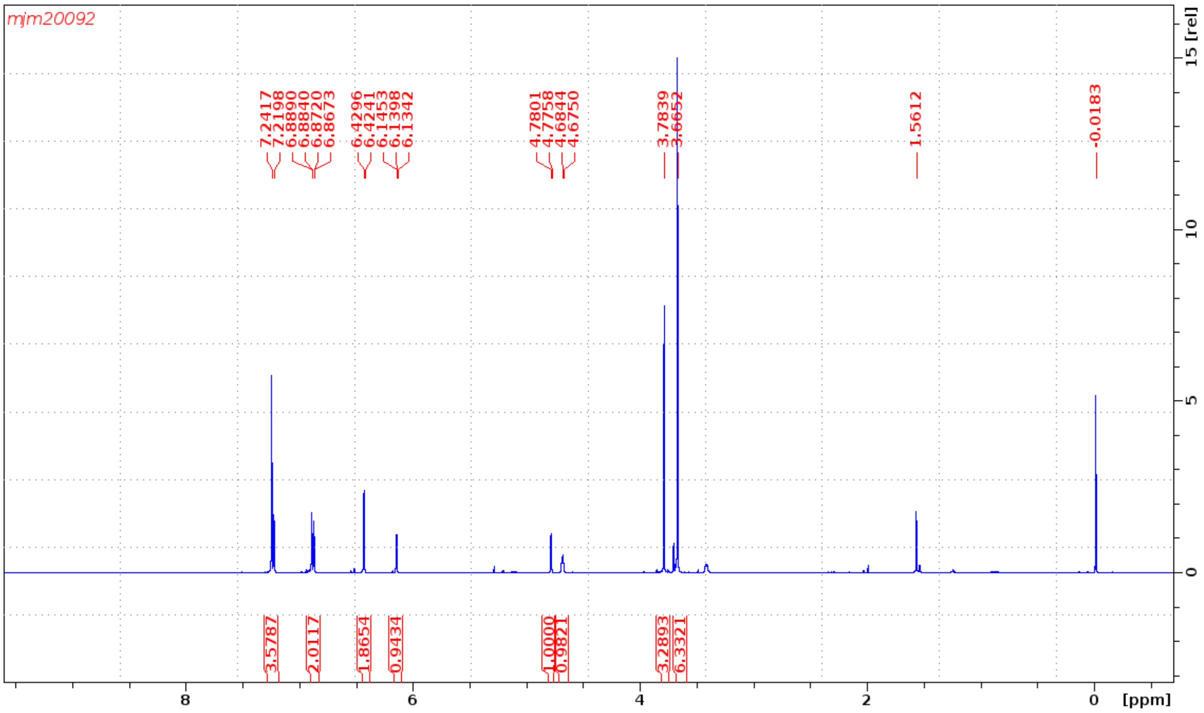

Figure S31: ^{1}H NMR Spectrum 1-(3,5-Dimethoxyphenyl)-3-hydroxy-4-(4-methoxyphenyl)azetidin-2-one **(12k)** (impurity H_2O at δ 1.56)

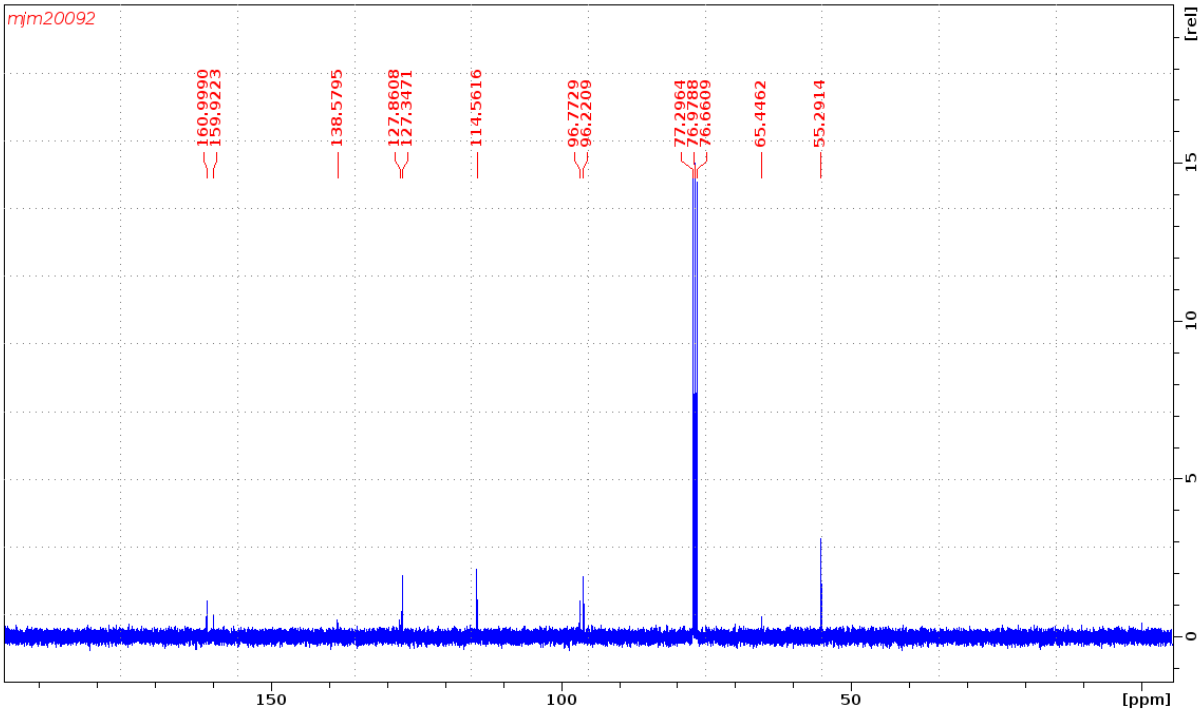

Figure S32: ^{13}C NMR Spectrum 1-(3,5-Dimethoxyphenyl)-3-hydroxy-4-(4-methoxyphenyl)azetidin-2-o**ne (12k)**

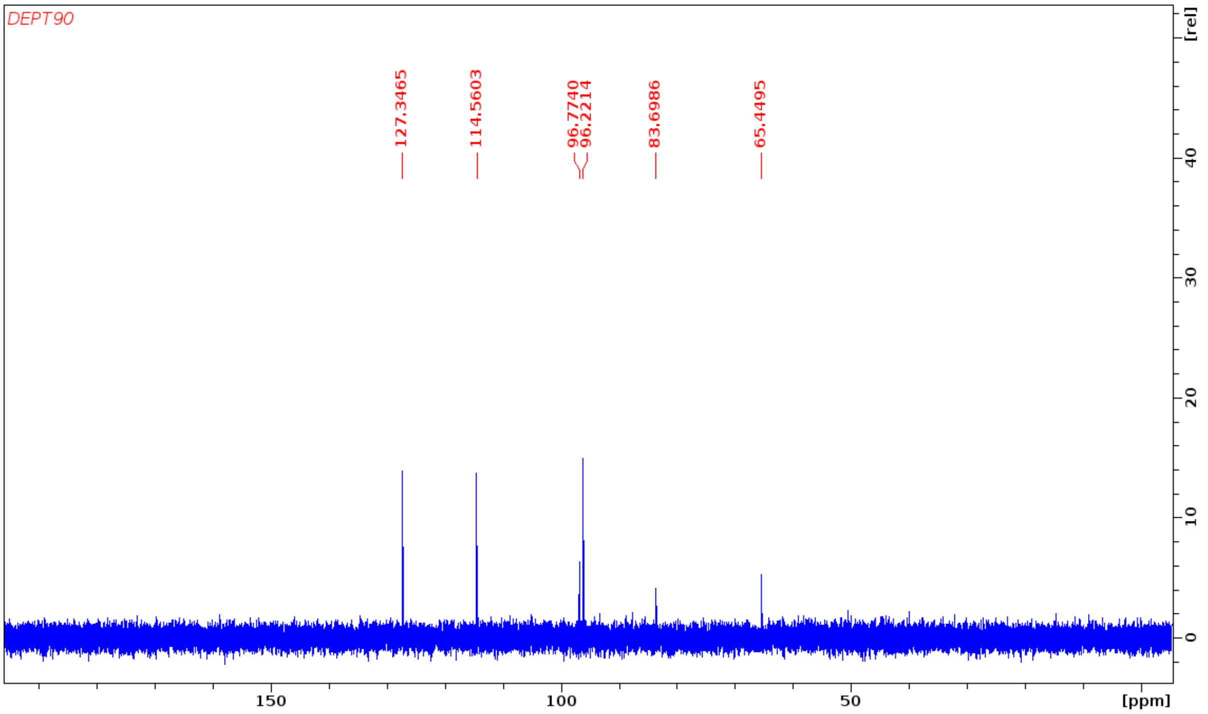

Figure S33: ^{13}C NMR DEPT-90 Spectrum 1-(3,5-Dimethoxyphenyl)-3-hydroxy-4-(4-methoxyphenyl)azetidin-2-one **(12k)**

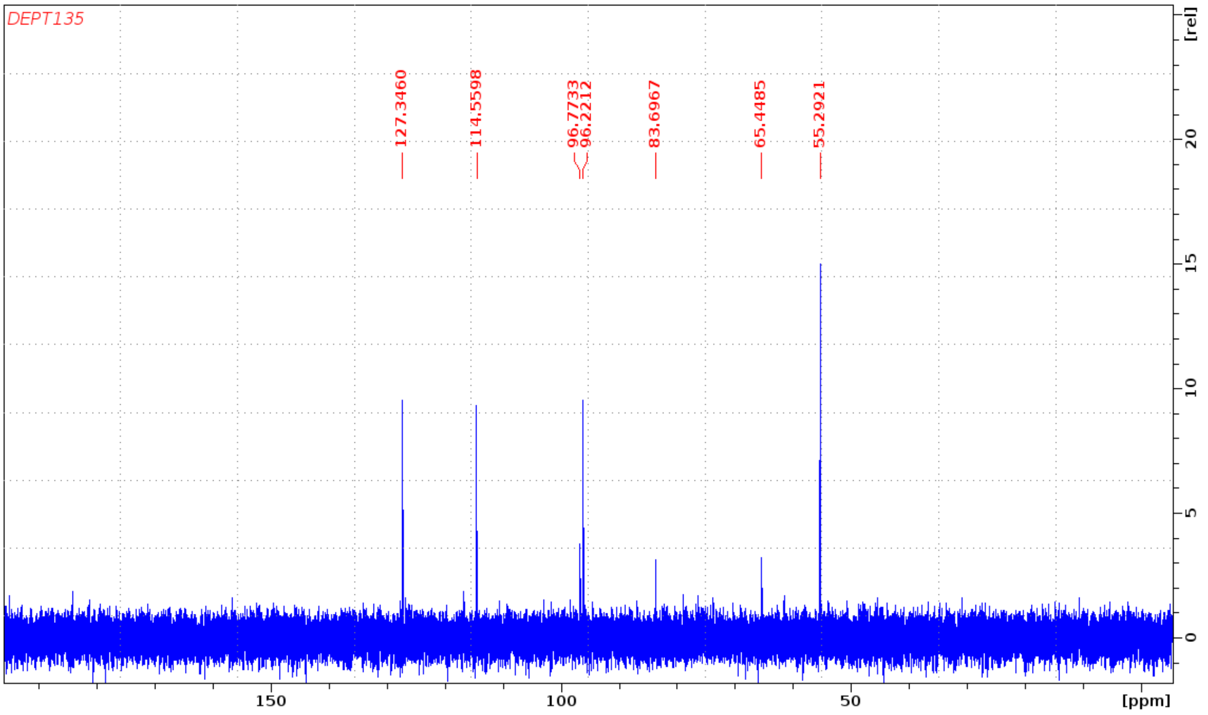

Figure S34: ^{13}C NMR DEPT-135 Spectrum 1-(3,5-Dimethoxyphenyl)-3-hydroxy-4-(4-methoxyphenyl)azetidin-2-one **(12k)**

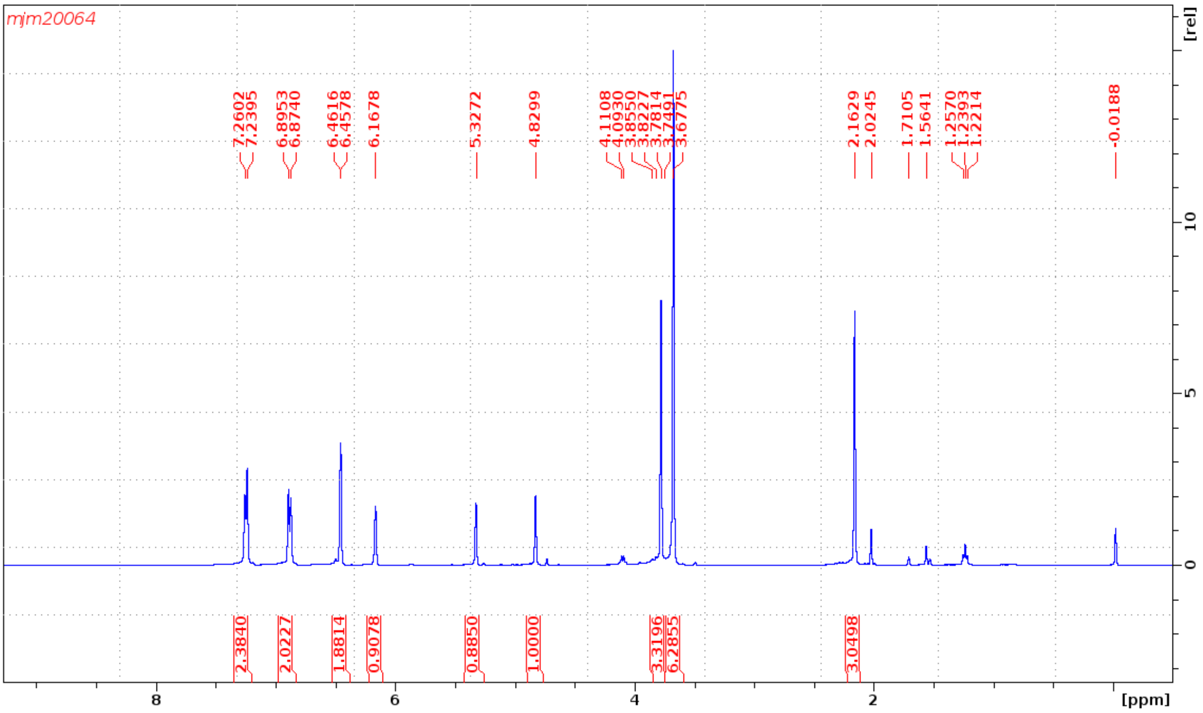

Figure S35: ^{1}H NMR Spectrum 3-Acetoxy-1-(3,5-dimethoxyphenyl)- 4-(4-methoxyphenyl)azetidin-2-one [Ethyl acetate impurity at δ 1.24, 2.02, 4.12, H_2O at δ 1.56]

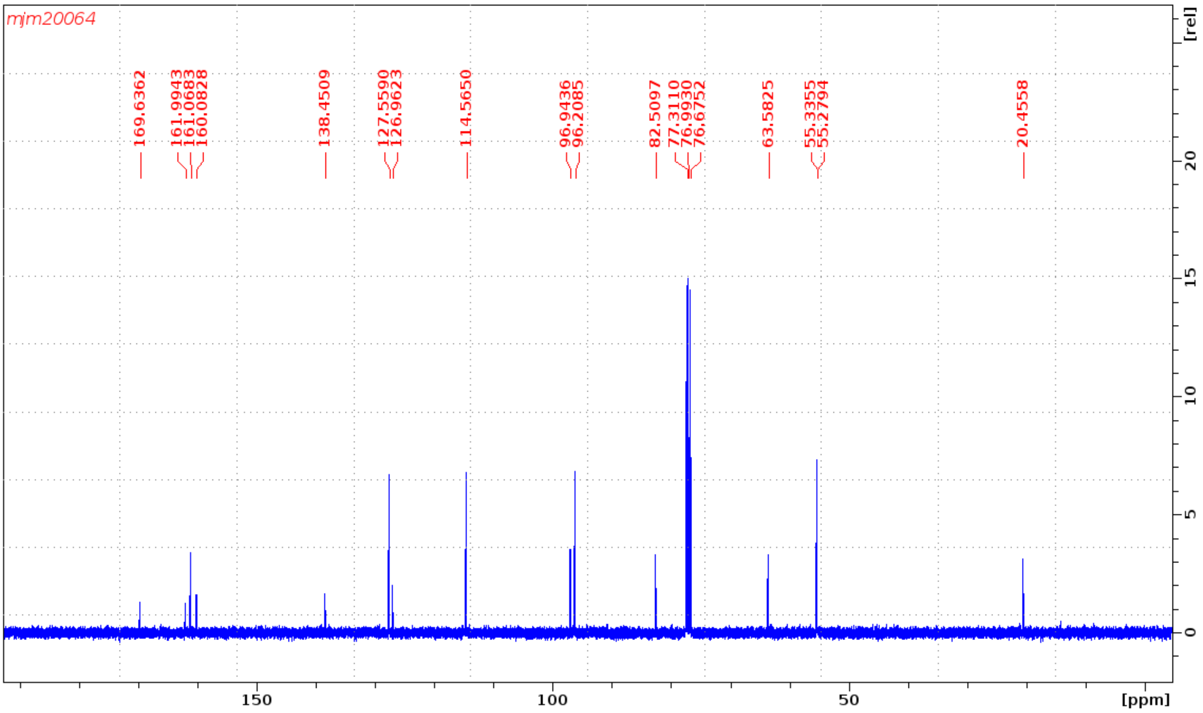

Figure S36: ^{13}C NMR Spectrum 3-Acetoxy-1-(3,5-dimethoxyphenyl)- 4-(4-methoxyphenyl)azetidin-2-one

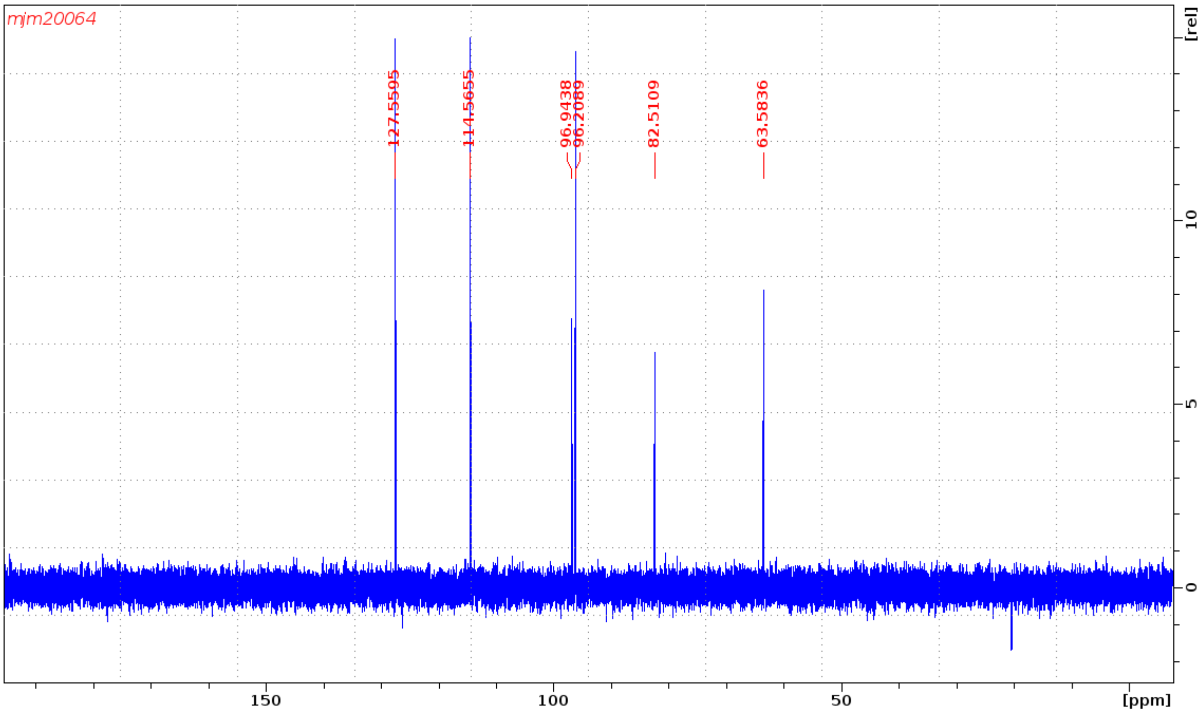

Figure S37: ^{13}C NMR DEPT 90 Spectrum 3-Acetoxy-1-(3,5-dimethoxyphenyl)- 4-(4-methoxyphenyl)azetidin-2-one

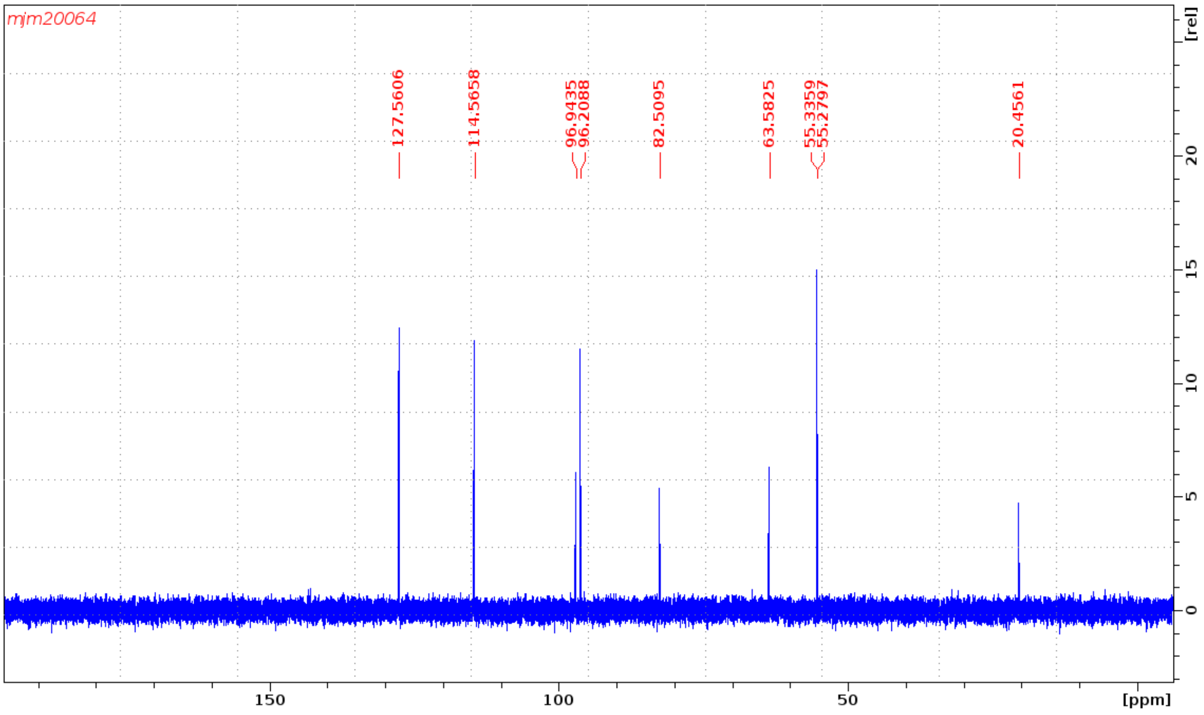

Figure S38: ^{13}C NMR DEPT 135 Spectrum 3-Acetoxy-1-(3,5-dimethoxyphenyl)- 4-(4-methoxyphenyl)azetidin-2-one

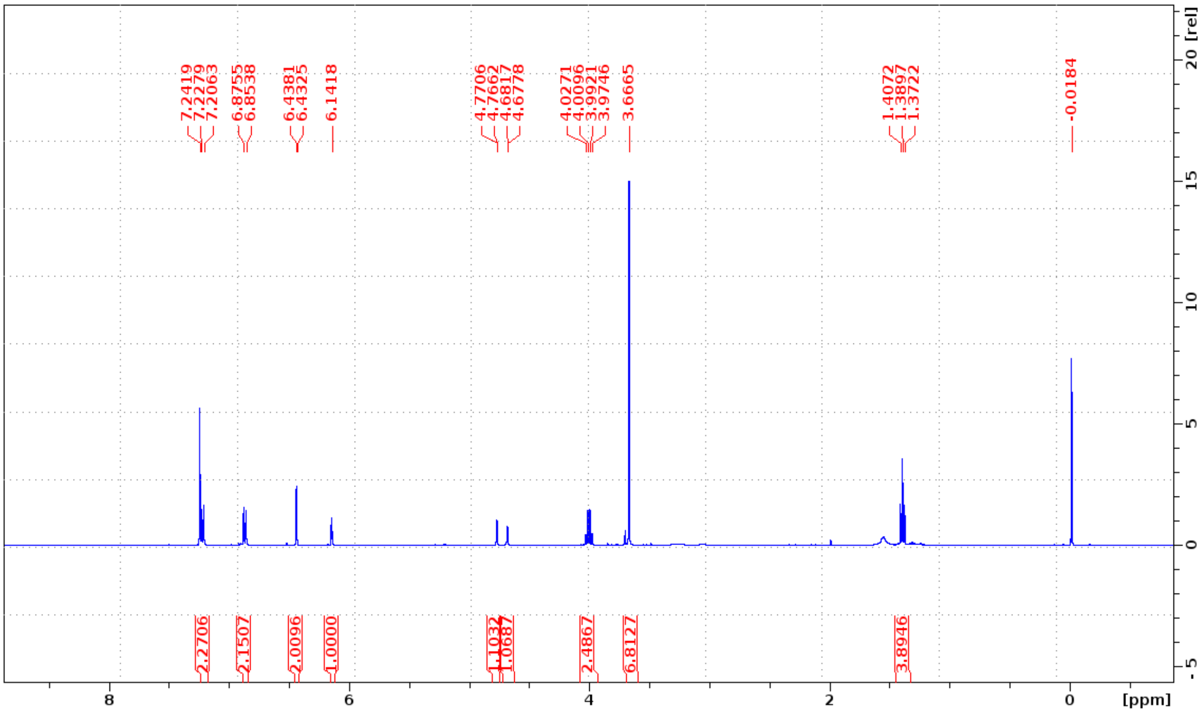

Figure S39: ^{1}H NMR Spectrum 1-(3,5-Dimethoxyphenyl)-4-(4-ethoxyphenyl)-3-hydroxyazetidin-2-one **(12l)** (impurity H_2O at δ 1.58)

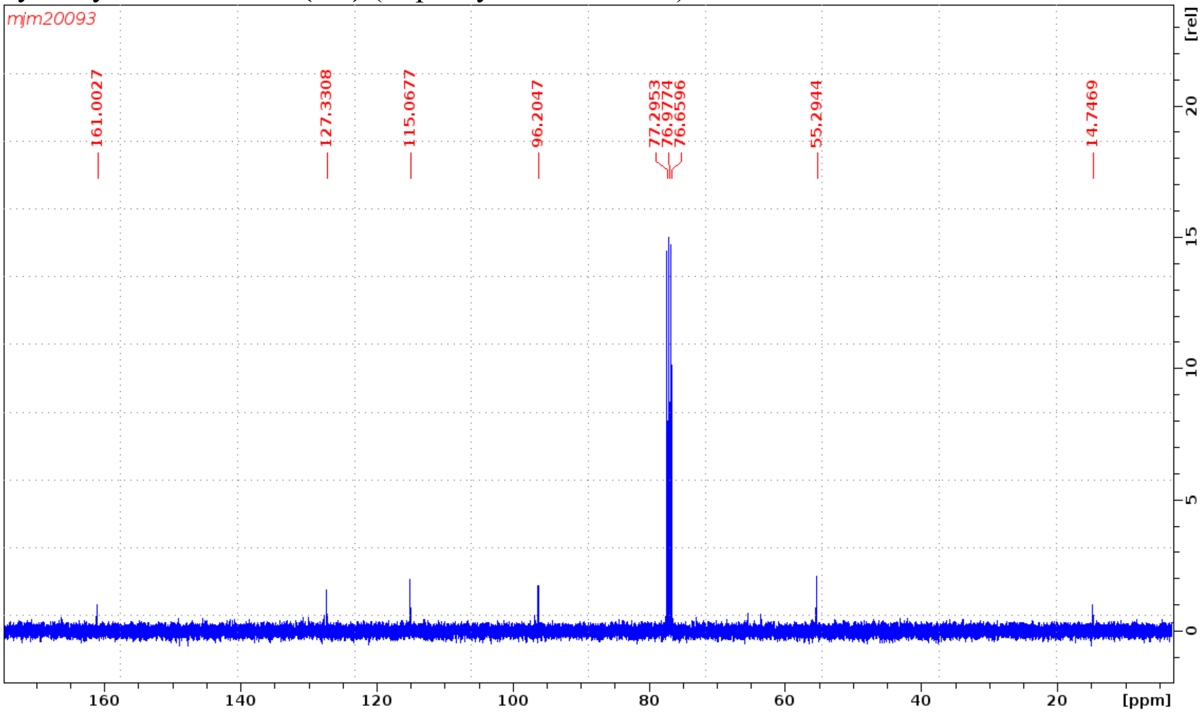

Figure S40: ^{13}C NMR Spectrum 1-(3,5-Dimethoxyphenyl)-4-(4-ethoxyphenyl)-3-hydroxyazetidin-2-one **(12l)**

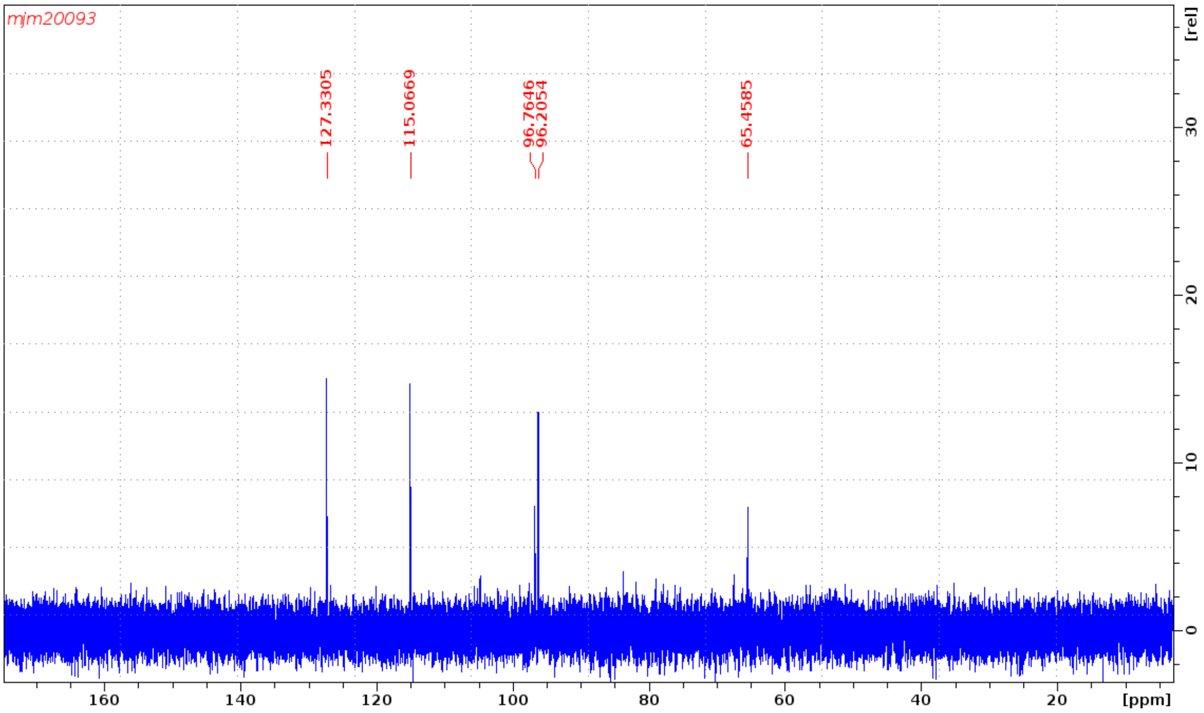

Figure S41: ^{13}C NMR DEPT-90 Spectrum 1-(3,5-Dimethoxyphenyl)-4-(4-ethoxyphenyl)-3-hydroxyazetidin-2-one **(12l)**

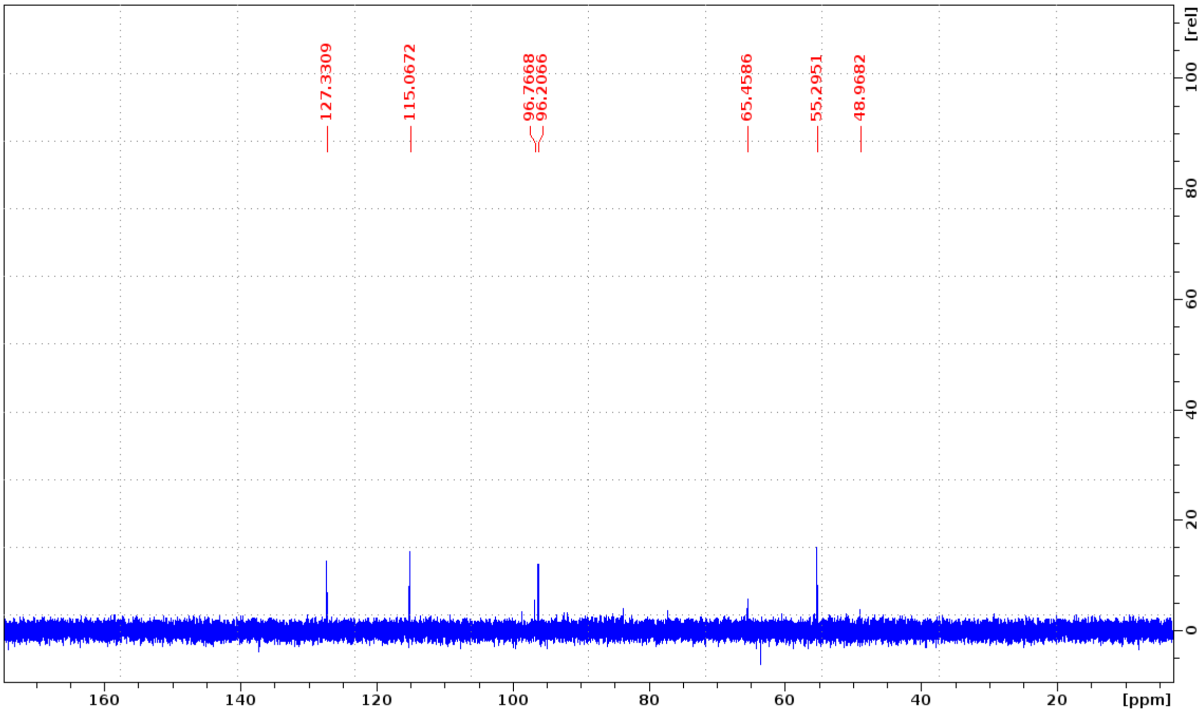

Figure S42: ^{13}C NMR DEPT-135 Spectrum 1-(3,5-Dimethoxyphenyl)-4-(4-ethoxyphenyl)-3-hydroxyazetidin-2-one **(12l)**

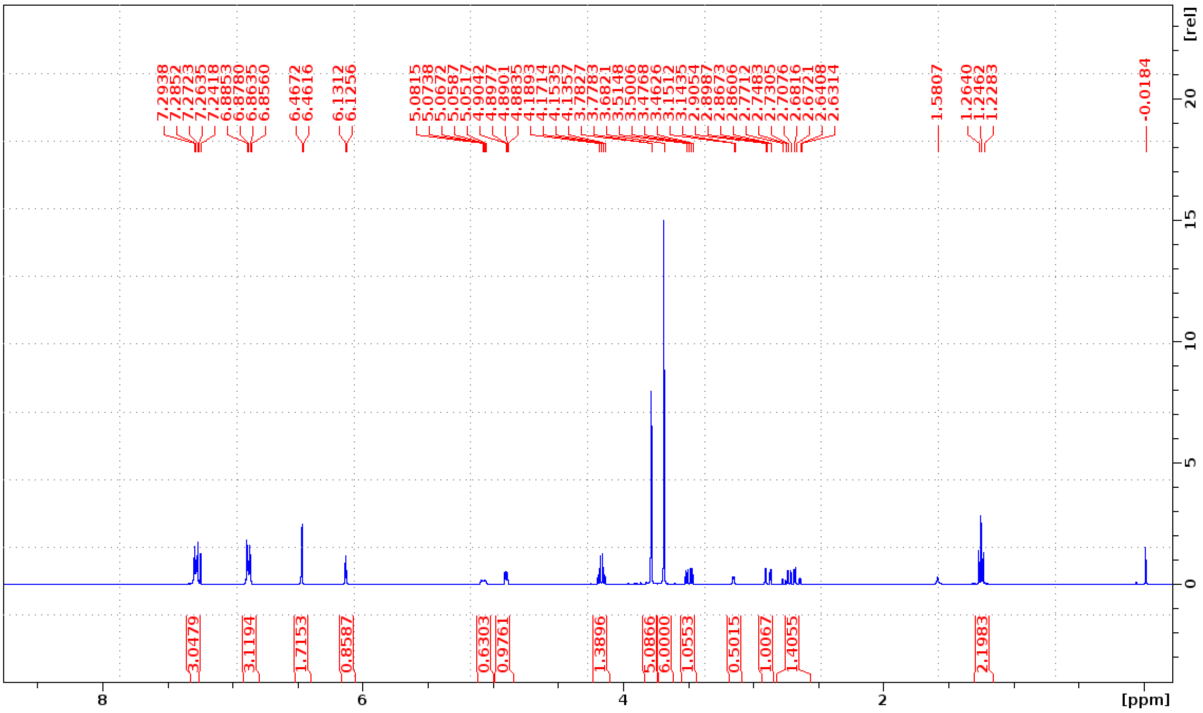

Figure S43: ^{1}H NMR Spectrum 1-(3,5-Dimethoxyphenyl)-4-(4-methoxyphenyl)azetidin-2-one **(12o)** (impurity H_2O at δ 1.58)

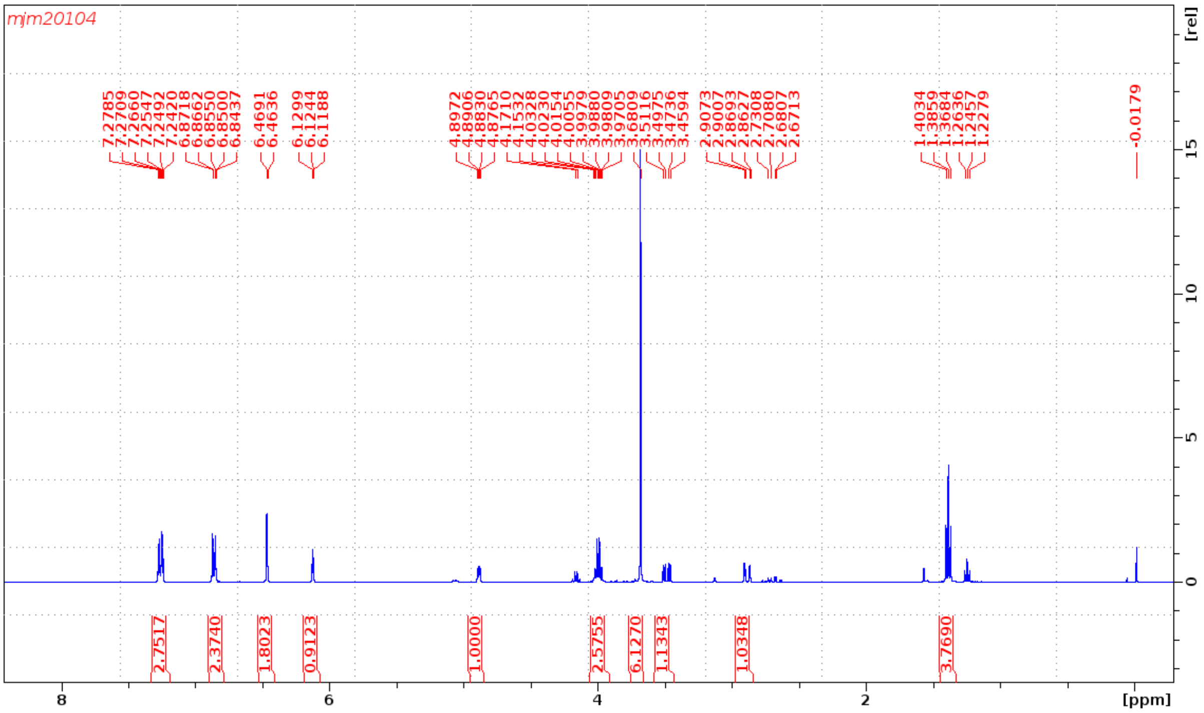

Figure S44: ^{1}H NMR Spectrum 1-(3,5-Dimethoxyphenyl)-4-(4-ethoxyphenyl) azetidin-2-one (**12p**) [δ 1.58 (impurity H_2O), δ 1.24, δ 2.70, δ 4.15]

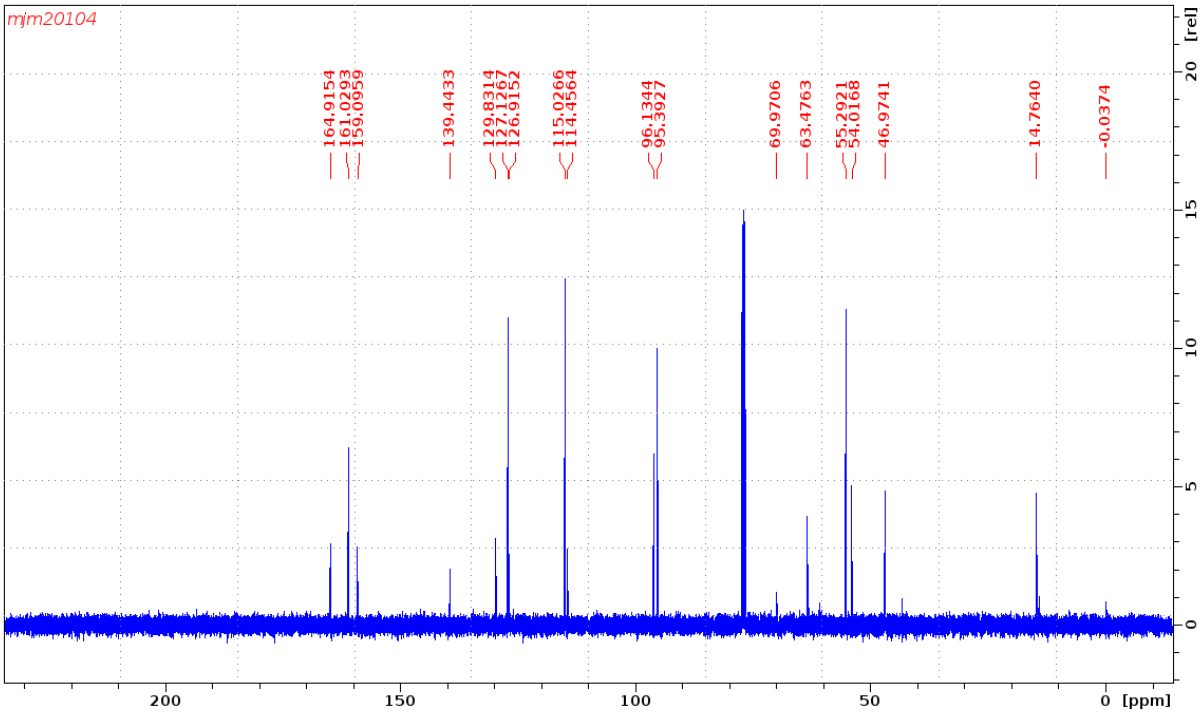

Figure S45: ^{13}C NMR Spectrum 1-(3,5-Dimethoxyphenyl)-4-(4-ethoxyphenyl) azetidin-2-one **(12p)**

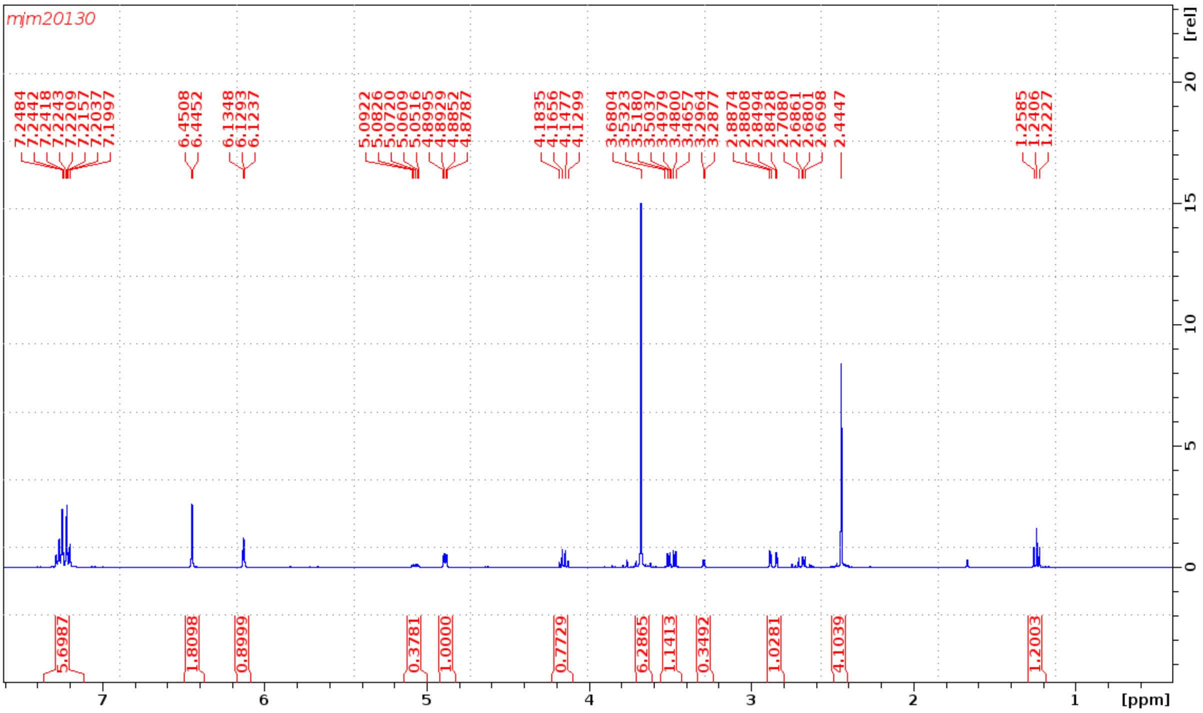

Figure S46: ^{1}H NMR Spectrum 1-(3,5-Dimethoxyphenyl)-4-(4-(methylthio)phenyl)azetidin-2-one **(12q)** [impurity H_2O at δ 1.58]

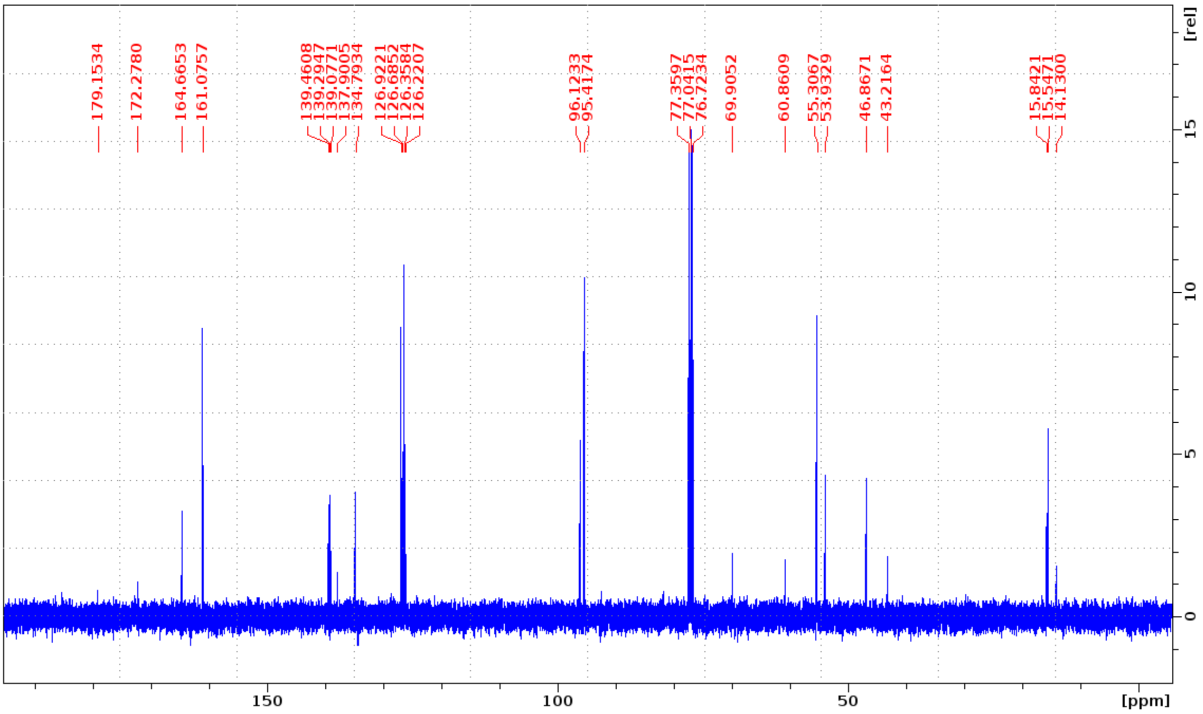

Figure S47: ^{13}C NMR Spectrum 1-(3,5-Dimethoxyphenyl)-4-(4-(methylthio)phenyl)azetidin-2-one **(12q)**

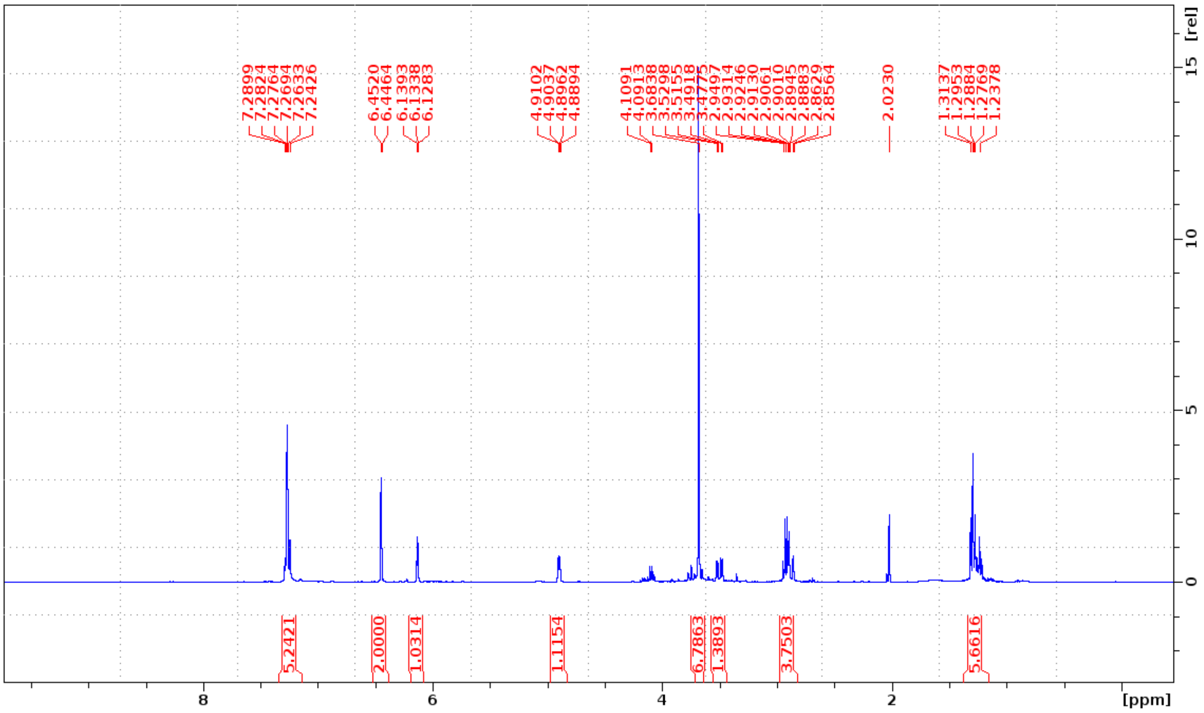

Figure S48: ^{1}H NMR Spectrum 1-(3,5-Dimethoxyphenyl)-4-(4-(ethylthio)phenyl)azetidin-2-one **(12r)** [impurity acetone at δ 2.02, ethyl acetate δ 1.24, δ 2.02, δ 4.10]

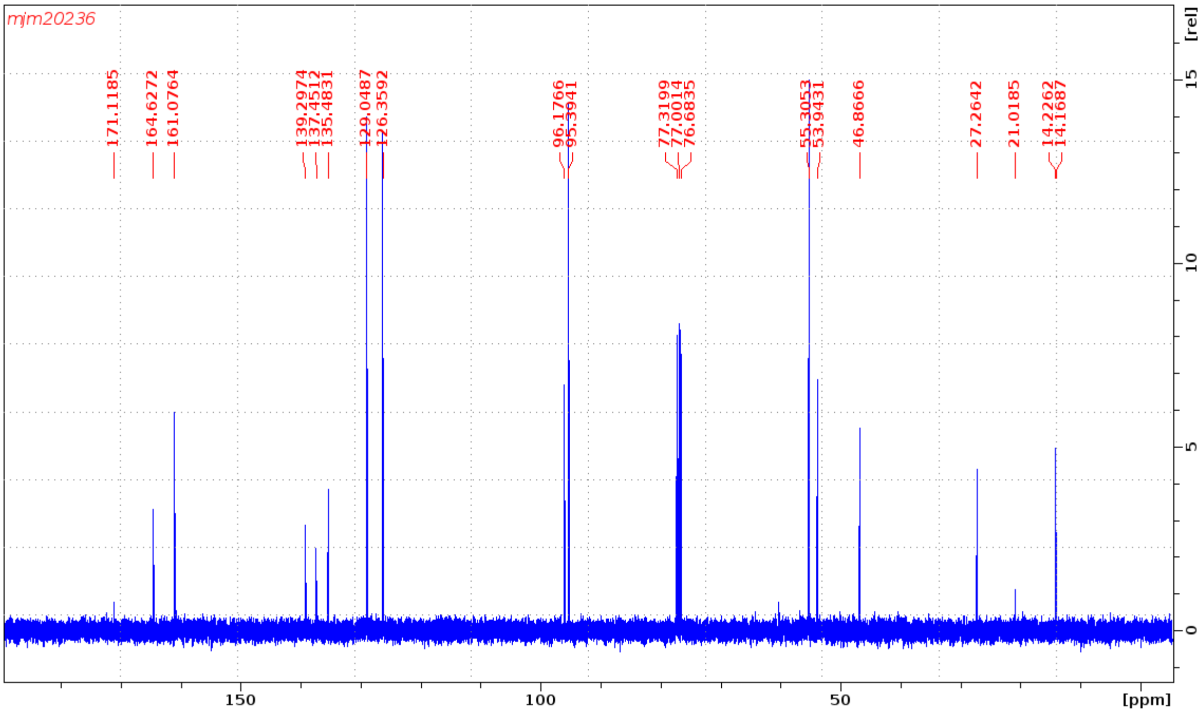

Figure S49: ^{13}C NMR Spectrum 1-(3,5-Dimethoxyphenyl)-4-(4-(ethylthio)phenyl)azetidin-2-one **(12r)**

Hirshfeld Surface analysis.

Images show the normalized contact distance (dnorm) surface with red areas indicating shorter than VDW radii and white-blue greater than VDW radii. Fingerprint plots are *di* (internal distance) plotted *vs de* (external distance) with reciprocal contacts shown.

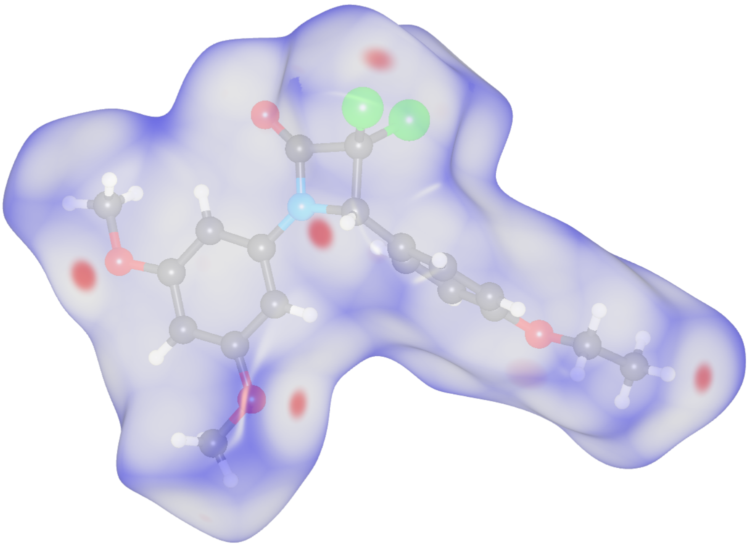

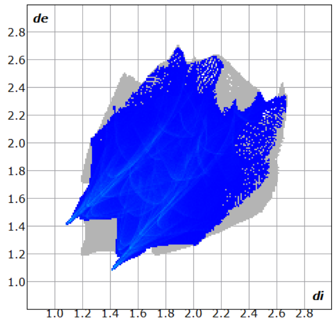

A B

Figure S50. (A) Hirshfeld surface of **12i** showing predominantly weak C-H…O (lactam-CH to methoxide) and Cl…O interactions and (B) fingerprint plot showing the C-H…O interactions, 21.5% of the surface area contacts.

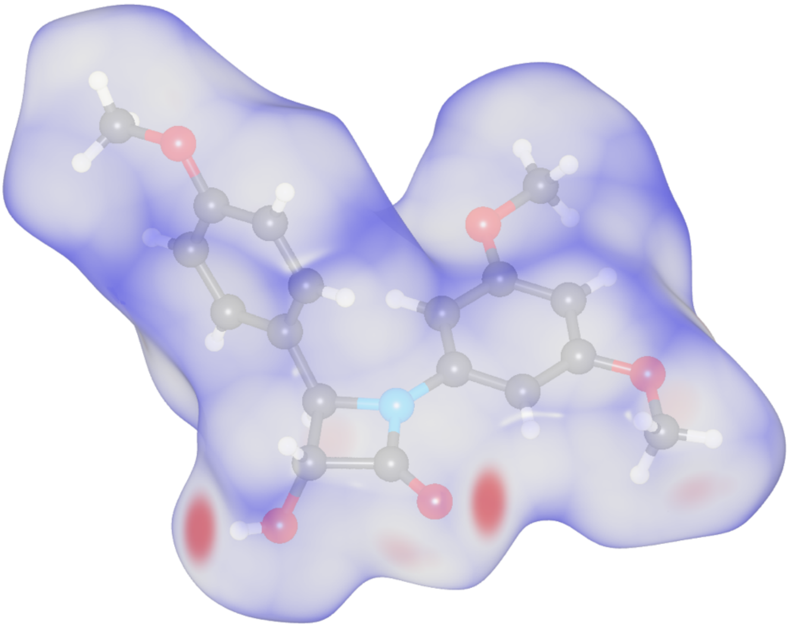

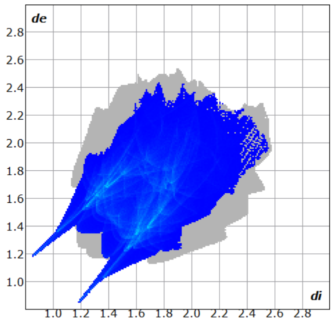

A B

Figure S51. (A) Hirshfeld surface of **12k** showing predominant OH…O interactions and (B) fingerprint plot showing the O-H…O interactions, 29.3% of the surface area contacts.

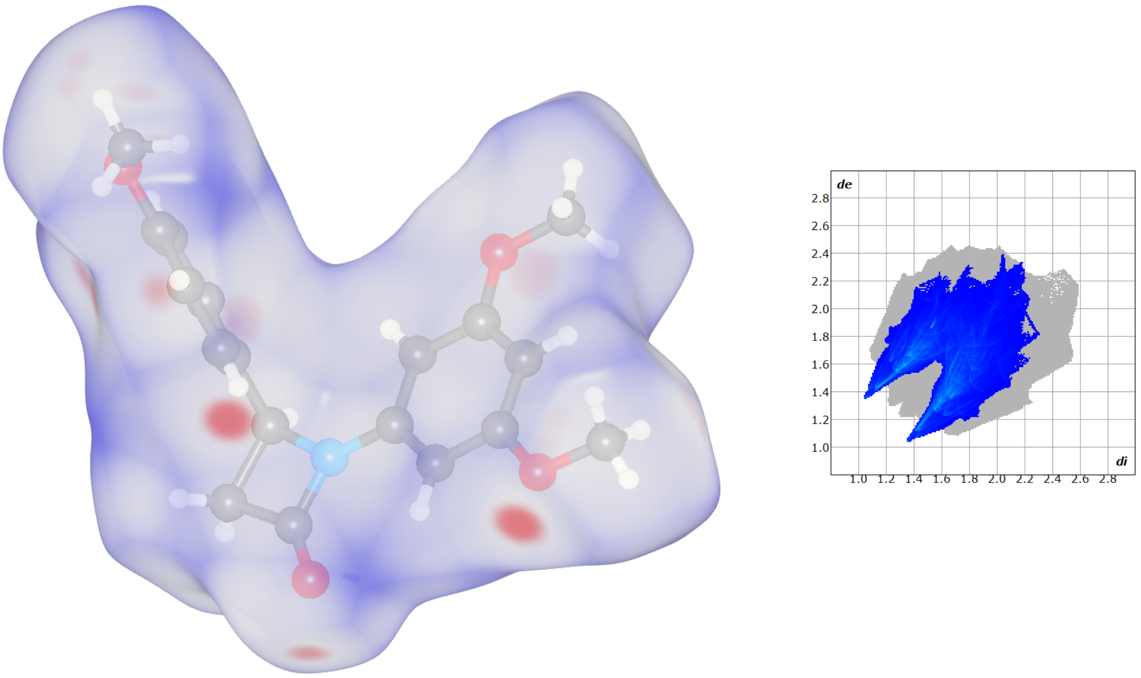

A B

Figure S52. (A) Hirshfeld surface of **12o** showing predominant weak CH…O (aryl ring – methoxide) interactions and (B) fingerprint plot showing the C-H…O interactions, 23.7% of the surface area contacts.

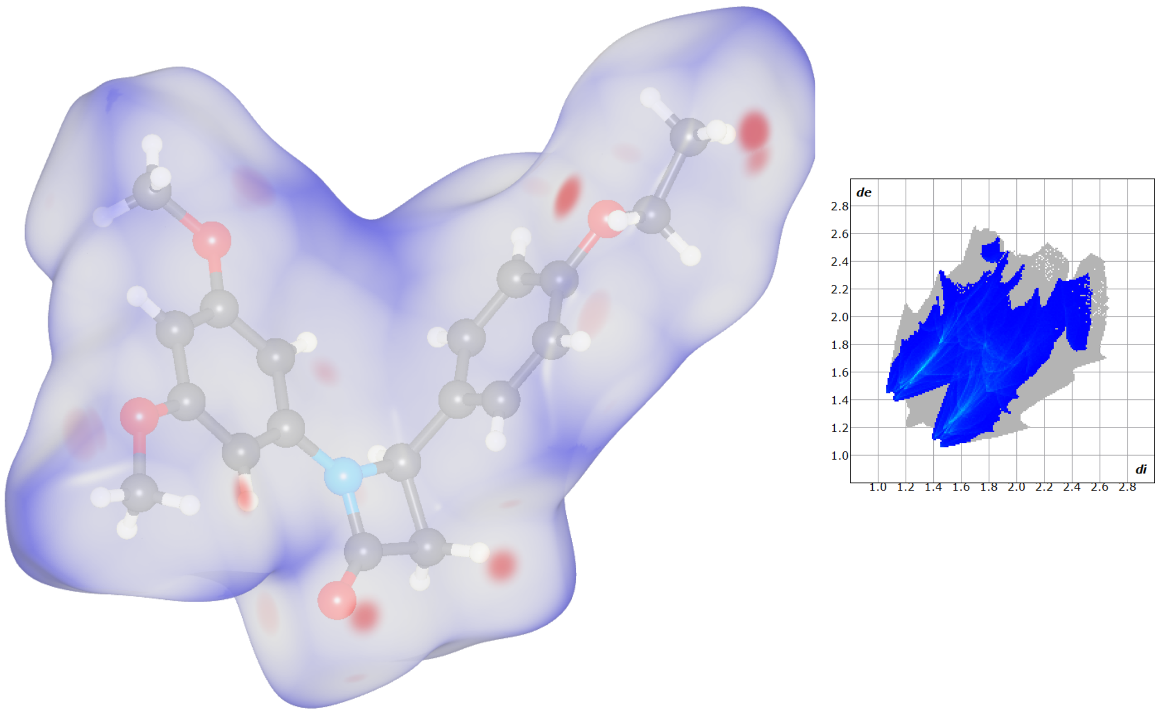

A B

Figure S53. (A) Hirshfeld surface of **12p** showing predominant weak CH…O interactions (Methoxide-CH3…methoxide, ethoxide-CH3…methoxide; lactam-CH2…ketone) and (B) fingerprint plot showing the C-H…O interactions, 22.7% of the surface area contacts.

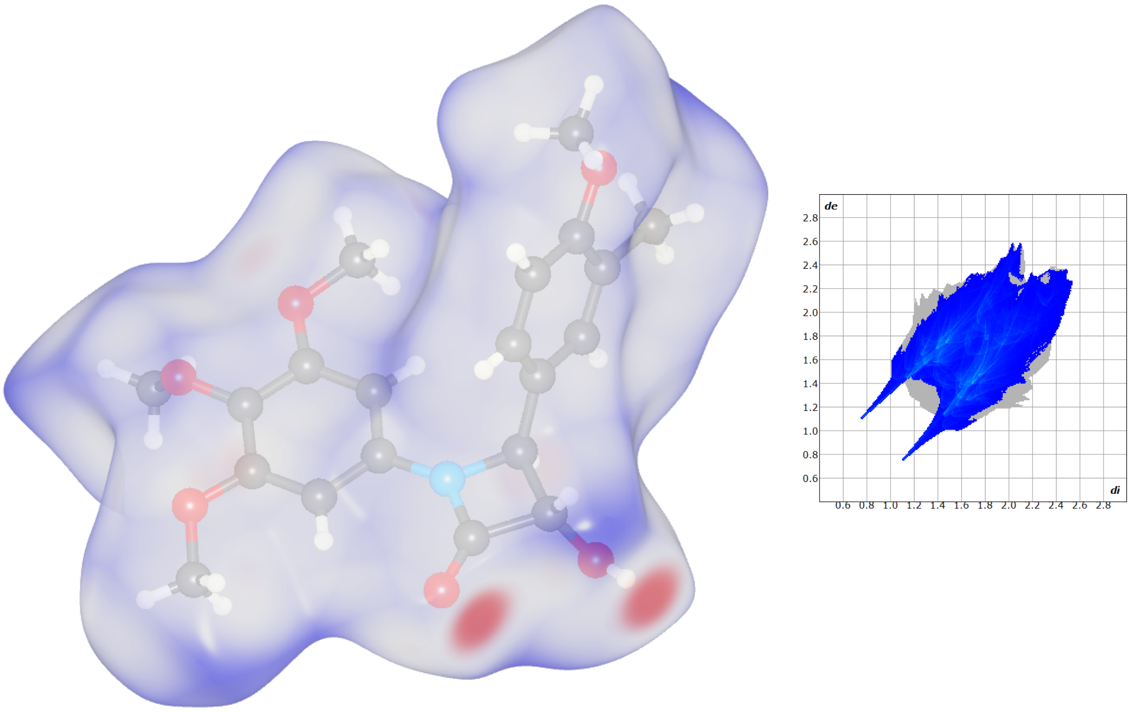

A **B**

Figure S54. (A) Hirshfeld surface of **12u** showing predominant OH…O interactions (lactam OH ….ketone O) and (B) fingerprint plot showing the O-H…O interactions, 30.4% of the surface area contacts.

References

1. Promega corporation, cytotox 96® non-radioactive cytotoxicity assay; promega cytotox 96 nonradioactive cytotoxicity assay protocol, 2016. Https://worldwide.Promega.Com/products/cell-health-assays/cell-viability-and-cytotoxicity-assays/cytotox-96-non_radioactive-cytotoxicity-assay/?Catnum=g1780 (accessed 06 February 2025).
2. Wang, S.; Malebari, A.M.; Greene, T.F.; O'Boyle, N.M.; Fayne, D.; Nathwani, S.M.; Twamley, B.; McCabe, T.; Keely, N.O.; Zisterer, D.M., *et al.* 3-vinylazetidin-2-ones: Synthesis, antiproliferative and tubulin destabilizing activity in MCF-7 and MDA-MB-231 breast cancer cells. *Pharmaceuticals (Basel)* **2019**, *12*.
